# Efficient cancer modeling through CRISPR-Cas9/HDR-based somatic precision gene editing in mice

**DOI:** 10.1126/sciadv.ade0059

**Published:** 2023-05-12

**Authors:** Wen Bu, Chad J. Creighton, Kelsey S. Heavener, Carolina Gutierrez, Yongchao Dou, Amy T. Ku, Yiqun Zhang, Weiyu Jiang, Jazmin Urrutia, Wen Jiang, Fei Yue, Luyu Jia, Ahmed Atef Ibrahim, Bing Zhang, Shixia Huang, Yi Li

**Affiliations:** ^1^ Lester and Sue Smith Breast Center, Baylor College of Medicine, Houston, TX, USA.; ^2^ Department of Medicine, Baylor College of Medicine, Houston, TX, USA.; ^3^ Dan L. Duncan Comprehensive Cancer Center, Baylor College of Medicine, Houston, TX, USA.; ^4^ Department of Molecular and Human Genetics, Baylor College of Medicine, Houston, TX, USA.; ^5^ Department of Molecular and Cellular Biology, Baylor College of Medicine, Houston, TX, USA.; ^6^ Department of Education, Innovation, and Technology, Baylor College of Medicine, Houston, TX, USA.

## Abstract

CRISPR-Cas9 has been used successfully to introduce indels in somatic cells of rodents; however, precise editing of single nucleotides has been hampered by limitations of flexibility and efficiency. Here, we report technological modifications to the CRISPR-Cas9 vector system that now allows homology-directed repair–mediated precise editing of any proto-oncogene in murine somatic tissues to generate tumor models with high flexibility and efficiency. Somatic editing of either *Kras* or *Pik3ca* in both normal and hyperplastic mammary glands led to swift tumorigenesis. The resulting tumors shared some histological, transcriptome, and proteome features with tumors induced by lentivirus-mediated expression of the respective oncogenes, but they also exhibited some distinct characteristics, particularly showing less intertumor variation, thus potentially offering more consistent models for cancer studies and therapeutic development. Therefore, this technological advance fills a critical gap between the power of CRISPR technology and high-fidelity mouse models for studying human tumor evolution and preclinical drug testing.

## Teaser

Cancer can be modeled through somatic precision gene editing with high flexibility and efficiency.

## INTRODUCTION

Preclinical cancer animal models play critical roles in investigating genes in cancer development and progression, as well as preclinical testing of cancer prevention and therapeutics. Commonly used mouse models include genetically engineered mouse models (GEMMs) and virus-mediated oncogene delivery mouse models (*1*). As these models are immunocompetent, they are especially valuable for studying the involvement of the immune system, a key advantage over xenograft models. However, GEMMs generally introduce genetic alterations into the germ line, often leading to genetic and expression changes before the target organ is fully developed and usually inflicting tissue-wide impact even when conditional or inducible tools are added on (*1*). Therefore, these models do not closely mimic the development of most sporadic cancers in humans. To circumvent these shortcomings, we and others have used retrovirus and lentivirus to carry mutated genes (or Cre for deleting loxP-marked genes) into a small number of somatic cells in selected tissues at selected times, therefore affording both temporal and spatial control of oncogenic drivers (*1*–*5*). However, this viral approach inserts mutated genes under the control of exogenous promoters [usually a viral long terminal repeat (LTR)]; therefore, these virus-delivered exogenous genes lack their native gene expression controls. Furthermore, retrovirus and lentivirus integrate into the infected cell genome unscrupulously, causing unpredictable copy number and integration effects and even disrupting endogenous genes if the viruses integrate into their gene body or regulator regions (*1*).

The CRISPR-Cas9 system provides an opportunity to edit endogenous genes in their native loci (*6*–*8*), therefore overcoming drawbacks of virus-mediated oncogene delivery models. CRISPR editing can generate both indel and point mutations. Multiple mouse tumor models have been successfully generated by somatically indel-editing tumor suppressor genes mediated by Cas9-catalyzed, guide RNA (gRNA)–directed double-strand breaks at specific genomic locations followed by error-prone nonhomologous end joining (NHEJ) repair leading to insertions and deletions and subsequent loss of function (*6*–*8*). However, most cancer-causing genetic alterations in humans are point mutations (*9*), which often result in amino acid alterations that can markedly affect protein activities. Installing missense mutations in proto-oncogenes can be achieved by several Cas9-based methods (*6*). The deaminase-mediated base-editing approach can introduce transition mutations—C:G to T:A base editing (CBE) or A:T to G:C base editing using a nuclease-deficient Cas9 fused with a cytidine or adenine deaminase. The C:G to G:C transversion mutation has also been achieved by recently developed variant enzymes (*10*, *11*). So far, only the CBE technique has been used successfully for tumor modeling in vivo (*12*, *13*). However, this editing method requires the protospacer adjacent motif (PAM) sequence being present at the location 12 to 18 nucleotides 3′ to the target base, and it currently cannot perform 6 of 12 possible base changes. Furthermore, as a specific deaminase cannot differentiate between the base intended to edit and the neighboring bases of the same identity, this method often introduces bystander mutations proximal to the targeted nucleotide within the base-editing window (*12*). The recently developed prime editor is composed of a Cas9 nickase fused with a reverse transcriptase and a prime editing guide RNA (pegRNA), which provides both gRNA and a template for reverse transcription (*6*). This method has been used to successfully introduce the S45F mutation into β-catenin and to induce liver tumors in mice (*14*). The experiment was carried out by hydrodynamic tail vein injection of three independent components, Cas9, pegRNA, and another single-guide RNA for improving editing efficiency. Although this method can theoretically introduce any point mutation, the delivery method is still impractical for modeling cancers in most organs.

While NHEJ following gRNA/Cas9-initiated double-strand break causes indels, coupling homology-directed repair (HDR) to gRNA/Cas9 can introduce all 12 point mutations (*6*). Including a short fragment of HDR donor sequence with engineered point mutations in the gRNA vector has led to successful precise edits in cell cultures [reviewed in (*6*, *8*)]. However, using Cas9-mediated HDR to somatically edit proto-oncogenes for cancer modeling in vivo has not been very successful (*15*, *16*). For example, Platt *et al.* (*15*) designed an editing vector for both indel-editing of the *Tp53* and *Lkb1* tumor suppressor genes and installing a missense mutation in the *Kras* proto-oncogene in somatic respiratory epithelium to model lung cancer. While polymerase chain reaction (PCR) sequencing of the edited region of the resulting tumors detected tumor-driver levels of indel mutations of *Tp53* and *Lkb1*, the *KrasG12D* missense mutation could hardly be detected. This is contradictory to the well-known strong cooperation between KRASG12D and the loss of function of P53 or LKB1 in mouse lung tumorigenesis models (*17*), indicating that HDR-based *Kras* editing may not be very successful. Another group (*18*) used a similar vector that also targeted these three genes to build lung cancer mouse models, but they used PCR with primers specific for the mutated *Kras* to assess *Kras* mutations in their tumors. Activating *Kras* mutations could not be detected in over one quarter of the approximate 50 tumors tested, and in the tumors which showed a positive PCR signal, whether the point mutation occurred in any appreciable fractions of the tumor cell population could not be determined (*18*). Therefore, tumor induction by genome editing of a single proto-oncogene in vivo in the absence of other oncogenic alterations has not been robustly demonstrated, and technical improvement of CRISPR-Cas9/HDR–based gene editing is needed to fully harness its power for precision editing in tumor modeling.

We report here a modified CRISPR-Cas9/HDR–based method that can somatically edit proto-oncogenes with high flexibility and efficiency to generate tumors in mouse mammary glands. Furthermore, autochthonous tumor models generated by this new method show some remarkable advantages compared to those generated by virus-mediated oncogene delivery methods. We expect this improved method to be broadly useful for precision editing in other somatic tissues for cancer modeling.

## RESULTS

### An AAV vector system edits *Kras* in mouse mammary glands and induces tumors with high efficiency

**Fig. 1.**
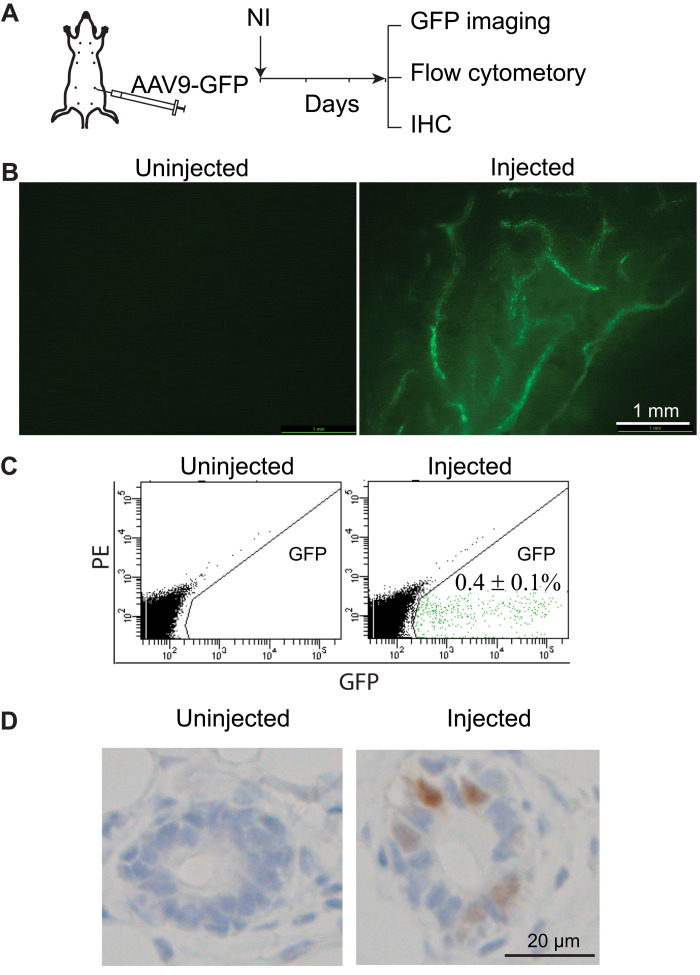
AAV serotype 9 infects mammary gland epithelial cells. (**A**) Diagram of experimental design to test the infection capacity of AAV serotype 9. (**B**) Mammary duct tree 3 days after intraductal infection by AAV9 carrying *copGFP*, imaged under a fluorescent stereoscope. (**C**) Detection of AAV9-GFP–infected cells using flow cytometry. PE, phycoerythrin. (**D**) Detection of AAV9-GFP–infected cells using immunohistochemistry (IHC).

We wanted to improve the tumor modeling of CRISPR-Cas9/HDR–based somatic gene editing in mammary glands and then compare it with our previously used lentivirus/retrovirus-mediated oncogene delivery mammary tumor modeling (*2*, *19*–*21*). Since gRNA and the HDR sequence are needed only briefly in cells to edit genes and we want to avoid the vector integration effects, we chose an adeno-associated virus (AAV) vector instead of retroviral or lentivirus vectors, which insert genes permanently into the host genome. There are multiple serotypes of AAVs with different tissue tropisms, and AAV serotypes 1 and 9 have been reported to infect mouse mammary gland epithelial cells (*22*). To confirm this and determine the infection efficiency, we injected AAV-9 carrying *copGFP* (AAV9-GFP) intraductally into mammary glands of mice (~1 × 10^11^ genome copies (gc) per gland; Fig. 1A). Three days later, green fluorescence was readily detected in multiple ducts under a fluorescent stereomicroscope (Fig. 1B). Flow cytometry of dissociated mammary cells prepared from infected glands showed ~0.4% of infected cells (Fig. 1C). For comparison, intraductal injection of 7.5 × 10^5^ infectious units of Lenti–green fluorescent protein (GFP) led to infection of approximately 4% of mammary cells (*20*). Immunohistochemistry staining of copGFP also confirmed the infection of epithelial cells (Fig. 1D). Together, these data confirm that serotype 9 AAV is suitable for mammary gland epithelial infection.

**Fig. 2.**
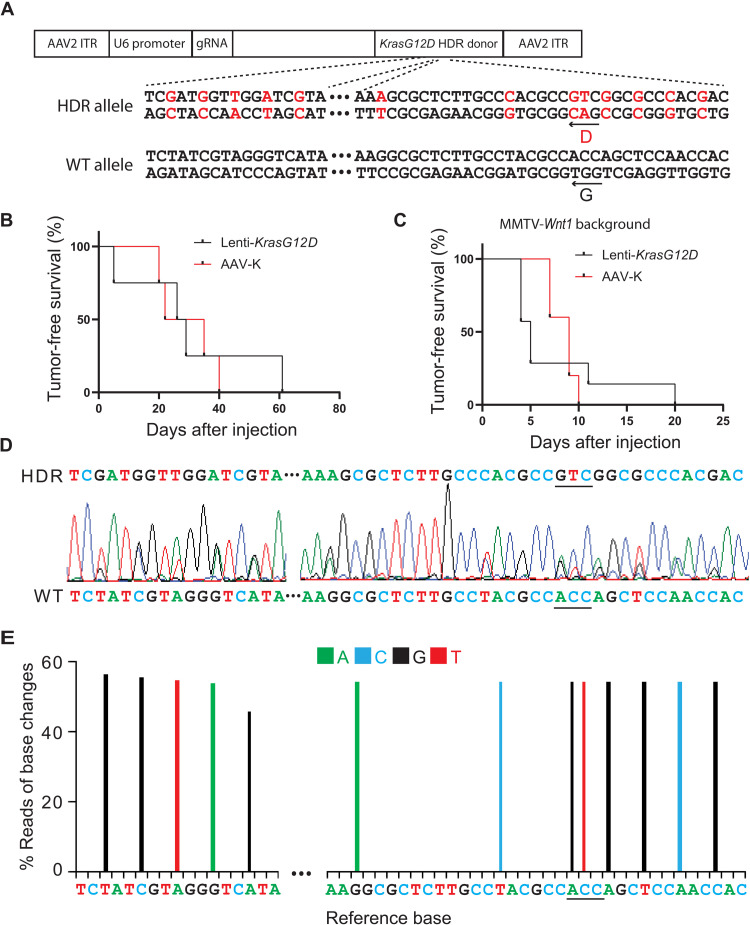
Installing *KrasG12D* mutation through somatic editing efficiently induces tumors in mouse mammary glands. (**A**) Diagram of the AAV-K construct that carries gRNA and HDR donor to install a *KrasG12D* mutation. Both HDR donor sequence and wild-type (WT) sequence are shown. Shown in red are G12D and synonymous mutations. ITR, inverted terminal repeat. (**B**) Kaplan-Meier tumor-free survival curves of CAG-SpCas9-P2A-EGFP mice with one #4 gland intraductally injected with AAV-K (5.0 × 10^11^ gc) or Lenti-*KrasG12D* (3.8 × 10^7^ IUs). (**C**) Kaplan-Meier tumor-free survival curves of CAG-*SpCas9-P2A-EGFP*/MMTV-*Wnt1* mice with one #4 gland intraductally injected with AAV-K or Lenti-*KrasG12D*. (**D**) A representative Sanger sequencing chromatogram of the *Kras* locus of an AAV-K–induced mammary tumor in a CAG-*SpCas9-P2A-EGFP* mouse. (**E**) A representative graph of percentage of base change reads from Amplicon Next-Generation sequencing of the *Kras* locus of an AAV-K–induced mammary tumor in a CAG-*SpCas9-P2A-EGFP* mouse. The color code of the changed bases is shown above the graph.

**Fig. 3.**
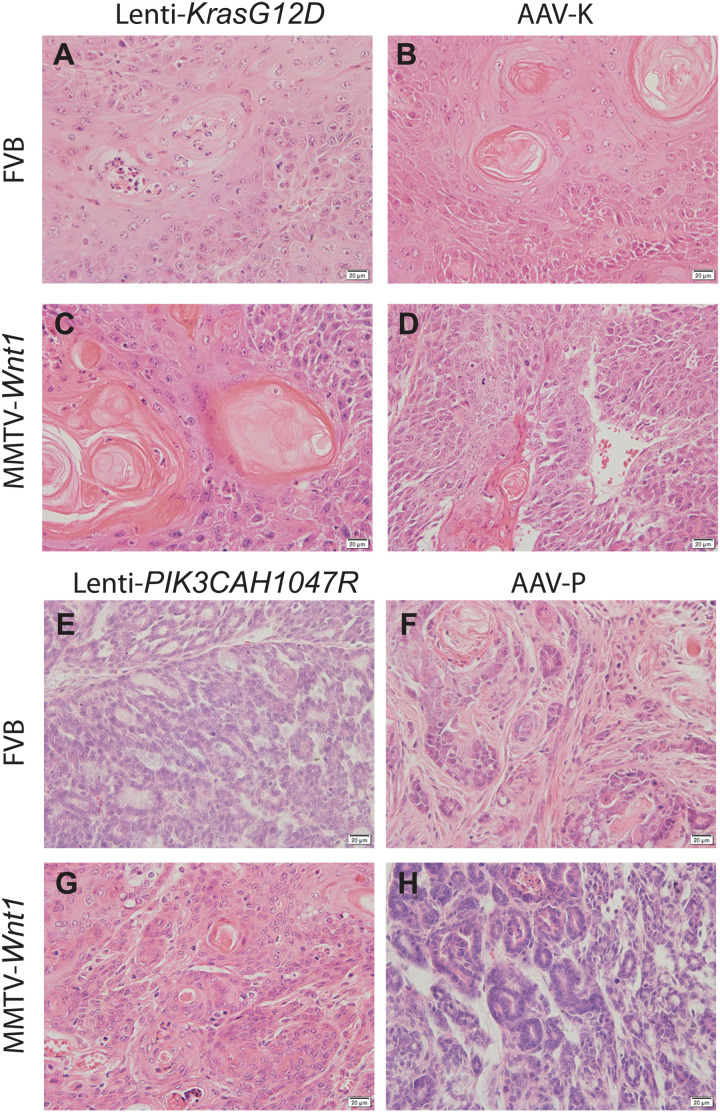
Histology of mammary tumors induced by oncogene lentivirus delivery method and gene editing method. Representative photomicrographs of hematoxylin and eosin (H&E)–stained mammary tumors. The viruses used and the genetic background are indicated. The left panels (**A**, **C**, **E**, and **G**) are tumors induced by lentivirus delivery method. The right panels (**B**, **D**, **F**, and **H**) are tumors induced by gene editing method.

To determine whether AAV9 could be used to introduce gRNA and HDR to edit a proto-oncogene in mammary glands to initiate tumorigenesis, we selected *Kras*, which is the most frequently mutated proto-oncogene in human cancers (*23*). There are two additional reasons to choose this oncogene: (i) Viral vector-mediated delivery of a mutated member of the *Ras* family (*Kras*, *Hras*, or *Nras*) has been reported to cause mammary tumors in mice and rats swiftly (*1*, *19*, *24*); (ii) we wanted to compare our result with the previous Platt *et al.* (*15*) report on somatic editing of *Kras* to generate lung tumors. In that study, the authors used both a conditional activated Cas9 transgenic mouse line and an AAV vector (AAV-KPL) that carried the luciferase reporter and Cre in addition to gRNAs (targeting *Tp53*, *Lkb1*, and *Kras*) and the HDR donor sequence for *KrasG12D* to model lung cancer by causing indel mutations of tumor suppressor genes *Tp53* and *Lkb1* and missense mutation of *Kras*, but the resulting tumors only showed a negligible level of *KrasG12D* missense mutations. The reasons for the low levels of success in introducing the designed point mutation could be several, so we decided to take this published system and make multiple changes to improve our chance of success. First, we chose to forgo a conditional *Cas9* expression mouse line so that we can avoid somatic activation of Cas9, which can be highly immunogenic (*25*, *26*), and instead selected a Cas9 mouse line that expresses *Cas9* constitutively so that Cas9 becomes a self-antigen. The selection of this germline Cas9 line also eliminated the need for *Cre* in the viral vector, avoiding another immunogen [although perhaps a modest one (*27*)]. While deleting the *Cre* promoter in AAV-KPL, we also deactivated the linked *Luciferase* gene, thus removing one more immunogen (*28*). Somatic expression of these immunogens in studies by Platt *et al.* (*15*) did not block mutated *Tp53* and *Lkb1* from causing lung carcinoma but perhaps inflamed the modest immune surveillance against the subset of cells that were additionally edited to produce *KrasG12D*, which itself is a neoantigen (*29*), leading to a negative selection against these triple-mutated cells in the final tumors reported by Platt *et al.* (*15*). Besides disabling the expression of *Luciferase* and *Cre*, this vector modification also prevented the transcription machinery from running into the immediate downstream HDR region, which may impair this region’s function as the homologous recombination donor. Furthermore, we also removed the gRNAs targeting *Tp53* and *Lkb1* in AAV-KPL so that we can specifically test the outcome of the edited *Kras* (Fig. 2A). Intraductal injection of the resulting virus (AAV-K; 5.0 × 10^11^ gc per gland), packaged in an AAV9 capsid, into four 6- to 8-week-old CAG-SpCas9-P2A-EGFP transgenic mice led to mammary tumors in all mice with a swift tumor latency of 28.5 days, comparable to the latency observed by intraductal injection of Lenti-*KrasG12D* [3.8 × 10^7^ international units (IUs), which likely infected more cells based on our previous study of the infection efficiency of lentivirus (*20*); Fig. 2B], indicating a high efficiency of genome editing for tumor modeling. At the ethical end point of approximately 2.0 cm in diameter, both groups of tumors are high-grade adeno-squamous carcinoma sprouting invasive nests of tumor cells, but the AAV-K group (*n* = 4) appears to have progressed further into squamous carcinoma with extensive keratin production and little or no glandular presence compared to the Lenti-*KrasG12D* cohort (*n* = 8; Fig. 3, A and B), suggesting that edited *Kras* and overexpressed *KrasG12D* may drive tumor cell differentiation differently.

To verify that these tumors were caused by precision editing of *Kras*, we first sequenced the amplicon of the edited region through Sanger sequencing. Besides the G12D mutation, the *KrasG12D* HDR donor also includes multiple synonymous mutations to prevent gRNA from recognizing the initial recombination product, the *Kras^HDR^* allele, and to distinguish the genome-edited allele from a potential spontaneously mutated allele. As shown in Fig. 2D, all intentionally edited nucleotides were detected as a major second peak. To further quantify the editing efficiency, we conducted the next-generation amplicon sequencing of the edited region. The sequencing result showed that ~50% of reads carried the expected *KrasG12D* mutation and the silent mutations introduced by the donor DNA in AAV-K (Fig. 2E), confirming that this genome editing system can efficiently edit proto-oncogenes and induce tumorigenesis in the mouse mammary gland.

Next, we tested whether AAV-K could also be used to efficiently edit *Kras* in precancerous cells that had already suffered other oncogenic alterations, for two reasons: (i) We wanted to ascertain that our vector system could also efficiently edit genes in precancerous cells, since the initial report of poor *Kras* editing by Platt *et al.* (*15*) came from experiments in cells that gained other oncogenic drivers that drove tumorigenesis; and (ii) proto-oncogenes are often activated as secondary or tertiary oncogenic events in atypical cells to accelerate the progression to cancer. *Kras* or *Hras* is known to be spontaneously activated in precancerous mammary epithelial cells to instigate tumor formation in transgenic models of breast cancer, including in mice transgenic for *Wnt1* under the control of mouse mammary tumor virus (MMTV) LTR (*30*, *31*). We have reported that intraductal injection of lentivirus carrying either *HrasQ61L* or *KrasG12D* was sufficient to transform precancerous mammary cells in MMTV-*Wnt1* mice to tumors (*19*). Therefore, we tested whether AAV-K was also sufficient to cause cancer in precancerous mammary cells in MMTV-*Wnt1* mice. We intraductally injected 5- to 8-week-old mice bitransgenic for MMTV-*Wnt1* and CAG-*SpCas9-P2A-EGFP* with AAV-K packaged in AAV9 serotype (5 × 10^11^ gc per gland). We palpated mammary tumors in these mice with a median latency of only 9 days, comparable to the rapid tumor detection in MMTV-*Wnt1* mice injected with Lenti-*KrasG12D* (Fig. 2C). Both groups of tumors are high-grade adeno-squamous carcinoma sprouting invasive nests of tumor cells, but the AAV-K group (*n* = 5) shows more adeno-differentiation admixed with a lesser squamous component than the Lenti-*KrasG12D* cohort (*n* = 5) (Fig. 3, C and D), opposite to the tumor differences in FVB mice, suggesting that the precancerous state affects edited *Kras* versus overexpressed *KrasG12D* in specifying tumor characteristics. These AAV-K–induced tumors in MMTV-*Wnt1* mice exhibited high histological similarities to spontaneous tumors arising in noninjected MMTV-*Wnt1* transgenic mice, while Lenti-*KrasG12D*–induced tumors did not, suggesting that precision editing mimics natural tumor evolution—which often involves spontaneous activating mutations of *Hras* in this transgenic Wnt1 model (*31*)—more closely than virus-introduced ectopic expression of oncogenes. Together, these data from both normal and precancerous mice indicate that our modified approach can efficiently install missense mutation of a proto-oncogene to drive tumorigenesis and may generate tumor models that closely mimic natural tumor evolution.

### Genome-edited *Pik3ca* induces mammary tumors with high efficiency

**Fig. 4.**
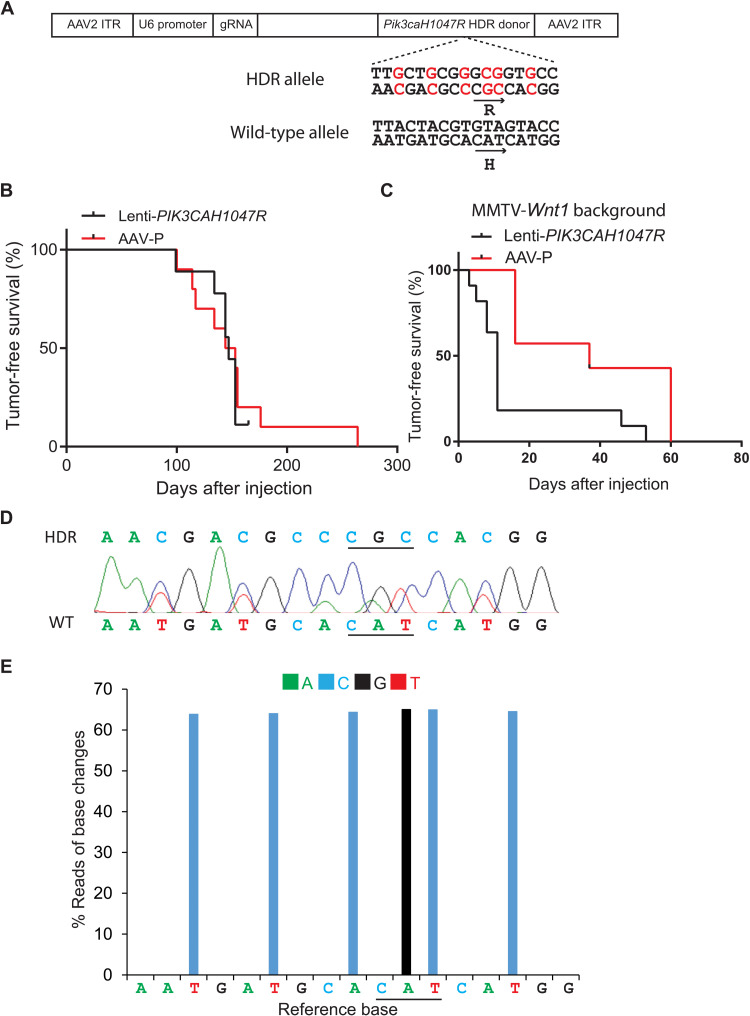
Installing *Pik3caH1047R* mutation through somatic editing efficiently induces tumors in mammary glands. (**A**) Diagram of the AAV-P construct that carries gRNA and HDR donor to install a *Pik3caH1047R* mutation. H1047R mutation and synonymous mutations are shown in red. (**B**) Kaplan-Meier tumor-free survival curves of CAG-SpCas9-P2A-EGFP mice intraductally injected into one #4 gland with AAV-P (5.3 × 10^11^ gc) or Lenti-*PIK3CAH1047R* (1.5 × 10^7^ IUs). (**C**) Kaplan-Meier tumor-free survival curves of CAG-*SpCas9-P2A-EGFP*/MMTV-*Wnt1* mice intraductally injected with AAV-P or Lenti-*PIK3CAH1047R*. (**D**) A representative Sanger sequencing chromatogram of the *Pik3ca* locus in an AAV-P–induced mammary tumor in a CAG-*SpCas9-P2A-EGFP* mouse. (**E**) A representative graph of percentage of base change reads from Amplicon Next-Generation sequencing of the *Pik3ca* locus in an AAV-P–induced mammary tumor in a CAG-*SpCas9-P2A-EGFP* mouse. The color code of the changed bases is shown above the graph.

After having established that our HDR-based gene editing vector system could efficiently edit *Kras* and drive tumorigenesis, we tested whether our vector system could be broadly applicable for precision-editing in cancer modeling in mice. We selected *PIK3CA*, which, like *KRAS*, is also one of the commonly mutated proto-oncogenes in human cancers—in human breast cancer, it is mutated in 36% of all cases, the highest among mutated proto-oncogenes (*32*). Virus-mediated or transgenic expression of *PIK3CAH1047R*, the most common hot-spot mutant, leads to mammary tumors in mice (*33*–*37*). We replaced the gRNA and HDR in AAV-K with *Pik3ca* gRNA and HDR to cause the *H1047R* mutation, respectively. Similar to editing *Kras*, synonymous mutations were also included in this HDR. The resulting construct was named AAV-P (Fig. 4A). This construct was packaged with the AAV-9 serotype and then intraductally injected into mammary glands of 7- to 14-week-old CAG-*SpCas9-P2A-EGFP* transgenic mice (5.3 × 10^11^ gc per gland). Tumors were detected with a median latency of 3.9 months, comparable to the tumor latency in mice intraductally injected with a lentiviral vector carrying the *PIK3CAH1047R* (Lenti-*PIK3CAH1047R*, 1.5 × 10^7^ IUs; Fig. 4B). Both groups of tumors are low- to moderate-grade adenocarcinoma, but there are some notable histological differences. While the Lenti-*PIK3CAH1047R* group (*n* = 5) exhibits prominent papillary differentiation with minimum squamous differentiation as we reported previously (*33*), the AAV-P group (*n* = 6) shows less papillary and other glandular differentiation, exhibits notable focal squamous differentiation, and is thus less differentiated overall (Fig. 3, E and F), suggesting that similar to the *Kras* case, edited *Pik3ca* and overexpressed *PIK3CAH1047R* may also drive tumor cell differentiation differently. Both Sanger and deep sequencing of the amplicon surrounding the edited region demonstrated that these tumors carried the expected H1047R mutation and the synonymous mutations (Fig. 4, D and E).

The deep sequencing data of these tumors detected the mutated allele in approximate 50% of the reads (Fig. 4E) as in edited *Kras* tumors (Fig. 2E). Together, these data suggest that our vector edited one allele of the target gene. Alternatively, homozygous editing could have occurred in these tumor cells, but a remarkable population of nonedited stromal cells infiltrated the tumors and thus lowered the reads of the edited alleles; however, tumor histology did not detect extensive stromal infiltration (Fig. 3). To confirm heterozygous editing, we used laser capture microdissection to isolate carcinoma cells from three tumors induced by AAV-P. Amplicon deep sequencing of the genomic DNA of these tumor cells also detected the mutated allele in approximate 50% of reads (fig. S1), indicating that our vector primarily caused heterozygous editing of *Pik3ca*, thus mimicking human tumors that generally mutate one allele only of a proto-oncogene (*38*).

CRISPR-Cas9 editing is known to sometime cause off-target editing in cultured cell lines (*39*). However, previous whole-genome sequencing (WGS) studies of rodent embryos from editing projects and rodent tumor models detect none to a small number of off-target edits (*40*–*42*). To confirm no substantial off-targeting in our vector system, we performed WGS of one *Pik3ca*-edited tumor and, for comparison, one mouse normal ear punch. We identified 179,332 indel sites that were not in the normal tissue. Only one of these sites matched with the 20-nucleotide gRNA, and this one site was at the intended CRISPR cutting site of a *Pik3ca* allele [indels are known to occur even with these HDR vectors (*18*)]. None of the rest matched even when up to five mismatches were allowed (table S1 and data files S1 and S2). These data confirm that off-target events are rare in our model system.

Having fully validated our vector system for proto-oncogene editing, we additionally tested whether AAV-P could instigate precancerous cells in the MMTV-*Wnt1* mice to progress to cancer. Although both phosphatidylinositol 3-kinase (PI3K) and Wnt signaling are important players in human cancers, they have not been tested for synergy in mammary tumorigenesis in animal models. We found that AAV-P led to a short tumor latency of only 37 days in mice bi-transgenic for MMTV-*Wnt1* and CAG-*SpCas9-P2A-EGFP*, comparable to the swift tumor latency resulting from Lenti-*PIK3CAH1047R* (Fig. 4C). The tumor formation speed in both cohorts of mice is markedly faster than that in AAV-P– and Lenti-*PIK3CAH1047R–*infected mice without the MMTV-*Wnt1* transgene (Fig. 4B) or in MMTV-*Wnt1* mice without viral injection (*31*, *43*), demonstrating that PI3K and Wnt1 collaborate to promote mammary tumorigenesis.

Both AAV-P– and Lenti-*PIK3CAH1047R–*induced tumors on the *Wnt1*-transgenic background are adenocarcinoma, but there are also remarkable histological differences. The Lenti-*PIK3CAH1047R* group (*n* = 10) exhibits significant focal squamous differentiation. In contrast, the AAV-K group (*n* = 7) shows modest squamous differentiation but features prominent papillary presence, thus appearing better differentiated overall (Fig. 3, G and H). These results suggest that the precancerous state also affects edited *Pik3ca* versus overexpressed *PIK3CAH1047R* in specifying tumor characteristics. Together, these data further demonstrate that our modified vector system is highly efficient for editing proto-oncogene in normal and precancerous cells in vivo.

### Genome-edited proto-oncogenes can activate more consistent cellular signaling changes than lentiviral vector-delivered exogenous oncogenes

**Fig. 5.**
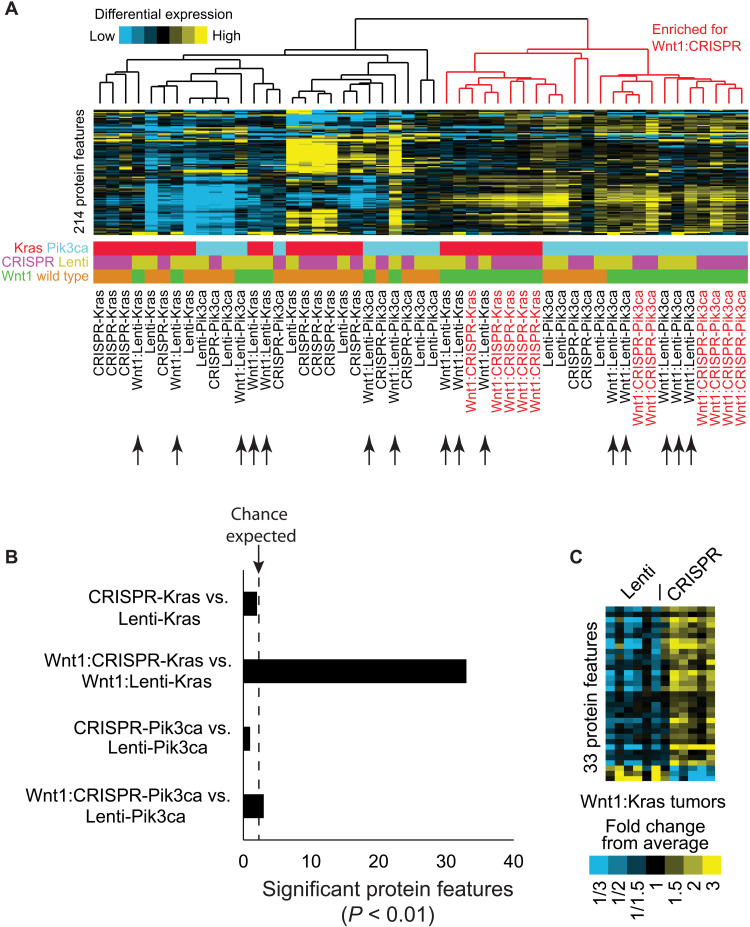
RPPA comparison of tumors generated by proto-oncogene editing and lentivirus-mediated oncogene delivery. (**A**) The unsupervised protein feature clustering of eight groups of mammary tumors. The somatically edited *KrasG12D-* or *Pik3caH1047R*-induced tumors on the MMTV-*Wnt1* background cluster closely (red font labeled), but tumors induced by Lenti-*KrasG12D* or *PIK3CAH1047R* on the MMTV-*Wnt1* background scatter wildly (arrow pointed). (**B**) Bar graph showing differential protein features detected by RPPA (*P* < 0.01 cutoff by *t* test). The dashed line is the expected level of differences caused by random chance due to multiple testing. (**C**) Heatmap showing differential protein features between mammary tumors generated by somatically edited *KrasG12D* and lentivirus-delivered *KrasG12D* on the MMTV-*Wnt1* background.

Virus-delivered mutated genes are inserted into unknown genomic loci and are usually no longer under expression control by their native regulators. Furthermore, their expression levels are also affected by the copy number of provirus and integration sites of the chromatin (positional effects). All these unintended features lead to divergent oncogene expression levels among infected cells and thus to divergent oncogenic signaling and perturbations on signaling networks among different cells (*1*). In contrast, CRISPR-edited genes in all infected cells continue to be under regulation of their native gene loci including promoters, enhancers, splicing machinery, and microRNAs. Consequently, gene editing–initiated tumors may show less intertumoral heterogeneity than tumors induced by lentivirus-delivered oncogenes. To test this possibility, all eight groups of mammary tumors generated in the above experiments were profiled using 214 antibodies in a reverse-phase protein array (RPPA) assay. This panel of antibodies is selected to detect critical signaling pathways important in cancer and development, and 77 of these antibodies detect phosphorylated proteins specifically, therefore directly reporting protein activities (*44*). Unsupervised clustering of the resulting data did not detect any clusters exclusive to any one of the eight groups of tumors, not totally unexpected considering that these tumors all exhibited moderate to high intertumoral heterogeneity and that RPPA surveys only a small subset of proteins produced by tumor cells. In the absence of MMTV-*Wnt1*, both lentiviral and genome-editing models—and regardless of *KrasG12D* or *PIK3CAH1047R* as the initiating oncogene—failed to group even moderately, perhaps due to relatively high intertumoral heterogeneity in each of these four models. On the MMTV-*Wnt1* transgenic background, Lenti-*KrasG12D–*induced tumors and Lenti-*PIK3CAH1047R–*induced tumors also spread through multiple clusters. However, tumors caused by edited *KrasG12D* and *Pik3caH1047R* on the MMTV-*Wnt1* background both clustered closely together (Fig. 5A and table S3), indicating less intertumoral heterogeneity of protein profiles among tumors induced by gene editing than by Lenti-delivered oncogene. These data also suggest that genome-edited proto-oncogenes can result in more consistent perturbations of intracellular signaling networks than lentivirus-delivered oncogenes, at least in some cases. Notably, the observation of close clustering on the MMTV-*Wnt1* precancerous background, but not on the wild-type (WT) background, may be due to a faster course of tumorigenesis in the former cohort, negating sporadic secondary genetic or epigenetic events and their uneven impact on protein profiles that might have occurred in the latter cohort.

To further examine the intertumoral heterogeneity among the tumors generated by these two methods, we performed RNA sequencing (RNA-seq) on four groups of tumors from our *PI3KCAH1047R* experiments. Unsupervised clustering across all samples showed that only the edited *Pik3caH1047R* tumors on the MMTV-*Wnt1* background formed an exclusive cluster (fig. S2). These data again suggest that gene editing leads to more uniform tumors, at least on the *Wnt1* background.

### Genome-edited proto-oncogenes can cause different intracellular signaling compared to lentiviral vector-delivered oncogenes

As discussed above and partly demonstrated by RPPA and RNA-seq, gene editing–initiated tumors likely show less intertumoral heterogeneity than tumors induced by Lenti-delivered oncogenes; therefore, there may be detectable differences in proteins and signaling pathways between tumors initiated by lentivirus-delivered oncogenes versus edited genes. To test this possibility, Student’s *t* test was used to compare these four pairs of tumors. AAV-P–induced tumors in both the absence and presence of the MMTV-*Wnt1* transgene did not differ significantly from corresponding Lenti-*PIK3CAH1047R*–induced tumors in both absence and presence of the MMTV-*Wnt1* transgene (Fig. 5B and fig. S3). AAV-K–induced tumors in mice without the MMTV-*Wnt1* transgene also did not differ from Lenti-*KrasG12D*–induced tumors by more than random chances (Fig. 5B and fig. S3). However, the detected difference of protein features between tumors generated by AAV-K versus Lenti-*KrasG12D* on the MMTV-*Wnt1* background is significantly higher than random chances (Fig. 5, B and C, and fig. S3). Whether these protein differences are due to distinct signaling potencies of edited *Kras* in its native locus versus overexpressed *KrasG12D* or other reasons remains to be determined. However, among the top 10 differentially expressed proteins between the two groups of tumors, five [SOX9, SLUG, GATA3, PIAS1, and signal transducer and activator of transcription 1 (STAT1)] are prominent proteins controlling cell differentiation, consistent with the two groups of tumors showing differences in adenosquamous differentiation (Fig. 3, C and D). Together, these data demonstrate that while the oncogenic drivers generated by these two methods can cause similar cellular signaling pathway changes in some cases, they can also cause significantly different signaling pathway changes in some other cases, which can affect tumor characteristics.

### Genome editing models reveal signaling network alterations caused by a *Wnt1* transgene better than Lenti-oncogene overexpression models

**Fig. 6.**
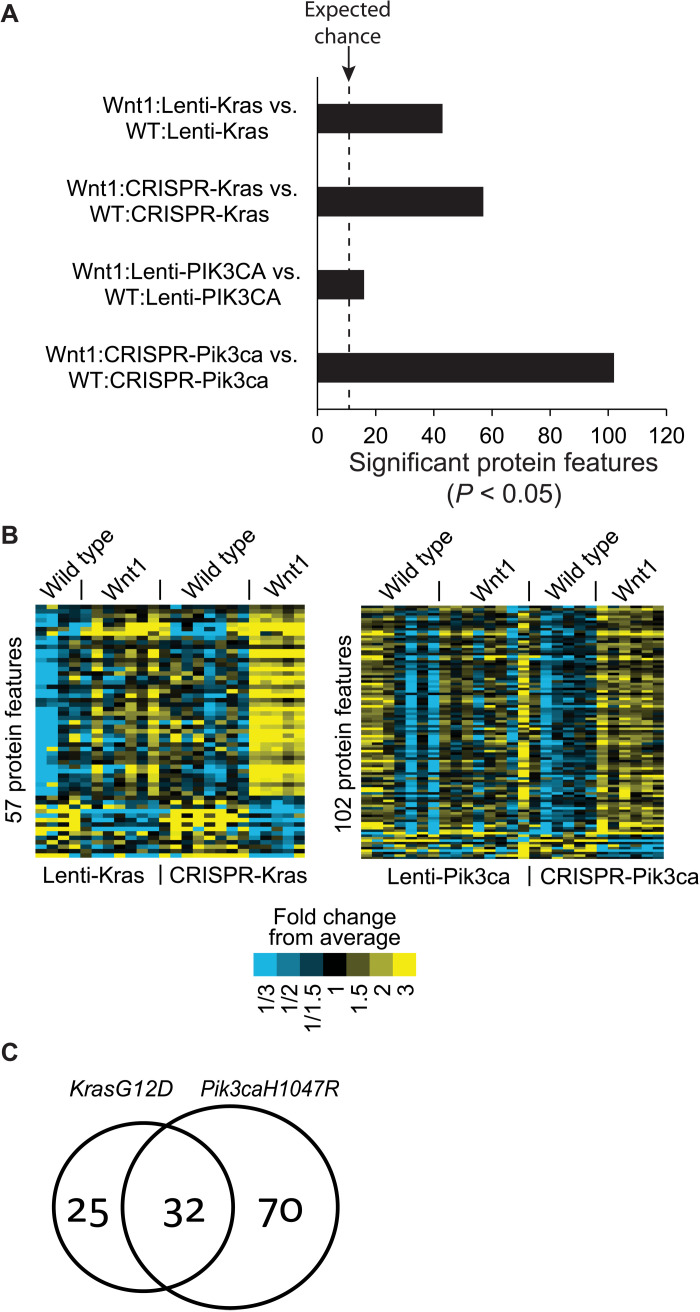
The somatic proto-oncogene editing method can better reveal Wnt signaling protein features than the lentivirus-mediated oncogene delivery method. (**A**) Bar graph showing differential RPPA protein features detected by RPPA (*P* < 0.05 cutoff by *t* test). The dashed line is the expected level of differences caused by random chance due to multiple testing. (**B**) Heatmap showing RPPA protein features. (**C**) Venn diagram showing differential protein features from comparing tumors on the MMTV*-Wnt1* versus WT background. The edited genetic drivers are indicated at the top.

Reduced intertumoral heterogeneity among CRISPR tumors on the transgenic *Wnt1* background suggests that these genome-edited models may also better demonstrate Wnt signaling activity than Lenti-oncogene overexpression models. To test this possibility, we performed Hallmark, Kyoto Encyclopedia of Genes and Genomes, and Wiki pathway analyses using gene set enrichment analysis (GSEA). Wnt signaling pathways were detected only in the list of genes differentially expressed in comparing edited *Pik3ca* tumors on the MMTV-*Wnt1* versus FVB/N background, but not among the genes differentially expressed in comparing Lenti-*PIK3CAH1047R* tumors on the MMTV-*Wnt1* versus FVB/N background (table S2 and data file S4). These data suggest that genome-edited method may reveal the Wnt signaling pathway better than the Lenti method. Next, we explored our RPPA dataset of eight groups of tumors to test whether the gene-editing method is also superior to the Lenti method in detecting the transgenic *Wnt1* impact on Wnt signal transduction components and other protein networks. Forty-three protein features were detected in comparing tumors induced by Lenti-*KrasG12D* on the FVB/N background versus on the MMTV-*Wnt1* background (Fig. 6A and fig. S3). Moderately, more protein features (57) were found in comparing tumors induced by CRISPR-edited *KrasG12D* on the FVB/N background versus on the MMTV-*Wnt1* background (Fig. 6, A and B, and fig. S3). On the other hand, while only 16 protein features were detected in comparing tumors induced by Lenti-*PIK3CAH1047R* on the FVB/N background versus on the MMTV-*Wnt1* background, 102 protein features were different between tumors induced by AAV-P on the FVB/N background versus on the MMTV-*Wnt1* background (Fig. 6, A and B, and fig. S3). This observation of higher differences of protein features in edited oncogene-induced tumors, especially in edited PIK3CA-induced tumors, suggests that reduced intertumoral heterogeneity in these genome-edited models allowed improved detection of Wnt signaling impacts on signaling networks. Thirty-two protein features are shared between these two comparisons of the edited oncogene-induced tumors (Fig. 6C and table S3). Among them are multiple known components of Wnt signaling—Wnt5, β-catenin, c-Myc, SOX9 (*45*, *46*), CtBP (*47*, *48*), nuclear factor κB (*49*), CP (*50*), FOXO1 (*51*), and STAT3 (*52*)—suggesting that the editing method may better reveal protein components of Wnt signaling than the Lenti method. Other proteins in this list likely represent new members of Wnt signaling and proteins that interact with Wnt signaling. Together, these data provide a potential new protein signature of Wnt signaling in tumors and highlight another strength of this in vivo somatic precision gene editing method over the lentivirus-based oncogene delivery method in cancer modeling.

## DISCUSSION

We have successfully edited, without any restrictions, two common proto-oncogenes in somatic mammary epithelial cells which induced tumors with high efficiency and accuracy. Our efficient modeling may be attributed to a few improvements including the disengagement from somatic expression of *Cas9*, *Cre*, and *Luciferase*, which are known to be immunogenic in mice (*25*, *26*), and the destruction of a promoter upstream of the HDR donor, thus preventing the RNA polymerase complex from running into this key functionally important domain and interfering with its function in homologous recombination-based DNA repair. However, the exact mechanism of our high efficiency in precision editing remains to be determined.

Most sporadic tumors evolve through a multistep tumor evolution from single mutated cells in otherwise completely normal tissues. Virus-mediated oncogene delivery and somatic gene editing are superior to conventional GEMMs in allowing both spatial and temporal controls (*1*). AAV-mediated somatic gene editing improves upon retroviral or lentiviral vector-mediated oncogene delivery methods in that no viral vectors are integrated into the genome and that the mutated genes are in their natural loci and completely under the control of their native gene regulators (*1*). Therefore, multiple concerns from using retroviral or lentiviral vectors, including concerns on nonphysiological oncogene expression controls and the integration site effects, can be eliminated. For these reasons, these precision gene editing models better mimic human cancer formation and may provide better models for study of human cancer biology, especially for testing mutations from patients and for preclinical testing of therapeutics for prevention and treatment. Furthermore, as partly demonstrated in Fig. 5, tumor models generated by edited genes likely exhibit less intertumoral heterogeneity within a model than tumor models generated by lentiviral/retroviral-mediated oncogene expression. This feature adds bonus value to these models in preclinical testing of chemopreventive and chemotherapeutic drugs since drug effects may be detected even in smaller numbers of animals.

While we demonstrated this efficient tumor modeling through gene editing in mammary glands, this improved vector system should be applicable in other epithelial tissues or nonepithelial tissues. Depending on the specific tissue to be infected, other AAV serotypes may need to be used, and viral injection methods may need to be optimized since certain tissues (such as prostate and pancreas) are not readily accessible to viral injection. Ex vivo infection of isolated primary cells followed by transplantation could also be a choice when specific tissues or cells are difficult to access. Besides mice, this method should also be adaptable to rats and other laboratory animals, and we have successfully used AAV vectors to introduce indels in tumors suppressor genes in rats to model mammary tumors (*1*).

The epithelia of breast and many other epithelial tissues are composed of stem cells, distinct progenitor cells, and different lineages of differentiated cells. These different cell populations have been found to contribute to cancer differentially, affecting oncogene sensitivity, tumor histopathology, expression profiles, metastasis, and other cancer characteristics (*1*). After injection into the ductal lumen, these AAV vectors likely infect any epithelial cells that they encounter, but the resulting tumors likely arise from a much smaller subset of the infected cell population that evolved into cancer at the fastest speed. This smaller subset could be a specific cell subtype or multiple cell subtypes with similar susceptibilities to transformation. To adapt this system for editing specific cell subsets, multiple strategies can be used: (i) The virus can be pseudotyped for infecting selected cells that are engineered to carry a cognate viral receptor; (ii) a cell type–specific gene promoter can be used to express *Cas9* in the cell type of interest for gene editing; and (iii) selected cell subsets could be purified by flow cytometry and other means, infected by regular AAV vectors ex vivo, and then transplanted back into mice.

Both PI3K and Wnt signaling play critical roles in breast cancer and other human tumors. We have previously reported that *Hras* is mutated and activated in approximately 50% of tumors arising in MMTV-*Wnt1* mice (*31*), and we have previously confirmed that forced expression of *HrasQ61L*, *KrasG12D*, or even *Nras* WT in MMTV-*Wnt1* mice led to rapid tumor appearance (*19*, *53*). Our finding that somatic expression of *PIK3CAH1047R*—by a lentivector or via genome editing—led to swift tumor formation in MMTV-*Wnt1* mice (Fig. 4) suggests that PIK3CA activation collaborates with Wnt1 signaling in mammary tumorigenesis and, as a well-recognized downstream mediator of RAS signaling, can even substitute for RAS in transforming WNT1-activated mammary tumors. By sequencing 56 tumors in MMTV-*Wnt1* mice, we detected one case with a *Pik3ca* hotspot mutation. Of particular interest, on the MMTV-*Wnt1* background, AAV-P–induced tumors (Fig. 3H) exhibited higher similarities to spontaneous tumors arising in noninfected MMTV-*Wnt1* transgenic mice than Lenti-*PIK3CAH1047R*–induced tumors (Fig. 3G). These histological data—together with the finding that tumors induced by AAV-K, but not Lenti-*KrasG12D*, on the MMTV-*Wnt1* background displayed high histological similarities to spontaneous tumors arising in noninfected MMTV-*Wnt1* transgenic mice—provide another evidence that AAV-introduced CRISPR precision editing mimics natural tumor evolution more closely than virus-introduced ectopic expression of oncogenes. Why genome editing and lentivirus-mediated oncogene expression in either normal or precancerous tissues caused histologically different tumors remains to be determined, but difference in oncogene expression levels and the resulting differential impact on signaling networks as revealed in Figs. 5 and 6 likely play a role in driving cell differentiation and cell fate.

In summary, we have improved the CRISPR-Cas9/HDR–mediated gene editing method for installing any missense mutations in somatic cells for tumor modeling in mice. This approach can be used to generate models that better mimic human cancer evolution and response to chemoprevention and treatment. Furthermore, in a proof-of-principle study of two of the most common human proto-oncogenes, we have demonstrated that these improved models of human cancer can uncover some features of cancer evolution that are not observed in tumor models generated by lentivirus-mediated oncogene delivery methods.

## MATERIALS AND METHODS

### Plasmids

The Lenti-*KrasG12D* was constructed by inserting a PCR fragment of *KrasG12D* cDNA into FU-CGW (*54*) at an Eco RI site. Lenti-*PIK2CAH1047R* has been described previously (*33*). The AAV-KPL plasmid (*15*) was obtained from Addgene (plasmid #60224). The *Tp53* gRNA and *Lkb1* gRNA, and part of the EF-1α promoter, were removed from this plasmid by restriction enzymes Age I and Bam HI, to obtain a construct that edits *Kras* int*o KrasG12D* only (AAV-K). Packaging plasmids pAAV2/9n and pAdDeltaF6 were obtained from Addgene (plasmids #112865 and #112867).

To create the construct to edit *Pik3ca* into *Pik3caH1047R*, the *Kras* gRNA and the HDR donor sequence in the AAV-K plasmid were replaced by the *Pik3caH1047R* gRNA (5′-ATGAATGATGCACATCATGG) and the HDR donor sequence for the *Pik3caH1047R* editing, respectively. The gRNA sequence was chosen through running gRNA tool from both PNA Bio (www.pnabio.com) and ATUM (www.atum.bio/eCommerce/cas9/input) by inputting 60 base pairs (bp) of sequence surrounding the *Pik3caH1047R* mutation site. To replace the gRNA in AAV-K, an oligo template was synthesized to contain the sequence matching the region in AAV-K between the enzyme sites Nde I and Bam HI with a substitution of *Pik3ca* gRNA for the *Kras* gRNA. This template was converted into a DNA fragment by PCR using two primers, AAV-349F (5′-CTATCATATGCTTACCGTAAC) and AAV-496R (5′-CCGGGATCCAAAAAAGCACC). The resulting DNA fragment was TA-cloned into the PCR2.1 plasmid. The Nde I and Bam HI fragment from the resulting plasmid was subsequently used to replace the Nde I and Bam HI region in the AAV-K, resulting in an intermediate plasmid (plasmid #1). To replace the *Kras* HDR with *Pik3caH1047R* HDR donor sequence in plasmid #1, a genomic DNA fragment of 813 bp surrounding the *Pik3caH1047* coding region was PCR-cloned into the PCR2.1 vector using the genomic DNA from an FVB/N mouse as template and primers Pik3ca65319F (5′-CCTCCAATGTTCAAGCACTG) and Pik3ca66092R (5′-CAGCATTCTAGTTTTGTCTCC). Into this HDR fragment, the H1047R mutation along with five silent mutations was installed using the Q5 Site-Directed Mutagenesis Kit (New England Biolabs, E0554S) and primers Pik3caMutF (5′-ATGAATGATGCACATCATGGTGGATGGACGACAAAAAT GGATTGGATC) and Pik3caMutR (5′-TTGCTTTGTGAAATATTCCAAAGC). This mutated HDR fragment in PCR2.1 was subsequently used to replace the HDR donor in the intermediate plasmid #1 through restriction enzymes Sac I and Apa I, resulting in the plasmid AAV-P.

### Virus production

Lentivirus production has been described previously (*20*, *24*). AAV9-EF1-copGFP-WPRE-hGH was provided by the Gene Vector Core at Baylor College of Medicine (BCM). Other AAV viruses were produced either by the Gene Vector Core at BCM using density gradient centrifugation or by the authors using the jetPRIME transfection reagent (Polyplus transfection, 114-07) for plasmid transfection, the AAVpro Purification kit (Takara #6675) for virus extraction/purification/concentration, and the AAVpro Titration kit (Takara #6233) for titer determination following the manufacturer’s instructions. Four 100-mm dishes of 293T cells were used for each batch of AAV production. The approximate yield was 100 μl of virus at the titer of 1 × 10^13^ gc/ml.

### Transgenic mice and animal care

FVB/N, MMTV-*Wnt1* mice (*55*) (on the FVB/N background), and Rosa26-Cas9 knock-in on FVB/N [Gt(ROSA)26Sort^m1.1(CAG-cas9*,-EGFP)Fezh^] were purchased from The Jackson Laboratory (Bar Harbor, ME). Male MMTV-*Wnt1* and female Rosa26-Cas9 knock-in mice were bred to generate MMTV-*Wnt1*/Rosa26-Cas9 double transgenic mice. All mice were kept on 2920X Teklad Global Extruded Rodent Diet (Soy Protein-Free; Harlan Laboratories, Indianapolis, IN). Only female mice were used in this study. All procedures using mice were performed in compliance with an Institutional Animal Care and Use Committee–approved animal protocol.

### Intraductal injection

Intraductal injection of virus has been previously described (*5*, *20*).

### Mammary gland fluorescent signal imaging

Mammary glands were collected from euthanized mice and imaged immediately under a fluorescent stereomicroscope (Leica MZ 16 F) through its matched software (LAS 3.8).

### Tissue processing and H&E staining

Tissue processing and hematoxylin and eosin (H&E) staining have been previously described (*5*).

### Preparation of single-cell suspensions from mammary glands and flow cytometry analysis

Preparation of single-cell suspensions from mammary glands and flow cytometry analysis have been described previously (*5*).

### Laser capture microdissection

Formalin-fixed paraffin-embeded (FFPE) tissue blocks were sectioned at 10 μm, mounted on slides covered with polyethylene-naphthalate membrane slide (2 μm) (no. 11505158; Leica, IL, USA), and left to dry overnight at room temperature. Sections were stained with H&E. After 100% alcohol wash, the sections were air-dried and visualized for tumor components. Microdissection was performed using the LMD7 laser microdissection system (Leica) with software Leica Laser Microdissection V 8.2.

### Genomic DNA purification

Genomic DNA from frozen tissue was extracted through proteinase K digestion, phenol chloroform treatment, and DNA precipitation. To extract genomic DNA from laser capture microdissection, the QIAmp DNA Micro kit (Qiagen, 56304) was used following the manufacturer’s protocol. The dissected areas were incubated with 15 μl of lysis buffer and 10 μl of proteinase K at 56°C overnight.

### Detection of edited genomic regions

The tumor DNA was used as a template to generate PCR amplicon at the edited region. The primers used to amplify the region surrounding *KrasG12D* are Kras29976F (5′-CGTCCTTTACAAGCGCACGC) and Kras30099R (5′-GCCTGCTGAAAATGACTGAG). The primers used to amplify the region surrounding *Pik3caH1047R* are Pik3ca65624F (5′-GATGACATTGCATATATCCG) and Pik3ca65741R (5′-GTTCAAAGCATGCTGCTTG). The purified amplicons were sequenced using either Sanger sequencing or Amplicon-EZ Next-Generation sequencing carried out by GENEWIZ (South Plainfield, NJ).

### Whole genome off-target survey through comparing indel sites with the gRNA sequence

The genomic DNA was extracted from an ear punch of a normal mouse (N01) and a tumor (T01) induced by AAV-P and was sequenced by Novogene (Sacramento, CA). The indel calling list of the T01 genome was compared against that of the N01 genome so as to filter out the overlapping indels. The resulting T01-unique indels were analyzed using Tandem Repeats Finder (v4.09.1) to additionally exclude indels at genome locations showing three or more tandem repeats in either the mouse reference genome sequence (GRCm38/mm10) or the T01 sequence (*40*). The remaining 179,332 genomic sites of indels were compared with the list of all potential CRISPR-Cas9 cutting sites in the reference mouse genome. This list was generated using PWMScan (https://ccg.epfl.ch/pwmtools/pwmscan.php) to scan GRCm38/mm10, both forward and reverse strands, for the Pik3ca gRNA allowing up to five mismatches. The 3′ 3-nucleotides of the matched sequences were extracted by Samtools (V1.15.1) for matching with the PAM sequences (NGG).

### RPPA analysis

RPPA assays were carried out as described previously (*44*). Taking the normalized data, technical replicates were averaged together. Log_2_-transformed values were compared between groups using two-sided *t* test. The chance expected number of differential protein features due to multiple testing was estimated by the Storey and Tibshirani method (*56*). Unsupervised hierarchical clustering was carried out using Cluster 3.0 (*57*), with log_2_-transformed protein expression values being centered on the sample median. Visualization of differential patterns using heatmaps was carried out using Java Treeview (*58*).

### RNA sequencing

To purify RNA, the frozen tumors were first crushed using a cryoPREP Dry Pulverizer (Covaris, MA) following the manufacturer’s protocol. The total RNA from each crushed tumor was then purified using a Direct-zol RNA Microprep kit (Zymo Research, Cat. No. R2062) following the manufacturer’s protocol. The purified RNA was then sequenced by Novogene (Sacramento, CA).

### Unsupervised gene transcript clustering

The RNA-seq data of four groups of mammary tumors generated by somatically edited *Pik3caH1047R* and lentivirus-delivered *PIK3CAH1047R* in mice without or with MMTV-*Wnt1* background were compared using unsupervised hierarchical clustering. Only top 2000 most variable gene transcript features across all samples in the dataset were included for the comparison.

### Gene set enrichment analysis

The GSEA was carried out using the previously reported method (*59*).

### Statistical analysis

Each value reported represents the means ± SDs of at least three biological replicates. Student’s *t* test (if normally distributed) was used to test the significance of difference between two means. Kaplan-Meier plots were generated by GraphPad software, which uses the log-rank (Mantel-Cox) test. *P* values were two-sided unless otherwise specified.

## Notes

View/request a protocol for this paper from *Bio-protocol*.

## References

[1] W. Bu, Y. Li, Advances in immunocompetent mouse and rat models, in *Cold Spring Harbor Perspectives Breast Cancer: From Fundamental Biology to Therapeutic Strategies,* in press (2023). 10.1101/cshperspect.a041328 PMC10810718 37217281

[2] Z. Du, K. Podsypanina, S. Huang, A. M. Grath, M. J. Toneff, E. Bogoslovskaia, X. Zhang, R. C. Moraes, M. Fluck, D. C. Allred, M. T. Lewis, H. E. Varmus, Y. Li, Introduction of oncogenes into mammary glands in vivo with an avian retroviral vector initiates and promotes carcinogenesis in mouse models. Proc. Natl. Acad. Sci. U.S.A. 103, 17396–17401 (2006). 17090666 10.1073/pnas.0608607103 PMC1635021

[3] B. C. Wang, W. S. Kennan, J. Yasukawa-Barnes, M. J. Lindstrom, M. N. Gould, Carcinoma induction following direct in situ transfer of v-Ha-ras into rat mammary epithelial cells using replication-defective retrovirus vectors. Cancer Res. 51, 2642–2648 (1991). 2021942

[4] E. C. Holland, H. E. Varmus, Basic fibroblast growth factor induces cell migration and proliferation after glia-specific gene transfer in mice. Proc. Natl. Acad. Sci. U.S.A. 95, 1218–1223 (1998). 9448312 10.1073/pnas.95.3.1218 PMC18724

[5] W. Bu, J. Chen, G. D. Morrison, S. Huang, C. J. Creighton, J. Huang, G. C. Chamness, S. G. Hilsenbeck, D. R. Roop, A. D. Leavitt, Y. Li, Keratin 6a marks mammary bipotential progenitor cells that can give rise to a unique tumor model resembling human normal-like breast cancer. Oncogene 30, 4399–4409 (2011). 21532625 10.1038/onc.2011.147 PMC3156856

[6] A. V. Anzalone, L. W. Koblan, D. R. Liu, Genome editing with CRISPR-Cas nucleases, base editors, transposases and prime editors. Nat. Biotechnol. 38, 824–844 (2020). 32572269 10.1038/s41587-020-0561-9

[7] T. Kaltenbacher, J. Löprich, R. Maresch, J. Weber, S. Müller, R. Oellinger, N. Groß, J. Griger, N. de Andrade Krätzig, P. Avramopoulos, D. Ramanujam, S. Brummer, S. A. Widholz, S. Bärthel, C. Falcomatà, A. Pfaus, A. Alnatsha, J. Mayerle, M. Schmidt-Supprian, M. Reichert, G. Schneider, U. Ehmer, C. J. Braun, D. Saur, S. Engelhardt, R. Rad, CRISPR somatic genome engineering and cancer modeling in the mouse pancreas and liver. Nat. Protoc. 17, 1142–1188 (2022). 35288718 10.1038/s41596-021-00677-0

[8] A. Katti, B. J. Diaz, C. M. Caragine, N. E. Sanjana, L. E. Dow, CRISPR in cancer biology and therapy. Nat. Rev. Cancer 22, 259–279 (2022). 35194172 10.1038/s41568-022-00441-w

[9] S. Nik-Zainal, H. Davies, J. Staaf, M. Ramakrishna, D. Glodzik, X. Zou, I. Martincorena, L. B. Alexandrov, S. Martin, D. C. Wedge, P. van Loo, Y. S. Ju, M. Smid, A. B. Brinkman, S. Morganella, M. R. Aure, O. C. Lingjærde, A. Langerød, M. Ringnér, S. M. Ahn, S. Boyault, J. E. Brock, A. Broeks, A. Butler, C. Desmedt, L. Dirix, S. Dronov, A. Fatima, J. A. Foekens, M. Gerstung, G. K. J. Hooijer, S. J. Jang, D. R. Jones, H. Y. Kim, T. A. King, S. Krishnamurthy, H. J. Lee, J. Y. Lee, Y. Li, S. McLaren, A. Menzies, V. Mustonen, S. O’Meara, I. Pauporté, X. Pivot, C. A. Purdie, K. Raine, K. Ramakrishnan, F. G. Rodríguez-González, G. Romieu, A. M. Sieuwerts, P. T. Simpson, R. Shepherd, L. Stebbings, O. A. Stefansson, J. Teague, S. Tommasi, I. Treilleux, G. G. van den Eynden, P. Vermeulen, A. Vincent-Salomon, L. Yates, C. Caldas, L. v.’t. Veer, A. Tutt, S. Knappskog, B. K. T. Tan, J. Jonkers, Å. Borg, N. T. Ueno, C. Sotiriou, A. Viari, P. A. Futreal, P. J. Campbell, P. N. Span, S. van Laere, S. R. Lakhani, J. E. Eyfjord, A. M. Thompson, E. Birney, H. G. Stunnenberg, M. J. van de Vijver, J. W. M. Martens, A. L. Børresen-Dale, A. L. Richardson, G. Kong, G. Thomas, M. R. Stratton, Landscape of somatic mutations in 560 breast cancer whole-genome sequences. Nature 534, 47–54 (2016). 27135926 10.1038/nature17676 PMC4910866

[10] I. C. Kurt, R. Zhou, S. Iyer, S. P. Garcia, B. R. Miller, L. M. Langner, J. Grünewald, J. K. Joung, CRISPR C-to-G base editors for inducing targeted DNA transversions in human cells. Nat. Biotechnol. 39, 41–46 (2021). 32690971 10.1038/s41587-020-0609-x PMC7854778

[11] T. Yuan, N. Yan, T. Fei, J. Zheng, J. Meng, N. Li, J. Liu, H. Zhang, L. Xie, W. Ying, D. Li, L. Shi, Y. Sun, Y. Li, Y. Li, Y. Sun, E. Zuo, Optimization of C-to-G base editors with sequence context preference predictable by machine learning methods. Nat. Commun. 12, 4902 (2021). 34385461 10.1038/s41467-021-25217-y PMC8361092

[12] S. Annunziato, C. Lutz, L. Henneman, J. Bhin, K. Wong, B. Siteur, B. van Gerwen, R. de Korte-Grimmerink, M. P. Zafra, E. M. Schatoff, A. P. Drenth, E. van der Burg, T. Eijkman, S. Mukherjee, K. Boroviak, L. F. Wessels, M. van de Ven, I. J. Huijbers, D. J. Adams, L. E. Dow, J. Jonkers, In situ CRISPR-Cas9 base editing for the development of genetically engineered mouse models of breast cancer. EMBO J. 39, e102169 (2020). 31930530 10.15252/embj.2019102169 PMC7049816

[13] M. P. Zafra, E. M. Schatoff, A. Katti, M. Foronda, M. Breinig, A. Y. Schweitzer, A. Simon, T. Han, S. Goswami, E. Montgomery, J. Thibado, E. R. Kastenhuber, F. J. Sánchez-Rivera, J. Shi, C. R. Vakoc, S. W. Lowe, D. F. Tschaharganeh, L. E. Dow, Optimized base editors enable efficient editing in cells, organoids and mice. Nat. Biotechnol. 36, 888–893 (2018). 29969439 10.1038/nbt.4194 PMC6130889

[14] P. Liu, S. Q. Liang, C. Zheng, E. Mintzer, Y. G. Zhao, K. Ponnienselvan, A. Mir, E. J. Sontheimer, G. Gao, T. R. Flotte, S. A. Wolfe, W. Xue, Improved prime editors enable pathogenic allele correction and cancer modelling in adult mice. Nat. Commun. 12, 2121 (2021). 33837189 10.1038/s41467-021-22295-w PMC8035190

[15] R. J. Platt, S. Chen, Y. Zhou, M. J. Yim, L. Swiech, H. R. Kempton, J. E. Dahlman, O. Parnas, T. M. Eisenhaure, M. Jovanovic, D. B. Graham, S. Jhunjhunwala, M. Heidenreich, R. J. Xavier, R. Langer, D. G. Anderson, N. Hacohen, A. Regev, G. Feng, P. A. Sharp, F. Zhang, CRISPR-Cas9 knockin mice for genome editing and cancer modeling. Cell 159, 440–455 (2014). 25263330 10.1016/j.cell.2014.09.014 PMC4265475

[16] B. Oldrini, Á. Curiel-García, C. Marques, V. Matia, Ö. Uluçkan, O. Graña-Castro, R. Torres-Ruiz, S. Rodriguez-Perales, J. T. Huse, M. Squatrito, Somatic genome editing with the RCAS-TVA-CRISPR-Cas9 system for precision tumor modeling. Nat. Commun. 9, 1466 (2018). 29654229 10.1038/s41467-018-03731-w PMC5899147

[17] H. Ji, M. R. Ramsey, D. N. Hayes, C. Fan, K. McNamara, P. Kozlowski, C. Torrice, M. C. Wu, T. Shimamura, S. A. Perera, M. C. Liang, D. Cai, G. N. Naumov, L. Bao, C. M. Contreras, D. Li, L. Chen, J. Krishnamurthy, J. Koivunen, L. R. Chirieac, R. F. Padera, R. T. Bronson, N. I. Lindeman, D. C. Christiani, X. Lin, G. I. Shapiro, P. A. Jänne, B. E. Johnson, M. Meyerson, D. J. Kwiatkowski, D. H. Castrillon, N. Bardeesy, N. E. Sharpless, K. K. Wong, LKB1 modulates lung cancer differentiation and metastasis. Nature 448, 807–810 (2007). 17676035 10.1038/nature06030

[18] I. P. Winters, S. H. Chiou, N. K. Paulk, C. D. McFarland, P. V. Lalgudi, R. K. Ma, L. Lisowski, A. J. Connolly, D. A. Petrov, M. A. Kay, M. M. Winslow, Multiplexed in vivo homology-directed repair and tumor barcoding enables parallel quantification of Kras variant oncogenicity. Nat. Commun. 8, 2053 (2017). 29233960 10.1038/s41467-017-01519-y PMC5727199

[19] W. Bu, Z. Liu, W. Jiang, C. Nagi, S. Huang, D. P. Edwards, E. Jo, Q. Mo, C. J. Creighton, S. G. Hilsenbeck, A. D. Leavitt, M. T. Lewis, S. T. C. Wong, Y. Li, Mammary precancerous stem and non-stem cells evolve into cancers of distinct subtypes. Cancer Res. 79, 61–71 (2019). 30401712 10.1158/0008-5472.CAN-18-1087 PMC6318055

[20] W. Bu, L. Xin, M. Toneff, L. Li, Y. Li, Lentivirus vectors for stably introducing genes into mammary epithelial cells in vivo. J. Mammary Gland Biol. Neoplasia 14, 401–404 (2009). 19936990 10.1007/s10911-009-9154-4

[21] A. N. Johnston, W. Bu, S. Hein, S. Garcia, L. Camacho, L. Xue, L. Qin, C. Nagi, S. G. Hilsenbeck, J. Kapali, K. Podsypanina, J. Nangia, Y. Li, Hyperprolactinemia-inducing antipsychotics increase breast cancer risk by activating JAK-STAT5 in precancerous lesions. Breast cancer research : BCR 20, 42 (2018). 29778097 10.1186/s13058-018-0969-z PMC5960176

[22] S. Wagner, R. Thresher, R. Bland, G. Laible, Adeno-associated-virus-mediated transduction of the mammary gland enables sustained production of recombinant proteins in milk. Sci. Rep. 5, 15115 (2015). 26463440 10.1038/srep15115 PMC4604487

[23] A. Zehir, R. Benayed, R. H. Shah, A. Syed, S. Middha, H. R. Kim, P. Srinivasan, J. Gao, D. Chakravarty, S. M. Devlin, M. D. Hellmann, D. A. Barron, A. M. Schram, M. Hameed, S. Dogan, D. S. Ross, J. F. Hechtman, D. F. DeLair, J. J. Yao, D. L. Mandelker, D. T. Cheng, R. Chandramohan, A. S. Mohanty, R. N. Ptashkin, G. Jayakumaran, M. Prasad, M. H. Syed, A. B. Rema, Z. Y. Liu, K. Nafa, L. Borsu, J. Sadowska, J. Casanova, R. Bacares, I. J. Kiecka, A. Razumova, J. B. Son, L. Stewart, T. Baldi, K. A. Mullaney, H. al-Ahmadie, E. Vakiani, A. A. Abeshouse, A. V. Penson, P. Jonsson, N. Camacho, M. T. Chang, H. H. Won, B. E. Gross, R. Kundra, Z. J. Heins, H. W. Chen, S. Phillips, H. Zhang, J. Wang, A. Ochoa, J. Wills, M. Eubank, S. B. Thomas, S. M. Gardos, D. N. Reales, J. Galle, R. Durany, R. Cambria, W. Abida, A. Cercek, D. R. Feldman, M. M. Gounder, A. A. Hakimi, J. J. Harding, G. Iyer, Y. Y. Janjigian, E. J. Jordan, C. M. Kelly, M. A. Lowery, L. G. T. Morris, A. M. Omuro, N. Raj, P. Razavi, A. N. Shoushtari, N. Shukla, T. E. Soumerai, A. M. Varghese, R. Yaeger, J. Coleman, B. Bochner, G. J. Riely, L. B. Saltz, H. I. Scher, P. J. Sabbatini, M. E. Robson, D. S. Klimstra, B. S. Taylor, J. Baselga, N. Schultz, D. M. Hyman, M. E. Arcila, D. B. Solit, M. Ladanyi, M. F. Berger, Mutational landscape of metastatic cancer revealed from prospective clinical sequencing of 10,000 patients. Nat. Med. 23, 703–713 (2017). 28481359 10.1038/nm.4333 PMC5461196

[24] W. Bu, Y. Li, Intraductal injection of lentivirus vectors for stably introducing genes into rat mammary epithelial cells in vivo. J. Mammary Gland Biol. Neoplasia 25, 389–396 (2020). 33165800 10.1007/s10911-020-09469-w PMC7965254

[25] S. Annunziato, S. M. Kas, M. Nethe, H. Yücel, J. del Bravo, C. Pritchard, R. Bin Ali, B. van Gerwen, B. Siteur, A. P. Drenth, E. Schut, M. van de Ven, M. C. Boelens, S. Klarenbeek, I. J. Huijbers, M. H. van Miltenburg, J. Jonkers, Modeling invasive lobular breast carcinoma by CRISPR/Cas9-mediated somatic genome editing of the mammary gland. Genes Dev. 30, 1470–1480 (2016). 27340177 10.1101/gad.279190.116 PMC4926868

[26] D. Wang, H. Mou, S. Li, Y. Li, S. Hough, K. Tran, J. Li, H. Yin, D. G. Anderson, E. J. Sontheimer, Z. Weng, G. Gao, W. Xue, Adenovirus-mediated somatic genome editing of Pten by CRISPR/Cas9 in mouse liver in spite of Cas9-specific immune responses. Hum. Gene Ther. 26, 432–442 (2015). 26086867 10.1089/hum.2015.087 PMC4509492

[27] M. DuPage, A. F. Cheung, C. Mazumdar, M. M. Winslow, R. Bronson, L. M. Schmidt, D. Crowley, J. Chen, T. Jacks, Endogenous T cell responses to antigens expressed in lung adenocarcinomas delay malignant tumor progression. Cancer Cell 19, 72–85 (2011). 21251614 10.1016/j.ccr.2010.11.011 PMC3069809

[28] S. P. Petkov, F. Heuts, O. A. Krotova, A. Kilpelainen, G. Engström, E. S. Starodubova, M. G. Isaguliants, Evaluation of immunogen delivery by DNA immunization using non-invasive bioluminescence imaging. Hum. Vaccin. Immunother. 9, 2228–2236 (2013). 23896580 10.4161/hv.25561 PMC3906409

[29] M. Gu, T. Xu, P. Chang, *KRAS/LKB1* and *KRAS/TP53* co-mutations create divergent immune signatures in lung adenocarcinomas. Ther. Adv. Med. Oncol. 13, 17588359211006950 (2021). 33995590 10.1177/17588359211006950 PMC8072935

[30] C. M. D'Cruz, E. J. Gunther, R. B. Boxer, J. L. Hartman, L. Sintasath, S. E. Moody, J. D. Cox, S. I. Ha, G. K. Belka, A. Golant, R. D. Cardiff, L. A. Chodosh, c-MYC induces mammary tumorigenesis by means of a preferred pathway involving spontaneous Kras2 mutations. Nat. Med. 7, 235–239 (2001). 11175856 10.1038/84691

[31] K. Podsypanina, Y. Li, H. E. Varmus, Evolution of somatic mutations in mammary tumors in transgenic mice is influenced by the inherited genotype. BMC Med. 2, 24 (2004). 15198801 10.1186/1741-7015-2-24 PMC446228

[32] Cancer Genome Atlas Network, Comprehensive molecular portraits of human breast tumours. Nature 490, 61–70 (2012). 23000897 10.1038/nature11412 PMC3465532

[33] A. Young, W. Bu, W. Jiang, A. Ku, J. Kapali, S. Dhamne, L. Qin, S. G. Hilsenbeck, Y. C. N. du, Y. Li, Targeting the pro-survival protein BCL-2 to prevent breast cancer. Cancer Prev. Res. 15, 3–10 (2022). 10.1158/1940-6207.CAPR-21-0031 PMC8741732 34667127

[34] P. Liu, H. Cheng, S. Santiago, M. Raeder, F. Zhang, A. Isabella, J. Yang, D. J. Semaan, C. Chen, E. A. Fox, N. S. Gray, J. Monahan, R. Schlegel, R. Beroukhim, G. B. Mills, J. J. Zhao, Oncogenic PIK3CA-driven mammary tumors frequently recur via PI3K pathway-dependent and PI3K pathway-independent mechanisms. Nat. Med. 17, 1116–1120 (2011). 21822287 10.1038/nm.2402 PMC3169724

[35] A. B. Hanker, A. D. Pfefferle, J. M. Balko, M. G. Kuba, C. D. Young, V. Sánchez, C. R. Sutton, H. Cheng, C. M. Perou, J. J. Zhao, R. S. Cook, C. L. Arteaga, Mutant PIK3CA accelerates HER2-driven transgenic mammary tumors and induces resistance to combinations of anti-HER2 therapies. Proc. Natl. Acad. Sci. U.S.A. 110, 14372–14377 (2013). 23940356 10.1073/pnas.1303204110 PMC3761610

[36] S. Koren, L. Reavie, J. P. Couto, D. de Silva, M. B. Stadler, T. Roloff, A. Britschgi, T. Eichlisberger, H. Kohler, O. Aina, R. D. Cardiff, M. Bentires-Alj, PIK3CA(H1047R) induces multipotency and multi-lineage mammary tumours. Nature 525, 114–118 (2015). 26266975 10.1038/nature14669

[37] A. Van Keymeulen, M. Y. Lee, M. Ousset, S. Brohée, S. Rorive, R. R. Giraddi, A. Wuidart, G. Bouvencourt, C. Dubois, I. Salmon, C. Sotiriou, W. A. Phillips, C. Blanpain, Reactivation of multipotency by oncogenic PIK3CA induces breast tumour heterogeneity. Nature 525, 119–123 (2015). 26266985 10.1038/nature14665

[38] C. M. Bielski, M. T. A. Donoghue, M. Gadiya, A. J. Hanrahan, H. H. Won, M. T. Chang, P. Jonsson, A. V. Penson, A. Gorelick, C. Harris, A. M. Schram, A. Syed, A. Zehir, P. B. Chapman, D. M. Hyman, D. B. Solit, K. Shannon, S. Chandarlapaty, M. F. Berger, B. S. Taylor, Widespread selection for oncogenic mutant allele imbalance in cancer. Cancer Cell 34, 852–862.e4 (2018). 30393068 10.1016/j.ccell.2018.10.003 PMC6234065

[39] P. D. Hsu, D. A. Scott, J. A. Weinstein, F. A. Ran, S. Konermann, V. Agarwala, Y. Li, E. J. Fine, X. Wu, O. Shalem, T. J. Cradick, L. A. Marraffini, G. Bao, F. Zhang, DNA targeting specificity of RNA-guided Cas9 nucleases. Nat. Biotechnol. 31, 827–832 (2013). 23873081 10.1038/nbt.2647 PMC3969858

[40] M. Zuckermann, V. Hovestadt, C. B. Knobbe-Thomsen, M. Zapatka, P. A. Northcott, K. Schramm, J. Belic, D. T. W. Jones, B. Tschida, B. Moriarity, D. Largaespada, M. F. Roussel, A. Korshunov, G. Reifenberger, S. M. Pfister, P. Lichter, D. Kawauchi, J. Gronych, Somatic CRISPR/Cas9-mediated tumour suppressor disruption enables versatile brain tumour modelling. Nat. Commun. 6, 7391 (2015). 26067104 10.1038/ncomms8391 PMC4467376

[41] K. R. Anderson, M. Haeussler, C. Watanabe, V. Janakiraman, J. Lund, Z. Modrusan, J. Stinson, Q. Bei, A. Buechler, C. Yu, S. R. Thamminana, L. Tam, M. A. Sowick, T. Alcantar, N. O’Neil, J. Li, L. Ta, L. Lima, M. Roose-Girma, X. Rairdan, S. Durinck, S. Warming, CRISPR off-target analysis in genetically engineered rats and mice. Nat. Methods 15, 512–514 (2018). 29786090 10.1038/s41592-018-0011-5 PMC6558654

[42] V. Iyer, K. Boroviak, M. Thomas, B. Doe, L. Riva, E. Ryder, D. J. Adams, No unexpected CRISPR-Cas9 off-target activity revealed by trio sequencing of gene-edited mice. PLOS Genet. 14, e1007503 (2018). 29985941 10.1371/journal.pgen.1007503 PMC6057650

[43] Y. Li, K. Podsypanina, X. Liu, A. Crane, L. K. Tan, R. Parsons, H. E. Varmus, Deficiency of Pten accelerates mammary oncogenesis in MMTV-Wnt-1 transgenic mice. BMC Mol. Biol. 2, 2 (2001). 11178110 10.1186/1471-2199-2-2 PMC29091

[44] C. Coarfa, S. L. Grimm, K. Rajapakshe, D. Perera, H. Y. Lu, X. Wang, K. R. Christensen, Q. Mo, D. P. Edwards, S. Huang, Reverse-phase protein array: Technology, application, data processing, and integration. J. Biomol. Tech. 32, 15–29 (2021). 34025221 10.7171/jbt.21-3202-001 PMC7861052

[45] P. Blache, M. van de Wetering, I. Duluc, C. Domon, P. Berta, J. N. Freund, H. Clevers, P. Jay, SOX9 is an intestine crypt transcription factor, is regulated by the Wnt pathway, and represses the CDX2 and MUC2 genes. J. Cell Biol. 166, 37–47 (2004). 15240568 10.1083/jcb.200311021 PMC2172132

[46] H. Akiyama, J. P. Lyons, Y. Mori-Akiyama, X. Yang, R. Zhang, Z. Zhang, J. M. Deng, M. M. Taketo, T. Nakamura, R. R. Behringer, P. D. McCrea, B. de Crombrugghe, Interactions between Sox9 and beta-catenin control chondrocyte differentiation. Genes Dev. 18, 1072–1087 (2004). 15132997 10.1101/gad.1171104 PMC406296

[47] F. Hamada, M. Bienz, The APC tumor suppressor binds to C-terminal binding protein to divert nuclear beta-catenin from TCF. Dev. Cell 7, 677–685 (2004). 15525529 10.1016/j.devcel.2004.08.022

[48] M. Fang, J. Li, T. Blauwkamp, C. Bhambhani, N. Campbell, K. M. Cadigan, C-terminal-binding protein directly activates and represses Wnt transcriptional targets in Drosophila. EMBO J. 25, 2735–2745 (2006). 16710294 10.1038/sj.emboj.7601153 PMC1500853

[49] J. Deng, S. A. Miller, H. Y. Wang, W. Xia, Y. Wen, B. P. Zhou, Y. Li, S. Y. Lin, M. C. Hung, beta-catenin interacts with and inhibits NF-kappa B in human colon and breast cancer. Cancer Cell 2, 323–334 (2002). 12398896 10.1016/s1535-6108(02)00154-x

[50] K. I. Takemaru, R. T. Moon, The transcriptional coactivator CBP interacts with beta-catenin to activate gene expression. J. Cell Biol. 149, 249–254 (2000). 10769018 10.1083/jcb.149.2.249 PMC2175158

[51] M. A. Essers, L. M. M. de Vries-Smits, N. Barker, P. E. Polderman, B. M. T. Burgering, H. C. Korswagen, Functional interaction between β-catenin and FOXO in oxidative stress signaling. Science 308, 1181–1184 (2005). 15905404 10.1126/science.1109083

[52] R. Huang, S. Wang, N. Wang, Y. Zheng, J. Zhou, B. Yang, X. Wang, J. Zhang, L. Guo, S. Wang, Z. Chen, Z. Wang, S. Xiang, CCL5 derived from tumor-associated macrophages promotes prostate cancer stem cells and metastasis via activating β-catenin/STAT3 signaling. Cell Death Dis. 11, 234 (2020). 32300100 10.1038/s41419-020-2435-y PMC7162982

[53] Z. Y. Zheng, L. Tian, W. Bu, C. Fan, X. Gao, H. Wang, Y. H. Liao, Y. Li, M. T. Lewis, D. Edwards, T. P. Zwaka, S. G. Hilsenbeck, D. Medina, C. M. Perou, C. J. Creighton, X. H. F. Zhang, E. C. Chang, Wild-type N-Ras, overexpressed in basal-like breast cancer, promotes tumor formation by inducing IL-8 secretion via JAK2 activation. Cell Rep. 12, 511–524 (2015). 26166574 10.1016/j.celrep.2015.06.044 PMC4512851

[54] L. Xin, M. A. Teitell, D. A. Lawson, A. Kwon, I. K. Mellinghoff, O. N. Witte, Progression of prostate cancer by synergy of AKT with genotropic and nongenotropic actions of the androgen receptor. Proc. Natl. Acad. Sci. U.S.A. 103, 7789–7794 (2006). 16682621 10.1073/pnas.0602567103 PMC1458510

[55] A. S. Tsukamoto, R. Grosschedl, R. C. Guzman, T. Parslow, H. E. Varmus, Expression of the int-1 gene in transgenic mice is associated with mammary gland hyperplasia and adenocarcinomas in male and female mice. Cell 55, 619–625 (1988). 3180222 10.1016/0092-8674(88)90220-6

[56] J. D. Storey, R. Tibshirani, Statistical significance for genomewide studies. Proc. Natl. Acad. Sci. U.S.A. 100, 9440–9445 (2003). 12883005 10.1073/pnas.1530509100 PMC170937

[57] M. B. Eisen, P. T. Spellman, P. O. Brown, D. Botstein, Cluster analysis and display of genome-wide expression patterns. Proc. Natl. Acad. Sci. U.S.A. 95, 14863–14868 (1998). 9843981 10.1073/pnas.95.25.14863 PMC24541

[58] A. J. Saldanha, Java Treeview—Extensible visualization of microarray data. Bioinformatics 20, 3246–3248 (2004). 15180930 10.1093/bioinformatics/bth349

[59] A. Subramanian, P. Tamayo, V. K. Mootha, S. Mukherjee, B. L. Ebert, M. A. Gillette, A. Paulovich, S. L. Pomeroy, T. R. Golub, E. S. Lander, J. P. Mesirov, Gene set enrichment analysis: A knowledge-based approach for interpreting genome-wide expression profiles. Proc. Natl. Acad. Sci. U.S.A. 102, 15545–15550 (2005). 16199517 10.1073/pnas.0506580102 PMC1239896

