# Supplementary material for: Efficient cancer modeling through CRISPR-Cas9/HDR-based somatic precision gene editing in mice
Source: Sci Adv. 2023 May 12;9(19):eade0059. doi: 10.1126/sciadv.ade0059 (PMC10181191; doi:10.1126/sciadv.ade0059)
Supplement: Supplementary file 1 — Figs. S1 and S2 Tables S1 to S3 Legends for data files S1 to S4 [file sciadv.ade0059_sm.pdf]

Science Advances
AAAS

Supplementary Materials for

Efficient cancer modeling through CRISPR-Cas9/HDR-based somatic precision gene editing in mice

Wen Bu *et al.*

Corresponding author: Wen Bu, wbu@bcm.edu; Yi Li, liyi@bcm.edu

Sci. Adv. **9**, eade0059 (2023)
DOI: 10.1126/sciadv.ade0059

The PDF file includes:

Figs. S1 and S2
Tables S1 to S3
Legends for data files S1 to S4

Other Supplementary Material for this manuscript includes the following:

Data files S1 to S4

Supplemental fig. S1

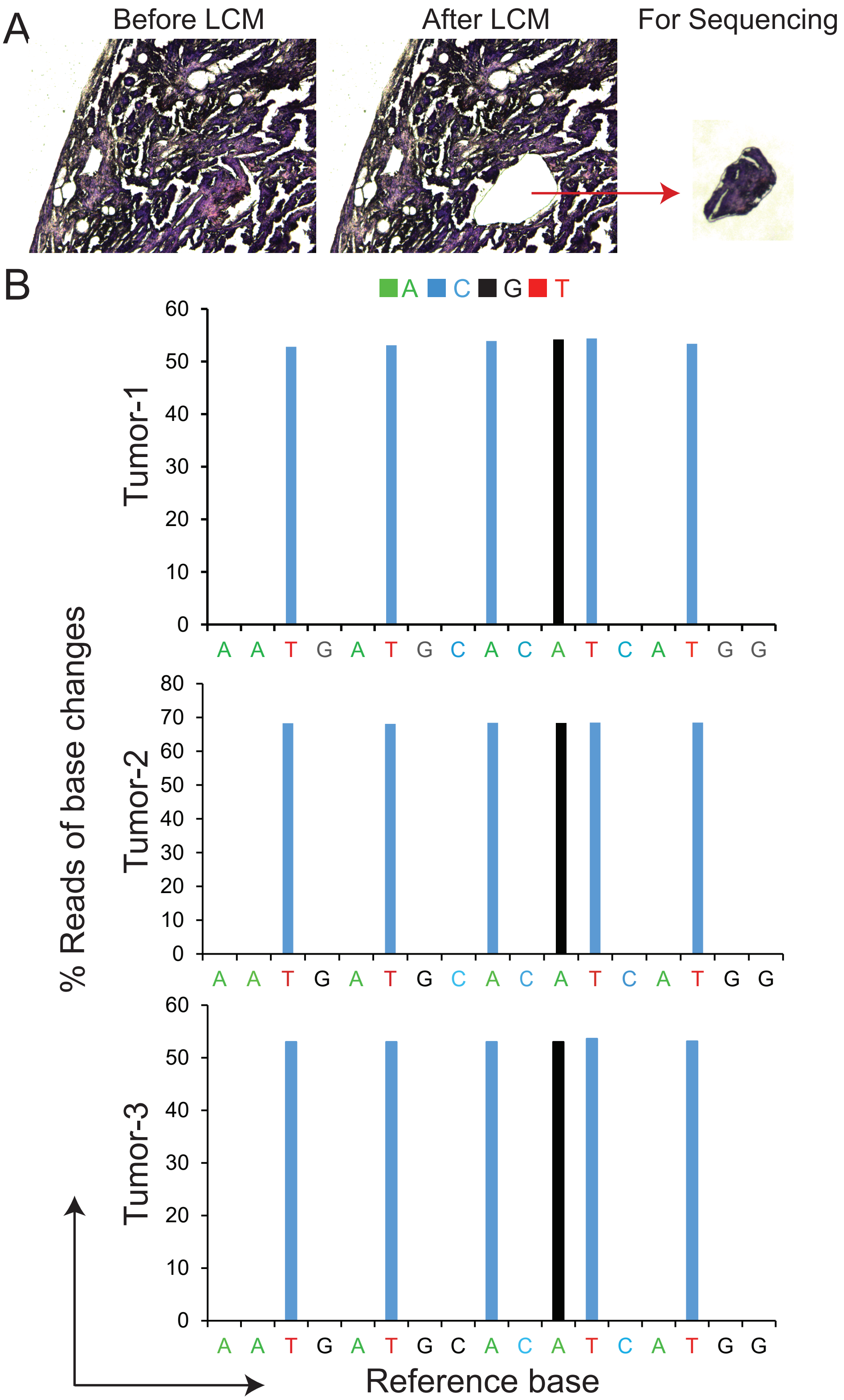

Supplemental fig. S1. Tumor cells from laser capture microdissection (LCM) are majorly heterozygous for the edited gene. (A) A representative LCM tumor sample used for subsequent Amplicon sequencing. (B) The percentages of edited alleles of LCM samples. Three LCM samples from 3 independent tumors induced by somatic editing of *Pik3caH1047R* were sequenced using Amplicon Next-Generation sequencing.

Supplemental fig. S2

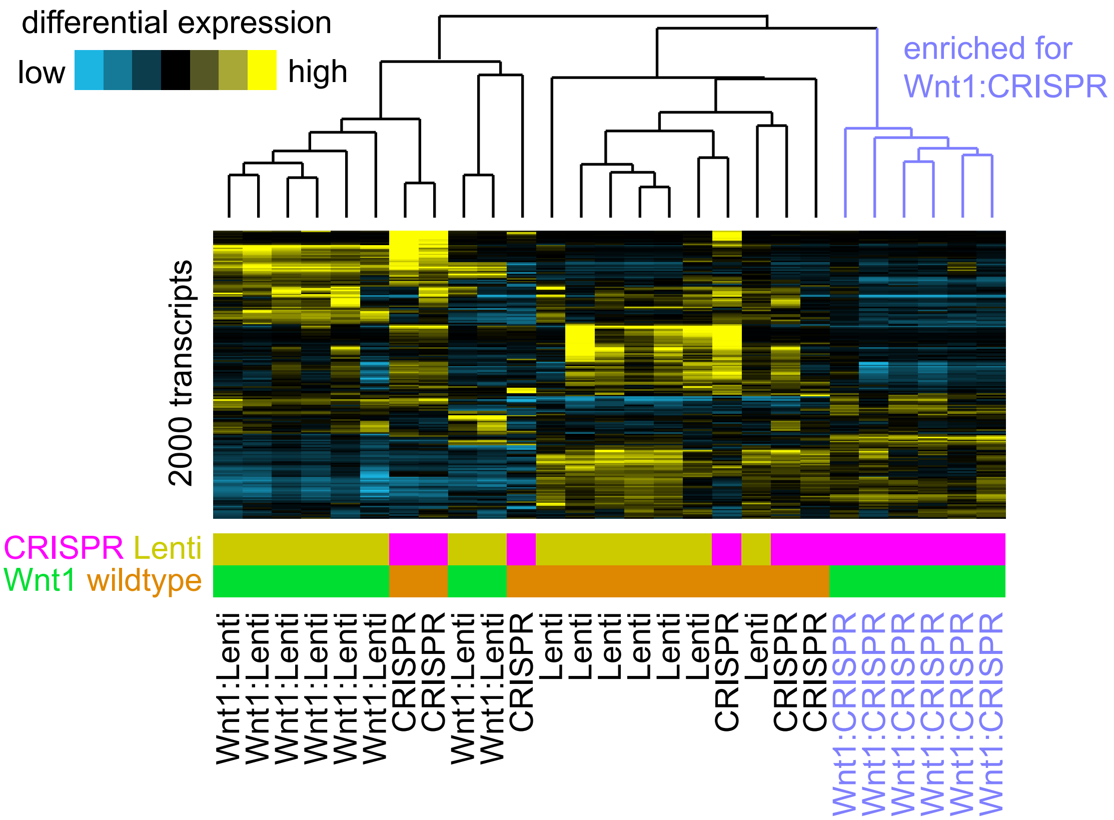

Supplemental fig. S2. The unsupervised gene transcript clustering of 4 groups of Pik3caH1047R mammary tumors. Hierarchical clustering of RNA-seq data were carried out using the top 2000 most variable gene transcript features in the dataset.

Supplemental table S1. Matching gRNA at indel sites

	Forward strand				Reverse strand			
	Without PAM		With PAM		Without PAM		With PAM	
# of unmatched positions	Without indel	With indel	Without indel	With indel	Without indel	With indel	Without indel	With indel
0	0	0	0	1*	0	0	0	0
1	1	0	0	0	0	0	0	0
2	9	0	0	0	4	0	0	0
3	127	0	6	0	127	0	4	0
4	1261	0	74	0	1304	0	70	0
5	10532	4	501	0	9932	0	517	0

* Intended editing site (Pik3ca)

Supplemental table S2. Pathway comparisons of RNA-Seq data of CRISPR-PIK3CA tumors on Wnt1 vs. WT, and Lent-PIK3CAH1047R tumors on Wnt1 vs. WT

		CRISPR	CRISPR	Lenti	Lenti
**PATHWAY NAME**	**SIZE**	**NES**	**NOM p-val**	**NES**	**NOM p-val**
WP_CILIOPATHIES	175	3.196	0.000	-1.893	0.010
WP_BARDETBIEDL_SYNDROME	84	2.993	0.000	-2.485	0.000
HALLMARK_WNT_BETA_CATENIN_SIGNALING	42	2.862	0.000	-1.348	0.131
KEGG_OLFACTORY_TRANSDUCTION	177	2.788	0.000	3.711	0.000
WP_CLEAR_CELL_RENAL_CELL_CARCINOMA_PATHWAYS	84	2.689	0.000	-2.236	0.002
WP_WNTBETACATENIN_SIGNALING_PATHWAY_IN_LEUKEMIA	26	2.560	0.000	-0.868	0.633
WP_GENES_RELATED_TO_PRIMARY_CILIUM_DEVELOPMENT_BASED_ON_CRISPR	100	2.494	0.000	-1.637	0.020
WP_NOTCH_SIGNALING_PATHWAY	61	2.489	0.000	-2.131	0.000
WP_PATHWAYS_AFFECTED_IN_ADENOID_CYSTIC_CARCINOMA	65	2.439	0.000	-1.064	0.355
KEGG_PATHWAYS_IN_CANCER	323	2.391	0.000	-1.709	0.022
WP_PRIMARY_FOCAL_SEGMENTAL_GLOMERULOSCLEROSIS_FSGS	70	2.386	0.002	-3.329	0.000
WP_MALIGNANT_PLEURAL_MESOTHELIOMA	428	2.333	0.000	-3.294	0.000
WP_CIRCADIAN_RHYTHM_GENES	199	2.286	0.000	-0.955	0.503
WP_HEMATOPOIETIC_STEM_CELL_GENE_REGULATION_BY_GABP_ALPHABETA_COMPLEX	20	2.149	0.004	0.777	0.718
WP_NOTCH_SIGNALING	45	2.147	0.000	-1.547	0.064
KEGG_CIRCADIAN_RHYTHM_MAMMAL	13	2.109	0.000	-0.774	0.763
WP_NEURAL_CREST_DIFFERENTIATION	100	2.109	0.000	-1.372	0.111
WP_GLIOBLASTOMA_SIGNALING_PATHWAYS	82	2.098	0.008	-1.061	0.363
WP_ALPHA_6_BETA_4_SIGNALING_PATHWAY	33	2.068	0.002	-2.619	0.000
WP_WNT_SIGNALING_PATHWAY	50	2.029	0.006	-1.161	0.271
WP_MRNA_PROCESSING	125	2.026	0.000	4.341	0.000
WP_JOUBERT_SYNDROME	76	1.999	0.010	-1.992	0.004
WP_ANDROGEN_RECEPTOR_SIGNALING_PATHWAY	90	1.941	0.006	-1.611	0.037
KEGG_NOTCH_SIGNALING_PATHWAY	47	1.929	0.004	-1.675	0.034
KEGG_LYSINE_DEGRADATION	43	1.927	0.005	-1.201	0.243
KEGG_CYSTEINE_AND_METHIONINE_METABOLISM	32	1.927	0.014	-1.371	0.134

WP_SRF_AND_MIRS_IN_SMOOTH_MUSCLE_DIFFERENTIATION_AND_PROLIFERATION	12	1.923	0.012	-1.922	0.010
WP_OSX_AND_MIRNAS_IN_TOOTH_DEVELOPMENT	18	1.923	0.006	-1.008	0.418
WP_TYPE_2_PAPILLARY_RENAL_CELL_CARCINOMA	33	1.906	0.008	-0.597	0.944
WP_GENES_TARGETED_BY_MIRNAS_IN_ADIPOCYTES	12	1.900	0.012	-1.925	0.013
WP_HEAD_AND_NECK_SQUAMOUS_CELL_CARCINOMA	73	1.889	0.012	-1.054	0.363
WP_HISTONE_MODIFICATIONS	61	1.882	0.008	-0.882	0.581
WP_ERBB_SIGNALING_PATHWAY	90	1.825	0.022	-1.511	0.069
WP_NEUROGENESIS_REGULATION_IN_THE_OLFACTORY_EPITHELIUM	56	1.818	0.011	-2.360	0.002
HALLMARK_MITOTIC_SPINDLE	199	1.789	0.012	1.645	0.018
WP_DISORDERS_OF_FRUCTOSE_METABOLISM	13	1.784	0.025	1.373	0.124
KEGG_ENDOMETRIAL_CANCER	52	1.775	0.014	-0.996	0.460
WP_DNA_REPAIR_PATHWAYS_FULL_NETWORK	118	1.761	0.017	3.878	0.000
WP_NEOVASCULARISATION_PROCESSES	37	1.751	0.026	-2.509	0.000
KEGG_SMALL_CELL_LUNG_CANCER	83	1.737	0.018	-1.984	0.002
KEGG_VASCULAR_SMOOTH_MUSCLE_CONTRACTION	112	1.734	0.011	-4.134	0.000
WP_AEROBIC_GLYCOLYSIS	12	1.734	0.017	1.291	0.161
KEGG_RENAL_CELL_CARCINOMA	70	1.724	0.024	-1.531	0.064
WP_ENERGY_METABOLISM	47	1.722	0.017	-1.692	0.028
WP_ATM_SIGNALING_IN_DEVELOPMENT_AND_DISEASE	45	1.718	0.031	1.062	0.395
WP_REGULATION_OF_WNT_BCATENIN_SIGNALING_BY_SMALL_MOLECULE_COMPOUNDS	17	1.686	0.028	-0.889	0.579
WP_SOMITOGENESIS_IN_THE_CONTEXT_OF_SPONDYLOCOSTAL_DYSOSTOSIS	9	1.669	0.026	1.255	0.204
WP_LNCRNA_IN_CANONICAL_WNT_SIGNALING_AND_COLORECTAL_CANCER	93	1.667	0.035	1.011	0.424
WP_ANGIOPOIETINLIKE_PROTEIN_8_REGULATORY_PATHWAY	130	1.664	0.041	-2.707	0.000
WP_TRANSCRIPTION_COFACTORS_SKI_AND_SKIL_PROTEIN_PARTNERS	18	1.660	0.032	-1.080	0.351
HALLMARK_HYPOXIA	198	1.655	0.026	-1.332	0.135

WP_THYROID_HORMONES_PRODUCTION_AND_PERIPHERAL_DOWNSTREAM_SIGNALING_EFFECTS	93	1.654	0.040	-1.727	0.022
WP_MIRNAS_INVOLVED_IN_DNA_DAMAGE_RESPONSE	35	1.630	0.049	1.817	0.010
WP_4249_HEDGEHOG_SIGNALING_PATHWAY	41	1.629	0.036	-1.897	0.012
WP_NOTCH1_REGULATION_OF_ENDOTHELIAL_CELL_CALCIFICATION	17	1.626	0.028	-1.846	0.016
WP_CORI_CYCLE	16	1.626	0.041	0.840	0.637
WP_MECHANOREGULATION_AND_PATHOLOGY_OF_YAPTAZ_VIA_HIPPO_AND_NONHIPPO_MECHANISMS	44	1.622	0.037	-1.437	0.091
KEGG_DORSO_VENTRAL_AXIS_FORMATION	22	1.606	0.040	-1.524	0.059
WP_CELL_DIFFERENTIATION_INDEX	22	1.5785693	0.05	-1.772809	0.03088803
KEGG_ERBB_SIGNALING_PATHWAY	87	1.567148	0.06090373	-1.582524	0.04106776
KEGG_MELANOGENESIS	99	1.565	0.048	-2.044	0.008
WP_BLADDER_CANCER	39	1.564	0.036	0.837	0.663
WP_ENDOMETRIAL_CANCER	63	1.560	0.044	-0.909	0.566
WP_RETT_SYNDROME_CAUSING_GENES	48	1.5572004	0.05231388	-1.801264	0.015904572
WP_MESODERMAL_COMMITMENT_PATHWAY	148	1.5513973	0.07099392	-1.445141	0.102564104
WP_BREAST_CANCER_PATHWAY	152	1.545	0.043	-1.308	0.148
KEGG_PROSTATE_CANCER	89	1.5417353	0.06854839	-0.777037	0.7292929
WP_OSTEOBLAST_DIFFERENTIATION_AND_RELATED_DISEASES	118	1.5218923	0.07894737	-3.295222	0
WP_HAIR_FOLLICLE_DEVELOPMENT_ORGANOGENESIS_PART_2_OF_3	32	1.5137861	0.06560636	-1.577914	0.051587302
WP_ID_SIGNALING_PATHWAY	16	1.509521	0.05858586	1.19573	0.2646503
WP_GLYCOLYSIS_IN_SENESCENCE	11	1.5021582	0.08413002	0.608966	0.9262948
WP_METHIONINE_METABOLISM_LEADING_TO_SULFUR_AMINO_ACIDS_AND_RELATED_DISORDERS	11	1.5021406	0.06142034	-1.215518	0.2209073
WP_NAD_BIOSYNTHETIC_PATHWAYS	22	1.4947909	0.06967213	-0.805771	0.69673705
WP_HIPPOYAP_SIGNALING_PATHWAY	21	1.4947209	0.08213552	-1.069149	0.3767821
WP_PKCGAMMA_CALCIUM_SIGNALING_PATHWAY_IN_ATAXIA	22	1.4815441	0.07407408	-2.025932	0.008196721
WP_INTEGRINMEDIATED_CELL_ADHESION	101	1.4797595	0.07378641	-3.083576	0
WP_DNA_IRDAMAGE_AND_CELLULAR_RESPONSE_VIA_ATR	81	1.478	0.056	3.407	0.000
WP_ERK_PATHWAY_IN_HUNTINGTONS_DISEASE	15	1.478	0.048	-0.704	0.836

WP_INFLUENCE_OF_LAMINOPATHIES_ON_WNT_SIGNALING	34	1.4693129	0.06638116	-0.851563	0.62601626
WP_SPLICING_FACTOR_NOVA_REGULATED_SYNAPTIC_PROTEINS	41	1.4572885	0.09034908	-1.645616	0.038910504
WP_PHOSPHOINOSITIDES_METABOLISM	48	1.4571955	0.07647059	-2.872668	0
WP_INITIATION_OF_TRANSCRIPTION_AND_TRANSLATION_ELONGATION_AT_THE_HIV1_LTR	32	1.4542667	0.07736944	-1.197879	0.24390244
WP_MAPK_CASCADE	33	1.4501737	0.08610568	-1.475622	0.080434784
KEGG_INOSITOL_PHOSPHATE_METABOLISM	54	1.4245585	0.09710744	-1.659325	0.027896997
WP_CANONICAL_AND_NONCANONICAL_NOTCH_SIGNALING	27	1.4197581	0.08798283	-2.221967	0.003898636
WP_MELATONIN_METABOLISM_AND_EFFECTS	33	1.415594	0.1010101	-1.613954	0.0513347
WP_OVARIAN_INFERTILITY	30	1.4115936	0.09645669	-1.342769	0.13111547
WP_NEPHROTIC_SYNDROME	44	1.4066311	0.11180124	-1.269289	0.16666667
WP_MAMMALIAN_DISORDER_OF_SEXUAL_DEVELOPMENT	24	1.4043293	0.10040984	-0.68426	0.87401575
WP_MALE_INFERTILITY	132	1.4023556	0.09607843	-0.842962	0.64624506
WP_METHIONINE_DE_NOVO_AND_SALVAGE_PATHWAY	22	1.401479	0.11324376	-0.569046	0.9738956
KEGG_GLYCOSAMINOGLYCAN_BIOSYNTHESIS_HEPARAN_SULFATE	26	1.3968266	0.11198428	-1.517782	0.07231405
WP_MECP2_AND_ASSOCIATED_RETT_SYNDROME	73	1.396297	0.09504132	-0.646	0.8926441
WP_MAMMARY_GLAND_DEVELOPMENT_PATHWAY_PREGNANCY_AND_LACTATION_STAGE_3_OF_4	33	1.3950433	0.1069307	-0.858593	0.6300578
WP_BMP_SIGNALING_IN_EYELID_DEVELOPMENT	20	1.3945495	0.1197479	-1.608485	0.046184737
WP_CELL_DIFFERENTIATION_EXPANDED_INDEX	29	1.3938936	0.11871228	-0.921822	0.56367433
WP_WNT_SIGNALING	112	1.3902873	0.10133843	-1.867709	0.009596929
KEGG_BLADDER_CANCER	41	1.3840907	0.10833333	0.822598	0.6954023
WP_GENE_REGULATORY_NETWORK_MODELLING_SOMITOGENESIS	11	1.3669906	0.136	0.797856	0.7244898
WP_MIRNA_REGULATION_OF_PROSTATE_CANCER_SIGNALING_PATHWAYS	34	1.3669871	0.10980392	-0.965658	0.47881356
WP_BRAINDERIVED_NEUROTROPHIC_FACTOR_BDNF_SIGNALING_PATHWAY	144	1.3593407	0.11445783	-2.34383	0
WP_DNA_DAMAGE_RESPONSE_ONLY_ATM_DEPENDENT	109	1.3579495	0.13184585	-1.335448	0.14836223

WP_NEURAL_CREST_CELL_MIGRATION_IN_CANCER	42	1.3503966	0.12989691	-2.4192	0
KEGG_NEUROTROPHIN_SIGNALING_PATHWAY	125	1.3468349	0.13307984	-1.958044	0.005988024
KEGG_GNRH_SIGNALING_PATHWAY	96	1.3405888	0.12269939	-2.554125	0
WP_EMBRYONIC_STEM_CELL_PLURIPOTENCY_PATHWAYS	116	1.3395439	0.13412228	-2.253467	0
KEGG_BASE_EXCISION_REPAIR	33	1.334	0.141	3.061	0.000
KEGG_BASAL_CELL_CARCINOMA	55	1.3325464	0.13052209	-0.800693	0.7096154
KEGG_FRUCTOSE_AND_MANNOSE_METABOLISM	33	1.3237286	0.14897579	-1.252669	0.18106996
WP_EGFEGFR_SIGNALING_PATHWAY	161	1.3112872	0.1680162	-2.412742	0
WP_NCRNAS_INVOLVED_IN_WNT_SIGNALING_IN_HEPATOCELLULAR_CARCINOMA	85	1.3086917	0.15151516	-0.788324	0.7198364
KEGG_COLORECTAL_CANCER	62	1.2983139	0.16221374	0.624005	0.922
WP_FAMILIAL_HYPERLIPIDEMIA_TYPE_4	21	1.2864301	0.15079366	-1.338039	0.1532567
WP_PANCREATIC_ADENOCARCINOMA_PATHWAY	88	1.2833071	0.18503937	0.611151	0.9266409
WP_MFAP5_EFFECT_ON_PERMEABILITY_AND_MOTILITY_OF_ENDOTHELIAL_CELLS_VIA_CYTOSKELETON_REARRANGEMENT	18	1.2755805	0.165692	-2.220528	0.001919386
WP_CONSTITUTIVE_ANDROSTANE_RECEPTOR_PATHWAY	27	1.2727547	0.17829457	-1.069576	0.34516767
WP_LEUCINE_ISOLEUCINE_AND_VALINE_METABOLISM	24	1.271294	0.17012449	-1.91026	0.010121457
WP_BASE_EXCISION_REPAIR	31	1.265	0.185	2.990	0.000
WP_WNT_SIGNALING_PATHWAY_AND_PLURIPOTENCY	100	1.2635528	0.18312757	-1.451782	0.086876154
WP_FRAGILE_X_SYNDROME	118	1.2599527	0.18106996	-1.883586	0.007604563
WP_FAMILIAL_HYPERLIPIDEMIA_TYPE_5	14	1.2586	0.19433199	-1.263069	0.19246031
WP_NUCLEOTIDE_EXCISION_REPAIR_IN_XERODERMA_PIGMENTOSUM	72	1.252	0.182	2.457	0.000
WP_INHIBITION_OF_EXOSOME_BIOGENESIS_AND_SECRETION_BY_MANUMYCIN_A_IN_CRPC_CELLS	18	1.248225	0.21984436	-0.970173	0.47907948
WP_BMP2WNT4FOXO1_PATHWAY_IN_PRIMARY_ENDOMETRIAL_STROMAL_CELL_DIFFERENTIATION	13	1.2454759	0.20731707	-0.799191	0.71343875
WP_DYRK1A	61	1.243112	0.18329939	1.042172	0.40234375
HALLMARK_MYC_TARGETS_V2	57	1.242	0.204	4.144	0.000

WP_DISRUPTION_OF_POSTSYNAPTIC_SIGNALING_BY_CNV	32	1.2419004	0.19795918	-1.117696	0.336714
WP_MAMMARY_GLAND_DEVELOPMENT_PATHWAY_INVOLUTION_STAGE_4_OF_4	10	1.2396836	0.20038536	1.411422	0.09073724
HALLMARK_P53_PATHWAY	199	1.2394952	0.18609408	1.572021	0.060240965
WP_MAMMARY_GLAND_DEVELOPMENT_PATHWAY_EMBRYONIC_DEVELOPMENT_STAGE_1_OF_4	17	1.2363372	0.20233463	-1.34195	0.13438736
WP_FOLLICLE_STIMULATING_HORMONE_FSH_SIGNALING_PATHWAY	24	1.2334075	0.20117188	-1.074214	0.36016098
KEGG_PEROXISOME	78	1.2323829	0.21428572	-1.497326	0.072
WP_SLEEP_REGULATION	38	1.2311649	0.20436507	-1.054303	0.36627907
WP_THYROID_STIMULATING_HORMONE_TSH_SIGNALING_PATHWAY	66	1.2308851	0.20428015	-1.004386	0.43326885
WP_ATM_SIGNALING_PATHWAY	40	1.231	0.217	2.053	0.002
WP_SEROTONIN_RECEPTOR_467_AND_NR3C_SIGNALING	19	1.2269849	0.21747968	-1.30246	0.167002
WP_ECTODERM_DIFFERENTIATION	136	1.2233648	0.2112403	-1.747165	0.022177419
WP_PI3KAKTMTOR_SIGNALING_PATHWAY_AND_THERAPEUTIC_OPPORTUNITIES	30	1.2186469	0.22661123	-1.233264	0.2020202
WP_HIPPOMERLIN_SIGNALING_DYSREGULATION	117	1.2180957	0.21836735	-2.559155	0
KEGG_PHOSPHATIDYLINOSITOL_SIGNALING_SYSTEM	75	1.2171986	0.2079395	-2.940479	0
KEGG_PANCREATIC_CANCER	70	1.2107599	0.22064777	-1.121968	0.3007968
HALLMARK_BILE_ACID_METABOLISM	112	1.2093861	0.22699386	-1.831497	0.015355086
WP_EGFR_TYROSINE_KINASE_INHIBITOR_RESISTANCE	84	1.2061865	0.22975518	-1.611912	0.050403226
WP_15Q112_COPY_NUMBER_VARIATION_SYNDROME	9	1.2033741	0.20718816	-1.543035	0.046747968
WP_HEART_DEVELOPMENT	44	1.1998407	0.22699386	-2.028603	0.002024292
WP_MICROTUBULE_CYTOSKELETON_REGULATION	46	1.1974752	0.198	-2.196867	0.002087683
WP_INSULIN_SIGNALING	159	1.1940402	0.23236515	-2.901487	0
WP_ANGIOGENESIS	24	1.193119	0.24652088	-2.348217	0
WP_RIBOFLAVIN_AND_COQ_DISORDERS	13	1.1912587	0.24603175	1.289012	0.15983607
WP_SMALL_CELL_LUNG_CANCER	94	1.1910478	0.23353294	-1.487755	0.0665362
WP_MTHFR_DEFICIENCY	24	1.1875517	0.26377952	-0.772242	0.75
WP_INTERLEUKIN1_IL1_STRUCTURAL_PATHWAY	49	1.1822815	0.24	1.06959	0.33052632
KEGG_MTOR_SIGNALING_PATHWAY	52	1.1784892	0.25536063	-1.714167	0.018072288

WP_PHOTODYNAMIC_THERAPYINDUCED_HIF1_SURVIVAL_SIGNALING	37	1.1706761	0.26572007	1.4425	0.072434604
WP_ESTROGEN_SIGNALING_PATHWAY	23	1.1697077	0.25636008	-1.899747	0.017307693
WP_FOXA2_PATHWAY	19	1.1693822	0.24180327	-1.084134	0.3407258
WP_PDGF_PATHWAY	39	1.168406	0.24893618	-1.509838	0.064449064
WP_REGULATION_OF_APOPTOSIS_BY_PARATHYROID_HORMONERELATED_PROTEIN	22	1.1626316	0.2753346	1.158645	0.27929688
WP_NRP1TRIGGERED_SIGNALING_PATHWAYS_IN_PANCREATIC_CANCER	54	1.1599119	0.26572007	-1.832571	0.015748031
KEGG_GLIOMA	64	1.1588279	0.26147705	-1.700543	0.021484375
WP_CELL_MIGRATION_AND_INVASION_THROUGH_P75NTR	30	1.1576452	0.2580645	-0.923461	0.5310621
KEGG_RIBOFLAVIN_METABOLISM	16	1.1565121	0.25851703	-1.262062	0.19246861
WP_FACTORS_AND_PATHWAYS_AFFECTING_INSULINLIKE_GROWTH_FACTOR_IGF1AKT_SIGNALING	36	1.1522789	0.2797619	-2.189852	0.003992016
WP_AMPLIFICATION_AND_EXPANSION_OF_ONCOGENIC_PATHWAYS_AS_METASTATIC_TRAITS	17	1.148987	0.27087575	-1.018103	0.4317719
WP_METASTATIC_BRAIN_TUMOR	12	1.1484745	0.27325583	1.232622	0.21218488
WP_FGF23_SIGNALING_IN_HYPOPHOSPHATEMIC_RICKETS_AND_RELATED_DISORDERS	22	1.1481485	0.275	-0.971855	0.48225468
WP_TYPE_I_COLLAGEN_SYNTHESIS_IN_THE_CONTEXT_OF_OSTEOGENESIS_IMPERFECTA	33	1.1473997	0.26597938	-2.133792	0.003952569
WP_THYROXINE_THYROID_HORMONE_PRODUCTION	25	1.1472529	0.26732674	-1.032143	0.42125985
KEGG_GAP_JUNCTION	84	1.1396222	0.2813102	-2.190957	0
WP_MAPK_PATHWAY_IN_CONGENITAL_THYROID_CANCER	16	1.1385171	0.30136988	-1.06154	0.36308315
WP_GPR40_PATHWAY	15	1.1326519	0.30528376	-1.545098	0.06275304
WP_ALSTROM_SYNDROME	33	1.1318021	0.29389313	-0.773371	0.7288136
KEGG_RETINOL_METABOLISM	44	1.1301874	0.28460038	0.905338	0.5966851
KEGG_WNT_SIGNALING_PATHWAY	147	1.1249878	0.29268292	-2.109158	0.003952569
WP_BDNFTRKB_SIGNALING	33	1.12437	0.3031579	-1.24441	0.19607843
WP_ASPIRIN_AND_MIRNAS	14	1.1226782	0.30181086	1.147945	0.26746505
WP_INTERLEUKIN11_SIGNALING_PATHWAY	44	1.1154332	0.3046729	-1.216972	0.21626984
WP_SMC1SMC3_ROLE_IN_DNA_DAMAGE_CORNELIA_DE_LANGE_SYNDROME	11	1.1094854	0.3292683	0.615141	0.918
WP_IL7_SIGNALING_PATHWAY	25	1.1088586	0.30408162	-0.790747	0.7093496
KEGG_TASTE_TRANSDUCTION	33	1.1044921	0.32553607	-1.851394	0.009940358

WP_MET_IN_TYPE_1_PAPILLARY_RENAL_CELL_CARCINOMA	58	1.1022247	0.3275194	-1.170823	0.27789474
KEGG_NUCLEOTIDE_EXCISION_REPAIR	43	1.100	0.323	2.795	0.000
WP_MAPK_AND_NFKB_SIGNALING_PATHWAYS_INHIBITED_BY_YERSINIA_YOPJ	12	1.0934929	0.32128513	-0.726965	0.8154158
WP_TRANSSULFURATION_PATHWAY	10	1.0932287	0.33401638	-1.373653	0.14345992
WP_IL17_SIGNALING_PATHWAY	32	1.0922945	0.34879032	-0.80691	0.6873748
WP_TAMOXIFEN_METABOLISM	16	1.090095	0.3436214	-1.691078	0.026104419
KEGG_UBIQUITIN_MEDIATED_PROTEOLYSIS	133	1.0752156	0.33396947	1.238354	0.21862349
WP_NUCLEOTIDE_EXCISION_REPAIR	43	1.075	0.348	2.852	0.000
WP_2Q112_COPY_NUMBER_VARIATION_SYNDROME	39	1.0733585	0.3732535	0.960235	0.488
WP_DEREGULATION_OF_RAB_AND_RAB_EFFECTOR_GENES_IN_BLADDER_CANCER	16	1.0721127	0.35387674	-1.679232	0.045454547
HALLMARK_ESTROGEN_RESPONSE_EARLY	197	1.0659357	0.34719336	-1.660391	0.036
KEGG_NON_SMALL_CELL_LUNG_CANCER	54	1.065783	0.36679536	-1.465076	0.068085104
WP_NONSMALL_CELL_LUNG_CANCER	72	1.0637219	0.35407725	0.934541	0.52772075
KEGG_CHRONIC_MYELOID_LEUKEMIA	73	1.0620376	0.36363637	-0.958266	0.49190283
WP_HOMOLOGOUS_RECOMBINATION	13	1.062	0.353	1.900	0.008
HALLMARK_HEDGEHOG_SIGNALING	36	1.0567951	0.3659889	-1.544375	0.044624746
WP_NUCLEAR_RECEPTORS	37	1.0519062	0.374	-1.894119	0.006147541
KEGG_THYROID_CANCER	29	1.0517799	0.3706004	1.170435	0.2664016
HALLMARK_NOTCH_SIGNALING	32	1.0511593	0.35743803	-2.085812	0.004081633
WP_H19_ACTION_RBE2F1_SIGNALING_AND_CDKBETACATENIN_ACTIVITY	15	1.0389389	0.3984221	1.581675	0.05533597
KEGG_PANTOTHENATE_AND_COA_BIOSYNTHESIS	15	1.0368488	0.37821782	-0.922897	0.5423387
WP_RAC1PAK1P38MMP2_PATHWAY	68	1.035759	0.39451477	-1.245057	0.20325203
WP_BONE_MORPHOGENIC_PROTEIN_SIGNALING_AND_REGULATION	12	1.0348203	0.39423078	-1.364228	0.138833
WP_ZINC_HOMEOSTASIS	27	1.0285871	0.4091778	-0.985056	0.48015872
WP_ETHANOL_EFFECTS_ON_HISTONE_MODIFICATIONS	29	1.0266662	0.38771594	-1.563204	0.05510204

WP_GALANIN_RECEPTOR_PATHWAY	20	1.0196844	0.38640776	-0.98111	0.4653846
KEGG_AMINOACYL_TRNA_BIOSYNTHESIS	41	1.017	0.402	2.184	0.000
WP_BILE_ACIDS_SYNTHESIS_AND_ENTEROHEPATIC_CIRCULATION	12	1.0138612	0.42632613	1.14878	0.26853707
WP_EFFECT_OF_PROGERIN_ON_GENES_INVOLVED_IN_HUTCHINSONGILFORD_PROGERIA_SYNDROME	34	1.013	0.433	2.205	0.004
KEGG_ACUTE_MYELOID_LEUKEMIA	57	1.004086	0.42372882	0.661748	0.8727634
WP_CYSTEINE_AND_METHIONINE_CATABOLISM	15	1.0031391	0.42829075	-1.986406	0.001937985
WP_PLURIPOTENT_STEM_CELL_DIFFERENTIATION_PATHWAY	48	1.0025024	0.44333997	-1.853675	0.021653544
WP_TRANSCRIPTION_FACTORS_REGULATE_MIRNAS_RELATED_TO_CARDIAC_HYPERTROPHY	9	0.9955378	0.45039684	-1.288783	0.172
WP_TGFBETA_SIGNALING_PATHWAY	132	0.994821	0.43866944	-1.477493	0.08637236
WP_CORTICOTROPINRELEASING_HORMONE_SIGNALING_PATHWAY	89	0.9943969	0.4476386	-1.435293	0.086419754
WP_GPR143_IN_MELANOCYTES_AND_RETINAL_PIGMENT_EPITHELIUM_CELLS	29	0.9864396	0.42125985	-1.414338	0.09185804
WP_TRANSCRIPTION_FACTOR_REGULATION_IN_ADIPOGENESIS	22	0.9861047	0.4569672	-2.062201	0
WP_WNT_SIGNALING_IN_KIDNEY_DISEASE	36	0.9856665	0.45418328	-1.76694	0.014925373
HALLMARK_APICAL_SURFACE	44	0.9792722	0.45173746	-1.945491	0.007619048
WP_GASTRIN_SIGNALING_PATHWAY	112	0.9764909	0.46875	-1.20372	0.2430279
WP_ALTERNATIVE_PATHWAY_OF_FETAL_ANDROGEN_SYNTHESIS	9	0.9722893	0.46502057	-0.725379	0.82725525
WP_PREGNANE_X_RECEPTOR_PATHWAY	27	0.9712682	0.4471058	-0.767181	0.76209676
WP_HEREDITARY_LEIOMYOMATOSIS_AND_RENAL_CELL_CARCINOMA_PATHWAY	20	0.9673453	0.474	0.829446	0.67474747
WP_MAP3K1_ROLE_IN_PROMOTING_AND_BLOCKING_GONADAL_DETERMINATION	16	0.9654205	0.46745563	0.789581	0.70434785
WP_INTRAFLAGELLAR_TRANSPORT_PROTEINS_BINDING_TO_DYNEIN	24	0.9642398	0.484556	-0.989345	0.47272727
KEGG_GLYCINE_SERINE_AND_THREONINE_METABOLISM	31	0.9597027	0.5041322	-0.90738	0.56458336
WP_OMEGA3_OMEGA6_FATTY_ACID_SYNTHESIS	15	0.9585003	0.49390244	-0.750399	0.802935

WP_VALPROIC_ACID_PATHWAY	12	0.9560578	0.49799198	-1.113823	0.2936345
WP_MITOCHONDRIAL_GENE_EXPRESSION	19	0.9535369	0.51361865	0.958488	0.49530956
WP_THERMOGENESIS	107	0.9514604	0.5073069	-2.658346	0
WP_SARSCOV2_B117_VARIANT_ANTAGONISES_INNATE_IMMUNE_ACTIVATION	9	0.9437664	0.51778656	0.850018	0.63779527
WP_SYNAPTIC_SIGNALING_PATHWAYS_ASSOCIATED_WITH_AUTISM_SPECTRUM_DISORDER	50	0.9433338	0.51836735	-1.610781	0.035639413
WP_INTERLEUKIN1_INDUCED_ACTIVATION_OF_NFKB	10	0.9431944	0.5182013	-0.681442	0.85
WP_COPPER_HOMEOSTASIS	44	0.9427838	0.48785424	-1.512586	0.06517312
WP_GLYOXYLATE_METABOLISM	11	0.9367378	0.50595236	0.780644	0.7405941
WP_NRF2_PATHWAY	135	0.9363626	0.4858871	-1.457771	0.09255533
WP_EPITHELIAL_TO_MESENCHYMAL_TRANSITION_IN_COLORECTAL_CANCER	157	0.9341551	0.5146443	-2.748023	0
KEGG_ADHERENS_JUNCTION	73	0.9304584	0.5480769	-1.525886	0.069767445
WP_SUDDEN_INFANT_DEATH_SYNDROME_SIDS_SUSCEPTIBILITY_PATHWAYS	154	0.9293914	0.5069307	-1.522343	0.05162524
WP_INTERACTOME_OF_POLYCOMB_REPRESSIVE_COMPLEX_2_PRC2	16	0.9262666	0.5502958	1.419873	0.10685484
WP_DOPAMINE_METABOLISM	12	0.9262651	0.5533981	-1.077213	0.3713693
WP_IMATINIB_AND_CHRONIC_MYELOID_LEUKEMIA	20	0.9215913	0.53862214	-1.08296	0.36157024
WP_TYPE_II_DIABETES_MELLITUS	21	0.9209117	0.5298507	-1.312183	0.14
WP_MAJOR_RECEPTORS_TARGETED_BY_EPINEPHRINE_AND_NOREPINEPHRINE	16	0.9146774	0.54805726	-2.304944	0
WP_IL5_SIGNALING_PATHWAY	40	0.9116491	0.56153846	-1.162211	0.2672065
WP_UREA_CYCLE_AND_RELATED_DISEASES	9	0.911	0.552	1.675	0.025
WP_ESTROGEN_RECEPTOR_PATHWAY	13	0.9098533	0.5719697	-1.3183	0.1643002
WP_TRANSCRIPTIONAL_ACTIVATION_BY_NRF2_IN_RESPONSE_TO_PHYTOCHEMICALS	14	0.9075491	0.56557375	-1.508315	0.053497944
WP_DNA_IRDOUBLE_STRAND_BREAKS_AND_CELLULAR_RESPONSE_VIA_ATM	55	0.902	0.573	2.843	0.000
WP_NAD_METABOLISM	16	0.9020475	0.57228917	0.837974	0.64547205
WP_NRF2ARE_REGULATION	22	0.9007124	0.5492958	-1.029164	0.38582677
WP_DEVELOPMENT_OF_URETERIC_COLLECTION_SYSTEM	59	0.9007023	0.56578946	-1.572717	0.06639839

WP_DRUG_INDUCTION_OF_BILE_ACID_PATHWAY	15	0.8991405	0.5807087	-0.79495	0.71153843
KEGG_LONG_TERM_DEPRESSION	68	0.8967164	0.5955285	-1.765155	0.023809524
WP_SREBF_AND_MIR33_IN_CHOLESTEROL_AND_LIPID_HOMEOSTASIS	17	0.8957847	0.55643564	-1.673416	0.03632887
WP_NAD_METABOLISM_SIRTUINS_AND_AGING	11	0.8916722	0.584	0.504479	0.9941748
WP_CARDIAC_HYPERTROPHIC_RESPONSE	53	0.8837777	0.59196615	-2.231307	0.004032258
KEGG_BIOSYNTHESIS_OF_UNSATURATED_FATTY_ACIDS	22	0.8788166	0.6054159	-0.855129	0.60956174
WP_KENNEDY_PATHWAY_FROM_SPHINGOLIPIDS	14	0.8782361	0.5884692	0.545547	0.97556007
WP_TUMOR_SUPPRESSOR_ACTIVITY_OF_SMARCB1	29	0.875	0.631	1.653	0.039
WP_INTRACELLULAR_TRAFFICKING_PROTEINS_INVOLVED_IN_CMT_NEUROPATHY	27	0.8704463	0.61811024	-1.402509	0.11788618
WP_GANGLIO_SPHINGOLIPID_METABOLISM	13	0.8654338	0.6104651	-1.324454	0.15748031
WP_FATTY_ACID_OMEGAOXIDATION	10	0.8644739	0.60365856	-0.861093	0.617357
KEGG_TAURINE_AND_HYPOTAURINE_METABOLISM	10	0.8576487	0.6582031	0.575921	0.96259844
WP_HIPPO_SIGNALING_REGULATION_PATHWAYS	95	0.8563453	0.6280488	-2.902571	0
WP_UREA_CYCLE_AND_METABOLISM_OF_AMINO_GROUPS	21	0.8543688	0.625	-1.126721	0.28104576
KEGG_ENDOCYTOSIS	175	0.8503769	0.6329897	-2.499703	0
WP_SARSCOV2_MITOCHONDRIAL_CHRONIC_OXIDATIVE_STRESS_AND_ENDOTHELIAL_DYSFUNCTION	26	0.8499992	0.6312057	1.031915	0.39694658
WP_ROLES_OF_CERAMIDES_IN_THE_DEVELOPMENT_OF_INSULIN_RESISTANCE	23	0.8497882	0.6523605	-1.35807	0.14166667
WP_ONECARBON_METABOLISM_AND_RELATED_PATHWAYS	50	0.8469922	0.6766467	-0.837466	0.64717346
KEGG_ASCORBATE_AND_ALDARATE_METABOLISM	17	0.8463898	0.654902	-1.617036	0.044806518
WP_TRANSCRIPTIONAL_CASCADE_REGULATING_ADIPOGENESIS	13	0.8398662	0.68016195	0.776638	0.75
WP_WHITE_FAT_CELL_DIFFERENTIATION	32	0.8397838	0.65217394	-0.704488	0.854
WP_PHOTODYNAMIC_THERAPYINDUCED_NFE2L2_NRF2_SURVIVAL_SIGNALING	23	0.8361964	0.67474747	-1.934031	0.004032258

KEGG_GLYCOSAMINOGLYCAN_BIOSYNTHESIS_KERATAN_SULFATE	15	0.8359806	0.65810275	-1.095832	0.31827113
WP_PREIMPLANTATION_EMBRYO	52	0.836	0.630	1.769	0.017
WP_ONCOSTATIN_M_SIGNALING_PATHWAY	65	0.834984	0.6863544	-1.379057	0.105675146
WP_MICRORNAS_IN_CARDIOMYOCYTE_HYPERTROPHY	87	0.8345432	0.66132265	-1.905122	0.008064516
WP_NICOTINE_EFFECT_ON_DOPAMINERGIC_NEURONS	21	0.8333936	0.6527778	1.168191	0.25413224
WP_CAMKK2_PATHWAY	33	0.8310419	0.6472946	-2.34548	0
KEGG_SELENOAMINO_ACID_METABOLISM	25	0.8300893	0.6695096	0.988447	0.44094488
WP_GASTRIC_ACID_PRODUCTION	9	0.8286223	0.6752988	-1.289469	0.1663286
WP_NODLIKE_RECEPTOR_NLR_SIGNALING_PATHWAY	9	0.8239679	0.6871287	-0.692355	0.87474746
WP_SUPRESSION_OF_HMGB1_MEDIATED_INFLAMMATION_BY_THBD	9	0.8149736	0.6904277	1.063313	0.36938775
WP_ONECARBON_METABOLISM	29	0.8147558	0.6979167	1.536845	0.078470826
WP_TGIF_DISRUPTION_OF_SHH_SIGNALING	9	0.8117133	0.70612246	0.766473	0.75342464
WP_CILIARY_LANDSCAPE	210	0.8077195	0.7318087	1.465983	0.07632094
WP_METABOLIC_REPROGRAMMING_IN_COLON_CANCER	42	0.8051855	0.7209302	1.248553	0.1783567
WP_PDGFRBETA_PATHWAY	29	0.8019373	0.71513945	-1.380709	0.118473895
WP_OLIGODENDROCYTE_SPECIFICATION_AND_DIFFERENTIATION_LEADING_TO_MYELIN_COMPONENTS_FOR_CNS	29	0.8010272	0.7002012	-1.179329	0.25052193
KEGG_GLYOXYLATE_AND_DICARBOXYLATE_METABOLISM	16	0.7993798	0.68247426	0.591389	0.9534413
WP_LEPTIN_SIGNALING_PATHWAY	76	0.7967598	0.7240704	-1.721438	0.020618556
WP_EXTRACELLULAR_VESICLEMEDIATED_SIGNALING_IN_RECIPIENT_CELLS	30	0.7967379	0.7514563	-1.83334	0.007858546
WP_1Q211_COPY_NUMBER_VARIATION_SYNDROME	25	0.7956009	0.7385892	-0.856852	0.6277228
WP_TGFBETA_RECEPTOR_SIGNALING_IN_SKELETAL_DYSPLASIAS	58	0.7947819	0.7083333	-1.388945	0.11500975
WP_TGFBETA_RECEPTOR_SIGNALING	54	0.7895244	0.7311609	-1.041029	0.38289204
WP_VITAMIN_D_METABOLISM	9	0.7890074	0.7148594	0.665683	0.8806262
WP_47_HEDGEHOG_SIGNALING_PATHWAY	16	0.7793435	0.71428573	0.904817	0.5551102
KEGG_TGF_BETA_SIGNALING_PATHWAY	85	0.7757276	0.75643563	-2.193397	0.003838772
WP_ENDOTHELIN_PATHWAYS	31	0.7747524	0.7154471	-2.195234	0

WP_HEMESYNTHESIS_DEFECTS_AND_PORPHYRIAS	9	0.7729882	0.750503	0.773191	0.7524558
WP_TRANSLATION_INHIBITORS_IN_CHRONICALLY_ACTIVATED_PDGFRA_CELLS	46	0.7714714	0.74951077	-0.697682	0.8522954
WP_TRANSSULFURATION_ONECARBON_METABOLISM_AND_RELATED_PATHWAYS	65	0.7699652	0.7484787	0.880047	0.5925156
WP_IL1_SIGNALING_PATHWAY	55	0.7690078	0.7520161	-0.999017	0.4431599
WP_ESTROGEN_METABOLISM	13	0.7658423	0.75670105	-1.315778	0.14785992
WP_P38_MAPK_SIGNALING_PATHWAY	35	0.7569	0.7755511	-0.79204	0.73558646
WP_ATR_SIGNALING	9	0.7542545	0.78723407	0.833327	0.638
WP_HEME_BIOSYNTHESIS	9	0.7532947	0.7692308	0.771683	0.7265306
WP_16P112_PROXIMAL_DELETION_SYNDROME	73	0.7528561	0.7829787	1.393462	0.11958763
WP_MFAP5MEDIATED_OVARIAN_CANCER_CELL_MOTILITY_AND_INVASIVENESS	13	0.7489176	0.8041825	-1.595373	0.034412954
KEGG_LIMONENE_AND_PINENE_DEGRADATION	10	0.7376961	0.8068411	-1.153115	0.26746505
WP_ENDODERM_DIFFERENTIATION	141	0.7344325	0.8016032	-0.954339	0.50390625
WP_ENDOPLASMIC_RETICULUM_STRESS_RESPONSE_IN_CORONAVIRUS_INFECTION	46	0.7310251	0.81632656	-1.05865	0.38047808
KEGG_STARCH_AND_SUCROSE_METABOLISM	39	0.7294692	0.79083663	-1.799566	0.01875
WP_AMPACTIVATED_PROTEIN_KINASE_SIGNALING	64	0.7284498	0.8090551	-2.157219	0.001926782
WP_AUTOPHAGY	30	0.7249963	0.82723576	-1.337082	0.13871635
HALLMARK_HEME_METABOLISM	191	0.7249889	0.8121086	-1.237843	0.18924303
WP_GLYCOLYSIS_AND_GLUCONEOGENESIS	42	0.720334	0.84136546	-0.930135	0.536
WP_GPCRS_CLASS_C_METABOTROPIC_GLUTAMATE_PHEROMONE	15	0.7203096	0.802682	0.912651	0.5540541
HALLMARK_KRAS_SIGNALING_DN	194	0.7197584	0.8211382	-1.601769	0.03469388
KEGG_HEDGEHOG_SIGNALING_PATHWAY	54	0.7155056	0.8376238	-1.428672	0.08858268
HALLMARK_GLYCOLYSIS	198	0.713092	0.8427419	1.119131	0.29540917
WP_OVERLAP_BETWEEN_SIGNAL_TRANSDUCTION_PATHWAYS_CONTRIBUTING_TO_LMNA_LAMINOPATHIES	54	0.7098718	0.851927	-1.185202	0.23339659
WP_SOMATIC_SEX_DETERMINATION	13	0.7058792	0.8097166	0.86264	0.6053719
KEGG_GLYCOSYLPHOSPHATIDYLINOSITOL_GPI_ANCHOR_BIOSYNTHESIS	25	0.6938716	0.818	1.334241	0.13412228

WP_HEPATOCYTE_GROWTH_FACTOR_RECEPTOR_SIGNALING	34	0.6892963	0.8523622	-1.354361	0.14971209
WP_HOSTPATHOGEN_INTERACTION_OF_HUMAN_CORONAVIRUSES_MAPK_SIGNALING	36	0.6862546	0.8507752	-1.238853	0.21181263
WP_PHOSPHODIESTERASES_IN_NEURONAL_FUNCTION	52	0.6858574	0.87966806	-1.399248	0.10359408
WP_TLR4_SIGNALING_AND_TOLERANCE	27	0.6788496	0.8884462	-0.820594	0.68725866
KEGG_TYROSINE_METABOLISM	38	0.6766807	0.85742974	1.473847	0.06526316
WP_EPO_RECEPTOR_SIGNALING	26	0.6755751	0.86796534	-0.569685	0.96767676
WP_BLOOD_CLOTTING_CASCADE	22	0.6708165	0.8693069	-1.212378	0.23188406
WP_VITAMIN_DSENSITIVE_CALCIUM_SIGNALING_IN_DEPRESSION	36	0.6684463	0.89336014	-1.031806	0.4016563
WP_LEPTININSULIN_SIGNALING_OVERLAP	17	0.6669373	0.90515465	-1.292229	0.17193677
WP_RANKLRANK_SIGNALING_PATHWAY	55	0.6568852	0.88439304	-1.5368	0.075510204
WP_RETINOL_METABOLISM	15	0.6567591	0.9042969	0.838361	0.66866267
KEGG_VALINE_LEUCINE_AND_ISOLEUCINE_DEGRADATION	43	0.6554282	0.89378756	-2.080878	0
KEGG_INSULIN_SIGNALING_PATHWAY	134	0.6553616	0.90438247	-2.514809	0
WP_NEURODEGENERATION_WITH_BRAIN_IRON_ACCUMULATION_NBIA_SUBTYPES_PATHWAY	44	0.6448022	0.9010309	-1.038053	0.39922482
WP_THIAMINE_METABOLIC_PATHWAYS	9	0.6353473	0.90786946	-0.593694	0.936345
KEGG_TYPE_II_DIABETES_MELLITUS	47	0.6104033	0.9250493	-1.645237	0.025590552
WP_STEROL_REGULATORY_ELEMENTBINDING_PROTEINS_SREBP_SIGNALING	70	0.6012203	0.9443299	-1.918676	0.007843138
WP_FLUOROPYRIMIDINE_ACTIVITY	33	0.5868521	0.93877554	0.922592	0.5610236
WP_MIRNA_REGULATION_OF_DNA_DAMAGE_RESPONSE	80	0.563	0.975	2.511	0.000
KEGG_GALACTOSE_METABOLISM	25	0.5575419	0.95953757	-0.573313	0.9636015
WP_TARGET_OF_RAPAMYCIN_SIGNALING	36	0.5557857	0.97341514	-1.910015	0.011695907
WP_QUERCETIN_AND_NFKB_AP1_INDUCED_APOPTOSIS	15	0.5349178	0.99021524	-0.799368	0.71548116
WP_PTF1A_RELATED_REGULATORY_PATHWAY	10	0.524364	0.9707113	-0.650285	0.8864971
WP_22Q112_COPY_NUMBER_VARIATION_SYNDROME	113	0.5120531	0.9841584	-1.216302	0.214
WP_GASTRIC_CANCER_NETWORK_2	30	0.453	0.992	2.104	0.004

WP_INTEGRATED_CANCER_PATHWAY	44	0.450	0.988	2.486	0.000
WP_IL9_SIGNALING_PATHWAY	17	-0.454934	1	-0.95701	0.49590164
WP_CHOLESTASIS	19	-0.503711	0.9835391	-1.519324	0.05859375
WP_CANNABINOID_RECEPTOR_SIGNALING	26	-0.519103	0.98651254	-1.446094	0.07509881
WP_CLOCKCONTROLLED_AUTOPHAGY_IN_BONE_METABOLISM	78	-0.553365	0.9766082	-1.880966	0.003875969
WP_IRON_METABOLISM_IN_PLACENTA	12	-0.555016	0.97077245	0.956603	0.4940476
WP_COMMON_PATHWAYS_UNDERLYING_DRUG_ADDICTION	40	-0.5766	0.9460501	-0.971255	0.4653846
WP_LIVER_X_RECEPTOR_PATHWAY	9	-0.58153	0.9482072	1.022112	0.41614908
WP_MIRNA_REGULATION_OF_P53_PATHWAY_IN_PROSTATE_CANCER	23	-0.582341	0.9670782	0.876018	0.61277443
WP_DISORDERS_OF_FOLATE_METABOLISM_AND_TRANSPORT	13	-0.601941	0.9417671	1.244006	0.19057377
WP_PRADERWILLI_AND_ANGELMAN_SYNDROME	58	-0.601999	0.9458333	-0.80932	0.67578125
WP_PROLACTIN_SIGNALING_PATHWAY	76	-0.603765	0.9386503	-1.267826	0.17097415
WP_DNA_DAMAGE_RESPONSE	66	-0.607	0.941	2.595	0.000
WP_VITAMIN_B12_DISORDERS	12	-0.608422	0.93237704	-0.921684	0.5532787
WP_ALTERED_GLYCOSYLATION_OF_MUC1_IN_TUMOR_MICROENVIRONMENT	9	-0.608731	0.94163424	1.208476	0.22924902
WP_CELLTYPE_DEPENDENT_SELECTIVITY_OF_CCK2R_SIGNALING	13	-0.609851	0.9325397	-2.669926	0
KEGG_PHENYLALANINE_METABOLISM	18	-0.60999	0.95445544	0.992857	0.46285716
WP_SEROTONIN_RECEPTOR_2_AND_ELKSRFGATA4_SIGNALING	20	-0.614509	0.93158954	-0.90263	0.5787402
WP_NCRNAS_INVOLVED_IN_STAT3_SIGNALING_IN_HEPATOCELLULAR_CARCINOMA	16	-0.617679	0.9392713	-1.300389	0.15705766
WP_UREA_CYCLE_AND_ASSOCIATED_PATHWAYS	24	-0.622705	0.93116635	-0.567364	0.9574899
KEGG_PENTOSE_PHOSPHATE_PATHWAY	26	-0.629412	0.9364754	1.11696	0.27954972
KEGG_LONG_TERM_POTENTIATION	68	-0.637872	0.9094488	-1.422287	0.0951417
WP_FARNESOID_X_RECEPTOR_PATHWAY	17	-0.638086	0.89411765	0.727507	0.79961467
KEGG_ABC_TRANSPORTERS	42	-0.653453	0.8767677	-1.387572	0.123732254
WP_NAD_METABOLISM_IN_ONCOGENEINDUCED_SENESCENCE_AND_MITOCHONDRIAL_DYSFUNCTIONASSOCIATED_SENESCENCE	23	-0.663668	0.88391036	-0.757304	0.7955912

WP_G_PROTEIN_SIGNALING_PATHWAYS	90	-0.670315	0.86614174	-2.727173	0
KEGG_VALINE_LEUCINE_AND_ISOLEUCINE_BIOSYNTHESIS	11	-0.672727	0.8447581	1.282818	0.2041237
WP_NUCLEAR_RECEPTORS_IN_LIPID_METABOLISM_AND_TOXICITY	33	-0.681607	0.85	-0.962865	0.4880478
KEGG_ETHER_LIPID_METABOLISM	32	-0.6902	0.85360825	-1.275724	0.17276423
WP_AUTOSOMAL_RECESSIVE_OSTEOPETROSIS_PATHWAYS	11	-0.690235	0.8449304	-0.796922	0.70689654
WP_GENES_CONTROLLING_NEPHROGENESIS	44	-0.696218	0.8486708	-1.720751	0.022
KEGG_NITROGEN_METABOLISM	22	-0.698585	0.8516129	-0.836674	0.6860707
WP_2Q37_COPY_NUMBER_VARIATION_SYNDROME	130	-0.709918	0.84261036	0.56475	0.9700599
WP_OXYSTEROLS_DERIVED_FROM_CHOLESTEROL	45	-0.71184	0.81458336	-0.821107	0.68495935
WP_CHROMOSOMAL_AND_MICROSATELLITE_INSTABILITY_IN_COLORECTAL_CANCER	72	-0.717	0.805	1.629	0.042
WP_MRNA_VACCINE_ACTIVATION_OF_DENDRITIC_CELL_AND_INDUCTION_OF_IFN1	10	-0.718848	0.8347107	-0.965479	0.46666667
WP_OMEGA9_FATTY_ACID_SYNTHESIS	14	-0.720191	0.81707317	-0.932149	0.5060241
WP_MODULATION_OF_PI3KAKTMTOR_SIGNALING_BY_BIOACTIVE_SPHINGOLIPIDS	14	-0.725777	0.8055556	-1.761623	0.022088353
WP_PI3KAKTMTOR_VITAMIN_D3_SIGNALING	22	-0.729196	0.8098039	-1.250719	0.17107943
WP_ANTIVIRAL_AND_ANTIINFLAMMATORY_EFFECTS_OF_NRF2_ON_SARSCOV2_PATHWAY	30	-0.729839	0.79962546	-1.35729	0.13967611
WP_NOVEL_INTRACELLULAR_COMPONENTS_OF_RIGILIKE_RECEPTOR_PATHWAY	56	-0.733132	0.77045906	0.774948	0.76371306
WP_PROTEOGLYCAN_BIOSYNTHESIS	18	-0.733594	0.8016194	-1.951893	0.005802708
WP_SEROTONIN_HTR1_GROUP_AND_FOS_PATHWAY	32	-0.743062	0.75775194	-1.272686	0.17097415
KEGG_N_GLYCAN_BIOSYNTHESIS	45	-0.744008	0.7852632	-1.007556	0.45192307
KEGG_FOLATE_BIOSYNTHESIS	10	-0.746569	0.8044807	1.320604	0.1511194
WP_OSTEOPONTIN_SIGNALING	13	-0.749407	0.77690804	-0.73879	0.76528597
WP_ETHANOL_METABOLISM_PRODUCTION_OF_ROS_BY_CYP2E1	9	-0.753043	0.76893204	-0.893503	0.5996055
WP_ENVELOPE_PROTEINS_AND_THEIR_POTENTIAL_ROLES_IN_EDMD_PHYSIOPATHOLOGY	44	-0.753314	0.76352704	-1.809842	0.021611001

WP_MITOCHONDRIAL_IMMUNE_RESPONSE_TO_SARSCOV2	31	-0.756654	0.75883573	0.682153	0.8580508
WP_PRION_DISEASE_PATHWAY	32	-0.75765	0.76620823	-1.131433	0.31692913
KEGG_ALANINE_ASPARTATE_AND_GLUTAMATE_METABOLISM	31	-0.759345	0.7954972	-0.837803	0.6392157
WP_MBDNF_AND_PROBDNF_REGULATION_OF_GABA_NEUROTRANSMISSION	36	-0.764502	0.7392996	-0.936934	0.5041494
WP_SARS_CORONAVIRUS_AND_INNATE_IMMUNITY	24	-0.772352	0.75101215	-1.174821	0.25301206
KEGG_DRUG_METABOLISM_CYTOCHROME_P450	53	-0.7782	0.76180696	-1.345923	0.1326923
KEGG_ONE_CARBON_POOL_BY_FOLATE	17	-0.779116	0.7408907	1.487893	0.07042254
WP_BIOMARKERS_FOR_PYRIMIDINE_METABOLISM_DISORDERS	15	-0.779239	0.73770493	1.564084	0.050526317
WP_TRIACYLGLYCERIDE_SYNTHESIS	22	-0.781869	0.7321063	-2.260974	0.005859375
WP_APOPTOSIS_MODULATION_BY_HSP70	19	-0.782187	0.718107	-0.604234	0.94153225
WP_DISORDERS_OF_GALACTOSE_METABOLISM	12	-0.787655	0.7267442	-1.071208	0.37574553
WP_GLUCURONIDATION	17	-0.791011	0.7186898	-1.580265	0.05009634
KEGG_HOMOLOGOUS_RECOMBINATION	27	-0.806	0.714	3.113	0.000
KEGG_ALPHA_LINOLENIC_ACID_METABOLISM	18	-0.81117	0.6964657	-0.960532	0.46361747
WP_NEUROINFLAMMATION_AND_GLUTAMATERGIC_SIGNALING	140	-0.816614	0.67951316	-1.894087	0.010060363
WP_NANOPARTICLE_TRIGGERED_AUTOPHAGIC_CELL_DEATH	23	-0.816769	0.6860707	-1.083179	0.34349594
WP_OSTEOCLAST_SIGNALING	16	-0.816965	0.6778656	-0.672761	0.87116563
WP_PYRIMIDINE_METABOLISM_AND_RELATED_DISEASES	17	-0.817	0.699	1.637	0.038
WP_TRYPTOPHAN_METABOLISM	33	-0.820715	0.6738281	-0.808203	0.70520234
KEGG_RIG_I_LIKE_RECEPTOR_SIGNALING_PATHWAY	60	-0.82074	0.6791667	1.161167	0.25813007
WP_CARDIAC_PROGENITOR_DIFFERENTIATION	52	-0.822176	0.688588	-1.666785	0.03258656
WP_APOPTOSISRELATED_NETWORK_DUE_TO_ALTERED_NOTCH3_IN_OVARIAN_CANCER	52	-0.830543	0.66536206	-1.27804	0.18395303
KEGG_PROPANOATE_METABOLISM	30	-0.833863	0.6485944	-1.834428	0.01119403
KEGG_GLYCOSAMINOGLYCAN_BIOSYNTHESIS_CHONDROITIN_SULFATE	22	-0.834708	0.6779661	-2.681217	0
WP_7Q1123_COPY_NUMBER_VARIATION_SYNDROME	101	-0.838856	0.6659794	1.475355	0.08227848

WP_SEROTONIN_AND_ANXIETYRELATED_EVENTS	13	-0.839218	0.6909091	-1.149451	0.2822086
HALLMARK_TGF_BETA_SIGNALING	54	-0.83989	0.634	-1.574259	0.04158004
WP_RENINANGIOTENSINALDOSTERONE_SYSTEM_RAAS	42	-0.840299	0.6455445	-2.191581	0.006276151
WP_GDNFRET_SIGNALING_AXIS	23	-0.840728	0.664	0.641799	0.9050505
KEGG_GLYCEROPHOSPHOLIPID_METABOLISM	75	-0.841263	0.6633858	-1.904777	0.00610998
WP_SARSCOV2_AND_COVID19_PATHWAY	9	-0.841464	0.6596154	-1.152846	0.26785713
WP_INCLUSION_BODY_MYOSITIS	11	-0.842702	0.66468257	-1.398005	0.104627766
WP_VITAMIN_B12_METABOLISM	47	-0.844088	0.6227898	-1.484544	0.07254902
KEGG_SPLICEOSOME	123	-0.855	0.663	5.724	0.000
KEGG_METABOLISM_OF_XENOBIOTICS_BY_CYTOCHROME_P450	53	-0.86154	0.668008	0.701678	0.87974685
WP_AGERAGE_PATHWAY	66	-0.863911	0.63747454	-1.205084	0.23031496
WP_TRYPTOPHAN_CATABOLISM_LEADING_TO_NAD_PRODUCTION	16	-0.864262	0.62033194	-0.982755	0.44376278
KEGG_PORPHYRIN_AND_CHLOROPHYLL_METABOLISM	32	-0.865252	0.5924453	-0.77865	0.7387755
WP_ARRHYTHMOGENIC_RIGHT_VENTRICULAR_CARDIOMYOPATHY	74	-0.869453	0.62620425	-2.448988	0
WP_SULFATION_BIOTRANSFORMATION_REACTION	13	-0.872313	0.59009904	0.808098	0.7112069
KEGG_SULFUR_METABOLISM	10	-0.874462	0.6003976	-1.61145	0.04950495
HALLMARK_ANDROGEN_RESPONSE	99	-0.875772	0.5944882	-1.592655	0.04809619
KEGG_PENTOSE_AND_GLUCURONATE_INTERCONVERSIONS	19	-0.8791	0.6	1.041486	0.4082474
WP_2Q211_COPY_NUMBER_VARIATION_SYNDROME	13	-0.8792	0.5966736	-1.19097	0.24902724
KEGG_ARRHYTHMOGENIC_RIGHT_VENTRICULAR_CARDIOMYOPATHY_ARVC	74	-0.879843	0.59607846	-2.467033	0
WP_BIOTIN_METABOLISM_INCLUDING_IMDS	9	-0.885547	0.6077236	-2.318667	0
WP_FOLATE_METABOLISM	65	-0.885681	0.59498954	-0.886553	0.5763359
WP_REGUCALCIN_IN_PROXIMAL_TUBULE_EPITHELIAL_KIDNEY_CELLS	29	-0.886202	0.5841996	-1.292718	0.17425743
WP_ENDOCHONDRAL_OSSIFICATION_WITH_SKELETAL_DYSPLASIAS	61	-0.892435	0.5906433	-2.612528	0
WP_VITAMIN_A_AND_CAROTENOID_METABOLISM	41	-0.892937	0.5967078	-0.791826	0.743083
WP_MONOAMINE_TRANSPORT	32	-0.89355	0.5842217	-1.513905	0.06367041
WP_P53_TRANSCRIPTIONAL_GENE_NETWORK	88	-0.896956	0.56092435	-1.281324	0.17529881
WP_INTERFERON_TYPE_I_SIGNALING_PATHWAYS	54	-0.898783	0.55772996	-0.898722	0.57494867

WP_OXIDATIVE_STRESS_RESPONSE	31	-0.901638	0.55122954	-1.352019	0.14430894
WP_S1P_RECEPTOR_SIGNAL_TRANSDUCTION	25	-0.903187	0.5659229	-1.213922	0.21327968
WP_ENDOCHONDRAL_OSSIFICATION	61	-0.903665	0.5390782	-2.652457	0
WP_SEROTONIN_AND_ANXIETY	17	-0.904197	0.53305787	-1.217021	0.23047619
WP_KISSPEPTINKISSPEPTIN_RECEPTOR_SYSTEM_IN_THE_OVARY	37	-0.904602	0.55193484	-1.008981	0.42105263
WP_NITRIC_OXIDE_METABOLISM_IN_CYSTIC_FIBROSIS	13	-0.905928	0.5502958	1.180517	0.23287672
WP_COHESIN_COMPLEX_CORNELIA_DE_LANGE_SYNDROME	34	-0.911	0.546	1.589	0.040
WP_HIPPOCAMPAL_SYNAPTOGENESIS_AND_NEUROGENESIS	27	-0.911743	0.5711501	-1.827196	0.013645224
WP_MITOCHONDRIAL_LONG_CHAIN_FATTY_ACID_BETAOXIDATION	17	-0.912351	0.56494844	-1.68916	0.024539877
WP_PARKINSONS_DISEASE_PATHWAY	50	-0.916546	0.55625	1.258022	0.19656488
KEGG_NICOTINATE_AND_NICOTINAMIDE_METABOLISM	24	-0.917465	0.54451346	0.66831	0.90160644
KEGG_GLYCOLYSIS_GLUCONEOGENESIS	54	-0.917815	0.5223301	-0.734392	0.7937743
WP_HOSTPATHOGEN_INTERACTION_OF_HUMAN_CORONAVIRUSES_AUTOPHAGY	19	-0.918607	0.5610687	-0.91058	0.5513308
WP_EXERCISEINDUCED_CIRCADIAN_REGULATION	48	-0.920318	0.5276074	0.867299	0.6046025
WP_SPHINGOLIPID_METABOLISM_INTEGRATED_PATHWAY	26	-0.923955	0.5179704	-1.35227	0.114754096
WP_VASOPRESSINREGULATED_WATER_REABSORPTION	43	-0.92506	0.53521127	-1.111769	0.30871212
WP_HYPOTHESIZED_PATHWAYS_IN_PATHOGENESIS_OF_CARDIOVASCULAR_DISEASE	24	-0.927469	0.5416667	-2.621035	0
KEGG_ARGININE_AND_PROLINE_METABOLISM	51	-0.939311	0.53061223	-1.547191	0.04950495
KEGG_P53_SIGNALING_PATHWAY	65	-0.943975	0.51434034	1.560732	0.05102041
WP_MAMMARY_GLAND_DEVELOPMENT_PATHWAY_PUBERTY_STAGE_2_OF_4	13	-0.945491	0.49616858	-1.434682	0.1021611
KEGG_MISMATCH_REPAIR	22	-0.946	0.521	3.784	0.000
WP_SIGNAL_TRANSDUCTION_THROUGH_IL1R	32	-0.950622	0.5041494	0.751262	0.793456
WP_UNFOLDED_PROTEIN_RESPONSE	23	-0.951388	0.51356995	1.126143	0.31536925
WP_7OXOC_AND_7BETAHC_PATHWAYS	27	-0.9519	0.47808766	-0.654076	0.8895349

WP_MELANOMA	67	-0.952361	0.48054475	0.8564	0.63600785
KEGG_GLYCOSAMINOGLYCAN_DEGRADATION	21	-0.958612	0.46450305	-1.787596	0.019313306
WP_AIRWAY_SMOOTH_MUSCLE_CELL_CONTRACTION	16	-0.958875	0.47328246	-2.283457	0.001956947
WP_NANOMATERIAL_INDUCED_APOPTOSIS	20	-0.95947	0.49615383	1.339714	0.14693877
KEGG_BASAL_TRANSCRIPTION_FACTORS	34	-0.960	0.484	2.377	0.000
WP_FAS_LIGAND_PATHWAY_AND_STRESS_INDUCTION_OF_HEAT_SHOCK_PROTEINS	43	-0.961207	0.48915187	1.129172	0.28598848
KEGG_MELANOMA	71	-0.961322	0.4877551	-0.914989	0.542
KEGG_TIGHT_JUNCTION	128	-0.964261	0.48622048	-1.546138	0.05068226
WP_GLUTATHIONE_METABOLISM	19	-0.965082	0.4919355	-1.165951	0.24746451
WP_IL4_SIGNALING_PATHWAY	54	-0.966965	0.46336633	-1.340165	0.13663366
WP_STING_PATHWAY_IN_KAWASAKILIKE_DISEASE_AND_COVID19	21	-0.971461	0.49203187	-0.710038	0.8376238
WP_PHYSICOCHEMICAL_FEATURES_AND_TOXICITYASSOCIATED_PATHWAYS	65	-0.976396	0.47876447	-1.788305	0.021912351
WP_FOLATEALCOHOL_AND_CANCER_PATHWAY_HYPOTHESES	9	-0.977803	0.46985447	-0.597514	0.94057375
WP_MONOAMINE_GPCRS	31	-0.981819	0.4738878	-1.621334	0.057581574
WP_2873_ARYL_HYDROCARBON_RECEPTOR_PATHWAY	42	-0.983064	0.4576613	0.95563	0.47460938
WP_OVERVIEW_OF_INTERFERONSMEDIATED_SIGNALING_PATHWAY	26	-0.984626	0.45167652	-1.085607	0.35420743
WP_VITAMIN_K_METABOLISM_AND_ACTIVATION_OF_DEPENDENT_PROTEINS	12	-0.985009	0.4631579	-1.115912	0.29622266
WP_PHOTODYNAMIC_THERAPYINDUCED_AP1_SURVIVAL_SIGNALING	50	-0.985923	0.44282743	1.21915	0.20408164
WP_LIPID_METABOLISM_PATHWAY	28	-0.986099	0.47358122	-2.516772	0
KEGG_RNA_DEGRADATION	56	-0.988	0.439	2.628	0.000
KEGG_BUTANOATE_METABOLISM	33	-0.9894	0.46106556	-1.211981	0.19957983
WP_NUCLEAR_RECEPTORS_METAPATHWAY	302	-0.992058	0.4226804	-1.492163	0.08108108
WP_IL2_SIGNALING_PATHWAY	42	-0.992981	0.41472867	-1.016237	0.41468254
WP_EBSTEINBARR_VIRUS_LMP1_SIGNALING	22	-0.996299	0.45957446	1.468885	0.08858268
KEGG_GLYCEROLIPID_METABOLISM	48	-0.999173	0.4529058	-1.713914	0.01871102
WP_BIOMARKERS_FOR_UREA_CYCLE_DISORDERS	12	-0.999278	0.4378819	1.050947	0.37816763
WP_KIT_RECEPTOR_SIGNALING_PATHWAY	59	-1.000112	0.4280079	-1.459392	0.08086785

WP_EV_RELEASE_FROM_CARDIAC_CELLS_AND_THEIR_FUNCTIONAL_EFFECTS	9	-1.001188	0.4460888	-0.692006	0.8392157
KEGG_DRUG_METABOLISM_OTHER_ENZYMES	39	-1.002312	0.42769858	1.308684	0.16568047
WP_NEUROINFLAMMATION	11	-1.005257	0.41295546	1.188675	0.2548638
WP_15Q133_COPY_NUMBER_VARIATION_SYNDROME	14	-1.008208	0.43125	-0.929706	0.50715744
WP_FGFR3_SIGNALING_IN_CHONDROCYTE_PROLIFERATION_AND_TERMINAL_DIFFERENTIATION	27	-1.008307	0.44509804	-0.866335	0.6128405
KEGG_GLYCOSPHINGOLIPID_BIOSYNTHESIS_GANGLIO_SERIES	15	-1.023027	0.4135021	-2.31353	0
WP_HEPATITIS_C_AND_HEPATOCELLULAR_CARCINOMA	51	-1.024131	0.39839035	-1.515611	0.07184466
WP_KYNURENINE_PATHWAY_AND_LINKS_TO_CELL_SENESCENCE	23	-1.02481	0.4039604	0.783566	0.7565392
WP_PROGERIAASSOCIATED_LIPODYSTROPHY	22	-1.025813	0.39641434	-1.368568	0.11764706
WP_2586_ARYL_HYDROCARBON_RECEPTOR_PATHWAY	46	-1.029964	0.39388144	-0.785316	0.7229862
KEGG_VASOPRESSIN_REGULATED_WATER_REABSORPTION	43	-1.030749	0.41379312	-1.220205	0.22403258
WP_IL6_SIGNALING_PATHWAY	42	-1.032139	0.42028984	-1.388463	0.119521916
WP_3Q29_COPY_NUMBER_VARIATION_SYNDROME	59	-1.033428	0.38947368	1.025443	0.38036808
KEGG_ALDOSTERONE_REGULATED_SODIUM_REABSORPTION	42	-1.035797	0.386	-2.404551	0
WP_4HYDROXYTAMOXIFEN_DEXAMETHASONE_AND_RETINOIC_ACIDS_REGULATION_OF_P27_EXPRESSION	18	-1.038269	0.3814433	-1.08355	0.3257732
KEGG_REGULATION_OF_AUTOPHAGY	28	-1.044391	0.37747034	-0.861271	0.6424242
WP_PATHWAYS_OF_NUCLEIC_ACID_METABOLISM_AND_INNATE_IMMUNE_SENSING	15	-1.04592	0.38963532	1.271477	0.16907217
WP_NETRINUNC5B_SIGNALING_PATHWAY	52	-1.049204	0.3547718	-1.927034	0.005524862
WP_NEURAL_CREST_CELL_MIGRATION_DURING_DEVELOPMENT	39	-1.051274	0.37711865	-2.214151	0.004081633
WP_KALLMANN_SYNDROME	23	-1.05173	0.37022132	-0.603062	0.92957747
WP_PHYSIOLOGICAL_AND_PATHOLOGICAL_HYPERTROPHY_OF_THE_HEART	24	-1.0551	0.38247013	-1.204641	0.2312253
WP_LTF_DANGER_SIGNAL_RESPONSE_PATHWAY	17	-1.055715	0.37424546	1.340946	0.12890625

HALLMARK_UNFOLDED_PROTEIN_RESPONSE	113	-1.058	0.368	1.985	0.004
WP_CCL18_SIGNALING_PATHWAY	35	-1.061922	0.35984096	-1.876146	0.013861386
WP_TGFBETA_SIGNALING_IN_THYROID_CELLS_FOR_EPITHELIALMESENCHYMAL_TRANSITION	17	-1.06512	0.35329342	-1.206499	0.22440945
WP_FAMILIAL_HYPERLIPIDEMIA_TYPE_1	17	-1.065358	0.36039603	-1.432278	0.09437751
KEGG_VIBRIO_CHOLERAE_INFECTION	53	-1.072286	0.35976788	0.670106	0.88247013
WP_ALANINE_AND_ASPARTATE_METABOLISM	12	-1.081913	0.35684648	-0.568433	0.9529412
WP_CONGENITAL_GENERALIZED_LIPODYSTROPHY	18	-1.085159	0.34274954	-2.039895	0.002053388
WP_GPCRS_CLASS_B_SECRETINLIKE	22	-1.090252	0.34184676	-2.001046	0
WP_G13_SIGNALING_PATHWAY	38	-1.091523	0.3298969	-0.921153	0.55
WP_METHYLATION_PATHWAYS	9	-1.09182	0.33333334	-1.109523	0.31526104
WP_DNA_MISMATCH_REPAIR	23	-1.094	0.333	4.157	0.000
WP_ANGIOTENSIN_II_RECEPTOR_TYPE_1_PATHWAY	28	-1.096267	0.32251522	-2.501689	0
KEGG_GLUTATHIONE_METABOLISM	49	-1.10122	0.31376517	0.668926	0.8654618
WP_DOPAMINERGIC_NEUROGENESIS	29	-1.112056	0.3084886	-0.593192	0.94274026
KEGG_MATURITY_ONSET_DIABETES_OF_THE_YOUNG	24	-1.117014	0.30588236	0.956141	0.5
WP_SPHINGOLIPID_METABOLISM_OVERVIEW	23	-1.11789	0.2866242	-1.302245	0.14078675
HALLMARK_PROTEIN_SECRETION	95	-1.120476	0.2945591	-1.533676	0.07157464
WP_CLASSICAL_PATHWAY_OF_STEROIDOGENESIS_WITH_GLUCOCORTICOID_AND_MINERALOCORTICOID_METABOLISM	15	-1.126446	0.30227745	-1.15517	0.25858587
WP_REGULATION_OF_SISTER_CHROMATID_SEPARATION_AT_THE_METAPHASE ANAPHASE_TRANSITION	15	-1.128	0.308	2.590	0.000
WP_GLYCOGEN_SYNTHESIS_AND_DEGRADATION	38	-1.129785	0.30058938	-1.601943	0.041501977
KEGG_TERPENOID_BACKBONE_BIOSYNTHESIS	15	-1.139	0.272	1.729	0.016
KEGG_LINOLEIC_ACID_METABOLISM	24	-1.143794	0.26035503	-0.53654	0.96728015
KEGG_ADIPOCYTOKINE_SIGNALING_PATHWAY	66	-1.146011	0.28629032	-1.51109	0.07385229
WP_HOSTPATHOGEN_INTERACTION_OF_HUMAN_CORONAVIRUSES_INTERFERON_INDUCTION	33	-1.146969	0.27833003	-0.978908	0.4631783

WP_GLUCOCORTICOID_RECEPTOR_PATHWAY	68	-1.15221	0.27722773	-0.744216	0.78313255
HALLMARK_ESTROGEN_RESPONSE_LATE	198	-1.152277	0.28070176	-1.160835	0.275
WP_SEROTONIN_TRANSPORTER_ACTIVITY	11	-1.161292	0.26652452	-0.925128	0.53346455
WP_PROXIMAL_TUBULE_TRANSPORT	56	-1.162016	0.257874	-0.603579	0.94534415
KEGG_RENIN_ANGIOTENSIN_SYSTEM	17	-1.163769	0.25614753	-1.851069	0.008350731
WP_PTDINS45P2_IN_CYTOKINESIS_PATHWAY	12	-1.165348	0.2696177	0.919606	0.54285717
WP_NOCGMPPKG_MEDIATED_NEUROPROTECTION	47	-1.167846	0.25992063	-1.115326	0.30897704
WP_RELATIONSHIP_BETWEEN_INFLAMMATION_COX2_AND_EGFR	25	-1.17651	0.24752475	-0.83521	0.6818182
WP_REGULATION_OF_ACTIN_CYTOSKELETON	149	-1.179062	0.23883495	-3.164742	0
KEGG_TRYPTOPHAN_METABOLISM	40	-1.185882	0.23265307	-1.612999	0.059880238
KEGG_BETA_ALANINE_METABOLISM	22	-1.18639	0.26335877	-1.36647	0.14373717
WP_GLYCEROLIPIDS_AND_GLYCEROPHOSPHOLIPIDS	22	-1.186958	0.25346535	-1.173629	0.24654832
WP_HEPATITIS_B_INFECTION	142	-1.191023	0.2254902	-0.979251	0.47619048
WP_GLYCEROPHOSPHOLIPID_BIOSYNTHETIC_PATHWAY	29	-1.191903	0.25544554	-0.790096	0.7300216
WP_OSTEOBLAST_SIGNALING	12	-1.19692	0.22222222	-1.82808	0.020120725
WP_DEVELOPMENT_OF_PULMONARY_DENDRITIC_CELLS_AND_MACROPHAGE_SUBSETS	13	-1.197714	0.22310758	-1.021034	0.39641434
KEGG_CYTOSOLIC_DNA_SENSING_PATHWAY	46	-1.202	0.218	1.752	0.016
WP_DEGRADATION_PATHWAY_OF_SPHINGOLIPIDS_INCLUDING_DISEASES	14	-1.203404	0.24855492	-0.911654	0.56751466
WP_NONHOMOLOGOUS_END_JOINING	10	-1.20724	0.23217922	-0.992159	0.43661973
WP_SYNAPTIC_VESICLE_PATHWAY	50	-1.207256	0.24055666	-1.414534	0.09325397
WP_TCA_CYCLE_IN_SENESCENCE	10	-1.209301	0.21325052	-0.90922	0.5626204
KEGG_PYRUVATE_METABOLISM	38	-1.209307	0.23373984	-1.18183	0.25868726
WP_TNFALPHA_SIGNALING_PATHWAY	91	-1.20988	0.20507812	1.408107	0.08414873
WP_STEROID_BIOSYNTHESIS	9	-1.221898	0.225	0.770972	0.74455446
WP_RESISTIN_AS_A_REGULATOR_OF_INFLAMMATION	32	-1.22221	0.22834645	-1.447438	0.09202454
WP_MIRNAS_INVOLVEMENT_IN_THE_IMMUNE_RESPONSE_IN_SEPSIS	44	-1.227034	0.21722114	-0.895415	0.5879917

HALLMARK_SPERMATOGENESIS	131	-1.22812	0.22222222	1.358963	0.14166667
WP_RESOLVIN_E1_AND_RESOLVIN_D1_SIGNALING_PATHWAYS_PROMOTING_INFLAMMATION_RESOLUTION	12	-1.230076	0.22222222	-1.232901	0.21836735
WP_EUKARYOTIC_TRANSCRIPTION_INITIATION	41	-1.232	0.202	3.064	0.000
KEGG_PURINE_METABOLISM	156	-1.233	0.214	2.313	0.000
WP_BIOSYNTHESIS_AND_REGENERATION_OF_TETRAHYDROBIOPTERIN_AND_CATABOLISM_OF_PHENYLALANINE	10	-1.235534	0.19444445	-0.897259	0.58984375
WP_METAPATHWAY_BIOTRANSFORMATION_PHASE_I_AND_II	154	-1.237043	0.18126273	-0.854509	0.6338912
HALLMARK_UV_RESPONSE_DN	143	-1.244116	0.18495935	-4.120374	0
WP_PPARALPHA_PATHWAY	25	-1.249702	0.20537429	0.445898	0.99789476
WP_FIBRIN_COMPLEMENT_RECEPTOR_3_SIGNALING_PATHWAY	43	-1.25576	0.18571429	-0.980903	0.4466403
KEGG_ARACHIDONIC_ACID_METABOLISM	55	-1.257399	0.1873805	0.769787	0.77
KEGG_STEROID_BIOSYNTHESIS	17	-1.261157	0.19136961	1.42254	0.09710744
HALLMARK_PEROXISOME	103	-1.262977	0.20746888	0.795137	0.7104247
KEGG_GLYCOSPHINGOLIPID_BIOSYNTHESIS_GLOBO_SERIES	14	-1.264995	0.19721116	-1.09138	0.34942085
WP_GABA_RECEPTOR_SIGNALING	29	-1.265439	0.18128654	1.32077	0.15503876
WP_GLYCOSYLATION_AND_RELATED_CONGENITAL_DEFECTS	25	-1.266864	0.18525897	1.482044	0.086065575
WP_FATTY_ACID_BIOSYNTHESIS	22	-1.274117	0.182	-1.74955	0.02303263
WP_AMINO_ACID_METABOLISM	91	-1.277208	0.17333333	-1.687031	0.02964427
WP_GLYCOSAMINOGLYCAN_DEGRADATION	17	-1.285142	0.15873016	-1.720218	0.028571429
WP_DRAVET_SYNDROME	24	-1.290731	0.1796875	-2.150262	0.001956947
WP_PURINE_METABOLISM_AND_RELATED_DISORDERS	22	-1.297626	0.15927419	1.13669	0.28710938
WP_OXIDATION_BY_CYTOCHROME_P450	53	-1.298246	0.16054158	0.97605	0.475
WP_EDA_SIGNALING_IN_HAIR_FOLLICLE_DEVELOPMENT	13	-1.299367	0.17043121	1.32299	0.15049505
WP_EICOSANOID_SYNTHESIS	23	-1.300067	0.15145229	-1.181232	0.2672065
KEGG_AXON_GUIDANCE	129	-1.301522	0.16824196	-2.810728	0
KEGG_STEROID_HORMONE_BIOSYNTHESIS	42	-1.307742	0.15234375	1.152049	0.28803244
WP_FAMILIAL_HYPERLIPIDEMIA_TYPE_3	12	-1.308445	0.15368421	-1.625418	0.05
WP_CALCIUM_REGULATION_IN_CARDIAC_CELLS	149	-1.312738	0.16795367	-3.046044	0

WP_METABOLISM_OF_SPINGOLIPIDS_IN_ER_AND_GOLGI_APPARATUS	21	-1.320522	0.14989734	-1.473725	0.095634095
KEGG_NON_HOMOLOGOUS_END_JOINING	13	-1.320984	0.13518886	0.578241	0.96363634
WP_FAMILIAL_HYPERLIPIDEMIA_TYPE_2	15	-1.323731	0.16470589	-1.246225	0.1826923
WP_AMYOTROPHIC_LATERAL_SCLEROSIS_ALS	38	-1.330247	0.13017751	-1.620203	0.04206501
WP_TRANSLATION_FACTORS	50	-1.335	0.135	2.998	0.000
WP_APOPTOSIS_MODULATION_AND_SIGNALING	84	-1.337726	0.11764706	1.405619	0.1026694
WP_METABOLIC_PATHWAY_OF_LDL_HDL_AND_TG_INCLUDING_DISEASES	16	-1.347816	0.14653465	-1.202224	0.22908367
WP_RAS_AND_BRADYKININ_PATHWAYS_IN_COVID19	28	-1.348379	0.13163064	-1.066777	0.34901962
WP_FAMILIAL_PARTIAL_LIPODYSTROPHY	30	-1.349332	0.12774451	-1.884781	0.011952192
WP_INTERACTIONS_BETWEEN_LOXL4_AND_OXIDATIVE_STRESS_PATHWAY	18	-1.349703	0.14723927	-1.168711	0.24482109
WP_GASTRIC_CANCER_NETWORK_1	25	-1.350	0.137	2.660	0.000
WP_NANOPARTICLE_TRIGGERED_REGULATED_NECROSIS	12	-1.35173	0.13412228	-0.711594	0.84599155
KEGG_SPHINGOLIPID_METABOLISM	38	-1.35322	0.14	-0.79704	0.71543086
WP_ARSENIC_METABOLISM_AND_REACTIVE_OXYGEN_SPECIES_GENERATION	10	-1.354335	0.14910537	1.050198	0.38387716
WP_SPHINGOLIPID_METABOLISM_IN_SENESCENCE	28	-1.354502	0.13983051	0.765446	0.740519
KEGG_RNA_POLYMERASE	27	-1.357	0.116	2.771	0.000
WP_PROSTAGLANDIN_AND_LEUKOTRIENE_METABOLISM_IN_SENESCENCE	31	-1.357294	0.12941177	-1.936814	0.00591716
WP_PURINE_METABOLISM	13	-1.360223	0.12992126	1.050784	0.39034206
WP_16P112_DISTAL_DELETION_SYNDROME	31	-1.369527	0.11812627	-0.57245	0.9692308
WP_STATIN_INHIBITION_OF_CHOLESTEROL_PRODUCTION	29	-1.370985	0.1027668	-0.878693	0.592
KEGG_PROXIMAL_TUBULE_BICARBONATE_RECLAMATION	22	-1.372467	0.10699589	-2.018792	0.004
WP_CHOLESTEROL_BIOSYNTHESIS_PATHWAY	15	-1.375	0.125	1.902	0.016

WP_NSP1_FROM_SARSCOV2_INHIBITS_TRANSLATION_INITIATION_IN_THE_HOST_CELL	16	-1.376	0.132	2.382	0.000
KEGG_PRIMARY_BILE_ACID_BIOSYNTHESIS	16	-1.377437	0.12896825	-0.993445	0.43907562
WP_VITAMIN_D_IN_INFLAMMATORY_DISEASES	22	-1.378431	0.12550607	-1.501154	0.08264463
KEGG_OOCYTE_MEIOSIS	108	-1.386	0.113	1.643	0.036
WP_MAPK_SIGNALING_PATHWAY	244	-1.38809	0.11605416	-3.191005	0
KEGG_GLYCOSPHINGOLIPID_BIOSYNTHESIS_LACTO_AND_NEOLACTO_SERIES	22	-1.389321	0.11553785	-1.056457	0.35416666
HALLMARK_REACTIVE_OXYGEN_SPECIES_PATHWAY	49	-1.394003	0.12062257	1.236219	0.19238476
WP_LEUKOTRIENE_METABOLIC_PATHWAY	13	-1.395492	0.10351563	-0.870762	0.5973535
WP_LIPID_METABOLISM_IN_SENESCENT_CELLS	20	-1.398925	0.11434511	1.019377	0.41015625
WP_FATTY_ACID_BETAOXIDATION	33	-1.400037	0.08661418	-2.001402	0.003853565
WP_FERROPTOSIS	61	-1.400315	0.10019268	1.417729	0.100196466
WP_MEASLES_VIRUS_INFECTION	127	-1.412	0.110	1.823	0.008
KEGG_PROGESTERONE_MEDIATED_OOCYTE_MATURATION	83	-1.414406	0.10097087	1.533916	0.06092437
WP_EXTRACELLULAR_VESICLES_IN_THE_CROSSTALK_OF_CARDIAC_CELLS	19	-1.41656	0.09829868	-0.867121	0.61627907
WP_ADIPOGENESIS	129	-1.418358	0.10357816	-3.407314	0
WP_DEVELOPMENT_AND_HETEROGENEITY_OF_THE_ILC_FAMILY	32	-1.419655	0.09741551	0.715009	0.83233535
HALLMARK_XENOBIOTIC_METABOLISM	195	-1.420358	0.10121457	-2.03276	0.002016129
WP_CHOLESTEROL_METABOLISM_WITH_BLOCH_AND_KANDUTSCHRUSSELL_PATHWAYS	47	-1.423047	0.1010101	1.20594	0.23706897
WP_NEPHROGENESIS	18	-1.423682	0.09503239	-1.198617	0.23274162
WP_DIFFERENTIATION_OF_WHITE_AND_BROWN_ADIPOCYTE	25	-1.425803	0.1027668	-2.121026	0.003984064
WP_METABOLIC_REPROGRAMMING_IN_PANCREATIC_CANCER	42	-1.430909	0.09714286	-0.735875	0.8023952
WP_NANOPARTICLEMEDIATED_ACTIVATION_OF_RECEPTOR_SIGNALING	28	-1.434623	0.0851927	-1.567295	0.05631068
WP_LEPTIN_AND_ADIPONECTIN	10	-1.434746	0.08232932	-1.53502	0.044315994
WP_FATTY_ACID_TRANSPORTERS	18	-1.435927	0.07942974	0.659945	0.8798521
HALLMARK_PANCREAS_BETA_CELLS	39	-1.442081	0.07632094	0.949706	0.5395257
WP_GPCRS_OTHER	69	-1.450079	0.08333334	-1.999795	0.006
WP_IRINOTECAN_PATHWAY	9	-1.451223	0.09484536	-1.087235	0.36419752

HALLMARK_UV_RESPONSE_UP	156	-1.453219	0.08300395	1.164576	0.25296444
WP_SYNTHESIS_OF_CERAMIDES_AND_1DEOXYCERAMIDES	22	-1.453267	0.10472279	-1.13303	0.2661448
WP_DISORDERS_OF_BILE_ACID_SYNTHESIS_AND_BILIARY_TRANSPORT	19	-1.455075	0.0824295	-0.943581	0.5285996
WP_LIPID_PARTICLES_COMPOSITION	9	-1.460295	0.0703125	-1.007938	0.41501975
WP_APOPTOSIS	83	-1.469	0.073	1.556	0.038
WP_TP53_NETWORK	19	-1.481069	0.07228915	1.593488	0.05172414
KEGG_PRION_DISEASES	34	-1.491604	0.06175299	-1.361291	0.13424124
WP_B_CELL_RECEPTOR_SIGNALING_PATHWAY	97	-1.502483	0.07739308	-1.001846	0.41897234
WP_HOSTPATHOGEN_INTERACTION_OF_HUMAN_CORONAVIRUSES_APOPTOSIS	21	-1.508	0.078	1.922	0.012
WP_MITOCHONDRIAL_COMPLEX_III_ASSEMBLY	14	-1.508	0.058	1.812	0.006
WP_MEVALONATE_ARM_OF_CHOLESTEROL_BIOSYNTHESIS_PATHWAY	13	-1.512	0.072	1.862	0.006
WP_CYTOSOLIC_DNASENSING_PATHWAY	63	-1.528	0.060	1.963	0.012
WP_CELL_CYCLE	119	-1.528	0.053	3.651	0.000
WP_GENETIC_CAUSES_OF_PSVDINCPH	37	-1.529268	0.06990291	-1.368864	0.12403101
WP_T_CELL_RECEPTOR_AND_COSTIMULATORY_SIGNALING	28	-1.535168	0.05192308	-1.147284	0.28031808
KEGG_AMINO_SUGAR_AND_NUCLEOTIDE_SUGAR_METABOLISM	43	-1.540946	0.05976096	0.568704	0.9586777
WP_EICOSANOID_METABOLISM_VIA_CYCLOOXYGENASES_COX	31	-1.541453	0.05383023	-1.625888	0.036585364
WP_OVERVIEW_OF_NANOPARTICLE_EFFECTS	17	-1.546262	0.062	-1.48408	0.07910751
WP_ACE_INHIBITOR_PATHWAY	17	-1.557427	0.05439331	-1.68255	0.026859503
WP_PROSURVIVAL_SIGNALING_OF_NEUROPROTECTIN_D1	15	-1.564359	0.0524109	1.321127	0.15369649
WP_IL10_ANTIINFLAMMATORY_SIGNALING_PATHWAY	12	-1.585597	0.05363985	-0.631378	0.9030303
WP_IL3_SIGNALING_PATHWAY	48	-1.592106	0.05294118	-1.283643	0.15
WP_LEUKOCYTEINTRINSIC_HIPPO_PATHWAY_FUNCTIONS	33	-1.609507	0.05316974	-1.283398	0.15650406
WP_PURINERGIC_SIGNALING	31	-1.615333	0.04216867	-1.766186	0.02173913
WP_ACQUIRED_PARTIAL_LIPODYSTROPHY_BARRAQUERSIMONS_SYNDROME	10	-1.623966	0.0464135	-0.798175	0.73307544

WP_HAIR_FOLLICLE_DEVELOPMENT_CYTODIFFERENTIATION_PART_3_OF_3	87	-1.625	0.035	1.780	0.026
WP_MIR5093P_ALTERATION_OF_YAP1ECM_AXIS	17	-1.625649	0.03861004	-2.031486	0.006
WP_NONGENOMIC_ACTIONS_OF_125_DIHYDROXYVITAMIN_D3	70	-1.628863	0.03754941	-1.44148	0.088176355
WP_CONTROL_OF_IMMUNE_TOLERANCE_BY_VASOACTIVE_INTESTINAL_PEPTIDE	13	-1.655029	0.04320988	0.59816	0.9611651
WP_MYOMETRIAL_RELAXATION_AND_CONTRACTION_PATHWAYS	154	-1.661303	0.03219316	-2.636031	0
WP_SELENIUM_MICRONUTRIENT_NETWORK	83	-1.665283	0.03822938	0.596985	0.9560878
WP_FBXL10_ENHANCEMENT_OF_MAPERK_SIGNALING_IN_DIFFUSE_LARGE_BCELL_LYMPHOMA	30	-1.666	0.036	1.606	0.044
WP_G1_TO_S_CELL_CYCLE_CONTROL	64	-1.667	0.033	3.600	0.000
WP_CHOLESTEROL_SYNTHESIS_DISORDERS	17	-1.670	0.026	2.022	0.004
WP_CYTOKINES_AND_INFLAMMATORY_RESPONSE	24	-1.67303	0.03339882	0.665765	0.88645416
KEGG_CELL_CYCLE	123	-1.676	0.039	3.769	0.000
KEGG_FATTY_ACID_METABOLISM	39	-1.676884	0.0362173	-1.761353	0.011904762
KEGG_CALCIUM_SIGNALING_PATHWAY	173	-1.676936	0.03082852	-3.813372	0
HALLMARK_PI3K_AKT_MTOR_SIGNALING	105	-1.686597	0.02191235	-0.75112	0.77281946
WP_PROSTAGLANDIN_SIGNALING	29	-1.700658	0.01964637	1.003659	0.4271255
WP_TYPE_I_INTERFERON_INDUCTION_AND_SIGNALING_DURING_SARSCOV2_INFECTION	31	-1.711305	0.03042596	-1.067099	0.32485324
KEGG_O_GLYCAN_BIOSYNTHESIS	29	-1.718842	0.02439024	-1.250727	0.1617357
WP_RETINOBLASTOMA_GENE_IN_CANCER	87	-1.723	0.022	6.370	0.000
WP_BIOGENIC_AMINE_SYNTHESIS	15	-1.722625	0.01730769	-0.892405	0.6069959
KEGG_EPITHELIAL_CELL_SIGNALING_IN_HELICOBACTER_PYLORI_INFECTION	66	-1.726152	0.01408451	1.261561	0.19455253
KEGG_DILATED_CARDIOMYOPATHY	89	-1.734309	0.02123552	-3.419128	0
WP_TOLLLIKE_RECEPTOR_SIGNALING_RELATED_TO_MYD88	31	-1.734575	0.0268714	-0.887611	0.5888031
WP_CANONICAL_AND_NONCANONICAL_TGFB_SIGNALING	17	-1.760273	0.01351351	-2.389458	0
WP_TH17_CELL_DIFFERENTIATION_PATHWAY	69	-1.760979	0.01803607	-1.208745	0.20970874

WP_THYMIC_STROMAL_LYMPHOPOIETIN_TSLP_SIGNALING_PATHWAY	46	-1.761146	0.03100775	1.152433	0.24844721
KEGG_AMYOTROPHIC_LATERAL_SCLEROSIS_ALS	53	-1.763606	0.02173913	-1.273439	0.16893204
WP_NUCLEOTIDE_GPCRS	11	-1.771425	0.02028398	-1.172643	0.24375
WP_VEGFAVEGFR2_SIGNALING_PATHWAY	423	-1.776951	0.02816901	-3.55093	0
WP_ULCERATIVE_COLITIS_SIGNALING	18	-1.78092	0.01165049	-0.889908	0.5719844
KEGG_HYPERTROPHIC_CARDIOMYOPATHY_HCM	83	-1.784451	0.03448276	-3.103913	0
WP_RAS_SIGNALING	179	-1.784803	0.03285421	-2.030033	0.01183432
KEGG_JAK_STAT_SIGNALING_PATHWAY	140	-1.788849	0.01239669	-1.443892	0.092929296
WP_TCA_CYCLE_AND_DEFICIENCY_OF_PYRUVATE_DEHYDROGENASE_COMPLEX_PDHC	16	-1.804129	0.01945525	-1.856232	0.018255578
WP_EICOSANOID_METABOLISM_VIA_LIPOOXYGENASES_LOX	29	-1.806549	0.00795229	-1.41025	0.10865191
HALLMARK_G2M_CHECKPOINT	198	-1.821	0.006	7.075	0.000
WP_SPHINGOLIPID_PATHWAY	30	-1.824367	0.01919386	-1.347768	0.13226452
WP_RALA_DOWNSTREAM_REGULATED_GENES	12	-1.828948	0.01221996	1.305307	0.16283925
KEGG_MAPK_SIGNALING_PATHWAY	264	-1.830153	0.00590551	-2.946141	0
WP_CANCER_IMMUNOTHERAPY_BY_CTLA4_BLOCKADE	13	-1.845241	0.01380671	-0.926971	0.5338491
KEGG_SNARE_INTERACTIONS_IN_VESICULAR_TRANSPORT	35	-1.87029	0.01859504	-0.627736	0.9234043
WP_MODULATORS_OF_TCR_SIGNALING_AND_T_CELL_ACTIVATION	61	-1.87284	0.00380228	1.455748	0.09330629
WP_REGULATORY_CIRCUITS_OF_THE_STAT3_SIGNALING_PATHWAY	78	-1.882868	0.00934579	-2.11867	0.004
WP_MYD88_DISTINCT_INPUTOUTPUT_PATHWAY	17	-1.896363	0.01046025	-1.158669	0.28305784
WP_PPAR_SIGNALING_PATHWAY	66	-1.904959	0.01190476	-1.834395	0.015217391
KEGG_PYRIMIDINE_METABOLISM	95	-1.912	0.010	3.715	0.000
WP_INTERACTIONS_BETWEEN_IMMUNE_CELLS_AND_MICRORNAS_IN_TUMOR_MICROENVIRONMENT	38	-1.930787	0.00375235	-1.236568	0.19153225
WP_ALZHEIMERS_DISEASE_AND_MIRNA_EFFECTS	267	-1.946351	0.00608519	1.464519	0.0720339
WP_COMPLEMENT_SYSTEM_IN_NEURONAL_DEVELOPMENT_AND_PLASTICITY	97	-1.947813	0.00627615	-1.642783	0.04191617
KEGG_HISTIDINE_METABOLISM	28	-1.949408	0.01028807	-1.064922	0.375
HALLMARK_APICAL_JUNCTION	197	-1.950784	0.00584795	-3.447031	0

WP_COVID19_ADVERSE_OUTCOME_PATHWAY	13	-1.966832	0.00606061	-0.916063	0.5567227
HALLMARK_DNA_REPAIR	148	-1.971	0.006	4.355	0.000
WP_PI3KAKT_SIGNALING_PATHWAY	322	-1.974555	0.00788955	-3.354543	0
KEGG_APOPTOSIS	82	-1.983193	0.002	-1.356806	0.11363637
KEGG_VEGF_SIGNALING_PATHWAY	75	-1.98576	0	-1.879636	0.011406845
KEGG_FC_GAMMA_R_MEDIATED_PHAGOCYTOSIS	95	-1.995468	0.00588235	-1.985742	0.007633588
WP_TNFRELATED_WEAK_INDUCER_OF_APOPTOSIS_TWEAK_SIGNALING_PATHWAY	42	-1.998275	0	-0.942217	0.5132383
WP_HEMATOPOIETIC_STEM_CELL_DIFFERENTIATION	57	-2.000183	0.004158	-0.793913	0.7322515
KEGG_PPAR_SIGNALING_PATHWAY	68	-2.001319	0.00191571	-1.822153	0.014285714
WP_TCA_CYCLE_AKA_KREBS_OR_CITRIC_ACID_CYCLE	18	-2.002389	0.00205761	-1.677405	0.032388665
HALLMARK_CHOLESTEROL_HOMEOSTASIS	74	-2.012392	0	0.82448	0.7131931
KEGG_ECM_RECEPTOR_INTERACTION	83	-2.018302	0.00982318	-4.444363	0
KEGG_CARDIAC_MUSCLE_CONTRACTION	72	-2.038196	0	-1.356292	0.12139918
KEGG_PROTEIN_EXPORT	23	-2.04753	0.00208768	1.562149	0.051652893
WP_PHOTODYNAMIC_THERAPYINDUCED_UNFOLDED_PROTEIN_RESPONSE	27	-2.050576	0.00197239	1.365821	0.11691023
WP_IMMUNE_RESPONSE_TO_TUBERCULOSIS	23	-2.060197	0	0.860097	0.6032389
KEGG_CITRATE_CYCLE_TCA_CYCLE	30	-2.071897	0.0043573	-2.029599	0.008474576
WP_COMPLEMENT_ACTIVATION	21	-2.082077	0.00401606	-1.691709	0.035363458
WP_PYRIMIDINE_METABOLISM	81	-2.089	0.000	3.674	0.000
WP_TCELL_ANTIGEN_RECEPTOR_TCR_PATHWAY_DURING_STAPHYLOCOCCUS_AUREUS_INFECTION	62	-2.101379	0.00203252	0.897884	0.5527426
WP_MACROPHAGE_MARKERS	9	-2.102489	0	-1.111173	0.31176472
WP_FOCAL_ADHESION	196	-2.115154	0.00406504	-4.232165	0
WP_FOCAL_ADHESION_PI3KAKTMTORSIGNALING_PATHWAY	300	-2.117725	0	-3.623941	0
WP_LUNG_FIBROSIS	58	-2.122622	0	-1.764224	0.027944112
WP_LDL_INFLUENCE_ON_CD14_AND_TLR4	24	-2.128374	0.0020284	-0.671397	0.8757638
KEGG_OTHER_GLYCAN_DEGRADATION	15	-2.131606	0.00197628	-1.258153	0.18762475
WP_PHOTODYNAMIC_THERAPYINDUCED_NFKB_SURVIVAL_SIGNALING	34	-2.134908	0.00623701	-0.732578	0.8031496

WP_TYPE_II_INTERFERON_SIGNALING	33	-2.136261	0	-1.191471	0.24
WP_ACUTE_VIRAL_MYOCARDITIS	84	-2.162229	0.00582524	-0.59541	0.9506903
WP_OXIDATIVE_DAMAGE_RESPONSE	40	-2.167383	0	1.396067	0.12210526
WP_IL18_SIGNALING_PATHWAY	266	-2.171566	0.00594059	-0.684874	0.86915886
HALLMARK_FATTY_ACID_METABOLISM	156	-2.173474	0.00399202	-1.659648	0.04477612
WP_ALZHEIMERS_DISEASE	254	-2.179	0.002	1.722	0.013
KEGG_COMPLEMENT_AND_COAGULATION_CASCADES	65	-2.184336	0.00203666	-2.173697	0.003929273
WP_TCELL_ACTIVATION_SARSCOV2	87	-2.190748	0	-1.449997	0.08514851
KEGG_AUTOIMMUNE_THYROID_DISEASE	37	-2.192	0.000	1.809	0.014
WP_TOLLLIKE_RECEPTOR_SIGNALING_PATHWAY	94	-2.199504	0.00215983	-1.194604	0.23780487
KEGG_REGULATION_OF_ACTIN_CYTOSKELETON	209	-2.226989	0	-3.173115	0
WP_FOXP3_IN_COVID19	15	-2.230166	0.00197628	-1.245508	0.2251407
WP_MIRNA_TARGETS_IN_ECM_AND_MEMBRANE_RECEPTORS	30	-2.241794	0	-2.342963	0.001949318
WP_MITOCHONDRIAL_COMPLEX_IV_ASSEMBLY	30	-2.245	0.006	2.811	0.000
KEGG_LEUKOCYTE_TRANSENDOTHELIAL_MIGRATION	112	-2.253778	0.00200803	-2.241869	0
KEGG_PATHOGENIC_ESCHERICHIA_COLI_INFECTION	52	-2.270275	0	0.690237	0.85655737
WP_CANCER_IMMUNOTHERAPY_BY_PD1_BLOCKADE	22	-2.270325	0.0020284	1.169164	0.246
KEGG_DNA_REPLICATION	35	-2.272	0.002	5.244	0.000
WP_PATHOGENIC_ESCHERICHIA_COLI_INFECTION	52	-2.279776	0.00199203	0.695467	0.84189725
WP_SPINAL_CORD_INJURY	113	-2.284422	0	-1.210771	0.218107
HALLMARK_ANGIOGENESIS	36	-2.291318	0	-2.956393	0
KEGG_FC_EPSILON_RI_SIGNALING_PATHWAY	78	-2.299761	0	-1.687541	0.015810277
KEGG_TOLL_LIKE_RECEPTOR_SIGNALING_PATHWAY	93	-2.300368	0	-1.254637	0.20486815
HALLMARK_COAGULATION	137	-2.305969	0.00392927	-2.595021	0
KEGG_FOCAL_ADHESION	197	-2.309053	0	-4.900367	0
WP_INFLAMMATORY_BOWEL_DISEASE_SIGNALING	43	-2.314163	0	-0.604618	0.92673266
WP_SARSCOV2_INNATE_IMMUNITY_EVASION_AND_CELLSPECIFIC_IMMUNE_RESPONSE	61	-2.315424	0	-0.768793	0.7471698
WP_2Q13_COPY_NUMBER_VARIATION_SYNDROME	58	-2.318	0.002	2.058	0.006

WP_COMPLEMENT_AND_COAGULATION_CASCADES	57	-2.32505	0.00196464	-2.01916	0.008179959
WP_NUCLEOTIDEBINDING_OLIGOMERIZATION_DOMAIN_NOD_PATHWAY	37	-2.335131	0.0021645	1.029574	0.4279919
WP_GENES_ASSOCIATED_WITH_THE_DEVELOPMENT_OF_RHEUMATOID_ARTHRITIS	16	-2.347329	0	-1.169167	0.25050506
HALLMARK_ADIPOGENESIS	200	-2.35532	0	-2.862732	0
WP_EBOLA_VIRUS_INFECTION_IN_HOST	118	-2.357229	0	-1.943082	0.009398496
HALLMARK_INTERFERON_ALPHA_RESPONSE	95	-2.372	0.000	2.181	0.004
WP_MATRIX_METALLOPROTEINASES	29	-2.407505	0	-1.150074	0.2725451
WP_CELLS_AND_MOLECULES_INVOLVED_IN_LOCAL_ACUTE_INFLAMMATORY_RESPONSE	16	-2.429338	0	-1.876064	0.010504202
HALLMARK_APOPTOSIS	161	-2.495417	0	-0.95992	0.50625
KEGG_CELL_ADHESION_MOLECULES_CAMS	119	-2.498343	0.00206186	-2.078204	0.004081633
KEGG_SYSTEMIC_LUPUS_ERYTHEMATOSUS	113	-2.502057	0.00601202	1.424371	0.103238866
WP_PEPTIDE_GPCRS	73	-2.514609	0	-1.946318	0.00845666
WP_PARKINUBIQUITIN_PROTEASOMAL_SYSTEM_PATHWAY	66	-2.528	0.000	2.272	0.000
WP_INFLAMMATORY_RESPONSE_PATHWAY	30	-2.534781	0	-1.246405	0.20675944
WP_PROSTAGLANDIN_SYNTHESIS_AND_REGULATION	44	-2.602268	0	-1.058904	0.36713997
WP_DNA_REPLICATION	41	-2.616	0.000	5.063	0.000
KEGG_B_CELL_RECEPTOR_SIGNALING_PATHWAY	75	-2.637628	0	-0.995799	0.45974576
WP_HYPERTROPHY_MODEL	20	-2.664085	0	0.863201	0.6289308
KEGG_ANTIGEN_PROCESSING_AND_PRESENTATION	60	-2.667	0.000	2.259	0.002
KEGG_NOD_LIKE_RECEPTOR_SIGNALING_PATHWAY	56	-2.684653	0	0.838221	0.64785993
WP_VITAMIN_D_RECEPTOR_PATHWAY	167	-2.685894	0	0.991984	0.4473684
KEGG_ASTHMA	23	-2.693734	0	1.408358	0.109561756
WP_SMALL_LIGAND_GPCRS	19	-2.745402	0	-1.274417	0.19253439
HALLMARK_MTORC1_SIGNALING	200	-2.748	0.000	3.435	0.000
KEGG_NEUROACTIVE_LIGAND_RECEPTOR_INTERACTION	251	-2.768598	0	-2.308869	0
WP_IL1_AND_MEGAKARYOCYTES_IN_OBESITY	24	-2.769209	0	-1.224735	0.20734341

WP_PLATELETMEDIATED_INTERACTIONS_WITH_VASCULAR_AND_CIRCULATING_CELLS	17	-2.844696	0	-1.215372	0.22154471
KEGG_VIRAL_MYOCARDITIS	62	-2.871899	0	-2.396529	0
KEGG_LYSOSOME	119	-2.911807	0	-2.191565	0
WP_STRIATED_MUSCLE_CONTRACTION_PATHWAY	38	-2.914348	0	-3.050186	0
WP_BURN_WOUND_HEALING	103	-2.923809	0	-1.779004	0.02631579
HALLMARK_E2F_TARGETS	200	-2.932	0.000	9.903	0.000
KEGG_T_CELL_RECEPTOR_SIGNALING_PATHWAY	108	-2.955981	0	-1.045734	0.38065845
KEGG_PRIMARY_IMMUNODEFICIENCY	34	-2.964	0.000	1.894	0.008
WP_INTERACTIONS_OF_NATURAL_KILLER_CELLS_IN_PANCREATIC_CANCER	22	-2.966437	0	1.511635	0.05367793
WP_PATHOGENESIS_OF_SARSCOV2_MEDIATED_BY_NSP9NSP10_COMPLEX	17	-2.984688	0	1.494743	0.08080808
KEGG_GRAFT_VERSUS_HOST_DISEASE	28	-2.993	0.000	1.640	0.041
WP_GPCRS_CLASS_A_RHODOPSINLIKE	216	-3.026269	0	-3.124962	0
WP_CHEMOKINE_SIGNALING_PATHWAY	160	-3.028039	0	-2.773611	0
KEGG_ALLOGRAFT_REJECTION	29	-3.061	0.000	1.809	0.018
WP_SELECTIVE_EXPRESSION_OF_CHEMOKINE_RECEPTORS_DURING_TCELL_POLARIZATION	28	-3.074596	0	-1.060799	0.36363637
KEGG_LEISHMANIA_INFECTION	63	-3.098776	0	1.012616	0.44554454
KEGG_INTESTINAL_IMMUNE_NETWORK_FOR_IGA_PRODUCTION	42	-3.116205	0	-1.500287	0.073267326
KEGG_TYPE_I_DIABETES_MELLITUS	35	-3.137	0.000	1.858	0.010
WP_TCELL_RECEPTOR_SIGNALING_PATHWAY	90	-3.152343	0	-1.536901	0.034552846
KEGG_HEMATOPOIETIC_CELL_LINEAGE	79	-3.17099	0	-2.606453	0
WP_COMPLEMENT_SYSTEM	89	-3.226431	0	-2.620989	0
KEGG_CHEMOKINE_SIGNALING_PATHWAY	172	-3.262615	0	-2.871542	0
HALLMARK_TNFA_SIGNALING_VIA_NFKB	199	-3.285306	0	1.275217	0.16463415
WP_CYTOPLASMIC_RIBOSOMAL_PROTEINS	86	-3.288	0.000	7.352	0.000
HALLMARK_COMPLEMENT	190	-3.309468	0	-1.653212	0.040618956
HALLMARK_IL2_STAT5_SIGNALING	199	-3.372619	0	0.634943	0.9002079
HALLMARK_MYOGENESIS	200	-3.43423	0	-7.497739	0

WP_NETWORK_MAP_OF_SARSCOV2_SIGNALING_PATHWAY	202	-3.451431	0	-0.959307	0.4895238
HALLMARK_MYC_TARGETS_V1	198	-3.465	0.000	8.624	0.000
KEGG_RIBOSOME	84	-3.506	0.000	8.007	0.000
KEGG_HUNTINGTONS_DISEASE	167	-3.537	0.000	3.442	0.000
KEGG_ALZHEIMERS_DISEASE	155	-3.550	0.000	1.785	0.019
KEGG_NATURAL_KILLER_CELL_MEDIATED_CYTOTOXICITY	107	-3.551474	0	-1.783768	0.01764706
WP_OVERVIEW_OF_PROINFLAMMATORY_AND_PROFIBROTIC_MEDIATORS	102	-3.585	0.000	1.608	0.032
HALLMARK_EPITHELIAL_MESENCHYMAL_TRANSITION	196	-3.605715	0	-5.308645	0
WP_MICROGLIA_PATHOGEN_PHAGOCYTOSIS_PATHWAY	39	-3.629534	0	-0.94695	0.49309665
WP_OXIDATIVE_PHOSPHORYLATION	52	-3.653	0.000	4.319	0.000
WP_MITOCHONDRIAL_COMPLEX_I_ASSEMBLY_MODEL_OXPHOS_SYSTEM	49	-3.673	0.000	3.438	0.000
HALLMARK_IL6_JAK_STAT3_SIGNALING	86	-3.696527	0	-1.572839	0.034979425
WP_NONALCOHOLIC_FATTY_LIVER_DISEASE	147	-3.706	0.000	1.766	0.017
HALLMARK_KRAS_SIGNALING_UP	199	-3.97263	0	-2.003536	0.003795066
KEGG_PARKINSONS_DISEASE	111	-4.011	0.000	3.948	0.000
WP_ALLOGRAFT_REJECTION	79	-4.059584	0	-1.251287	0.2055336
KEGG_OXIDATIVE_PHOSPHORYLATION	115	-4.142	0.000	4.470	0.000
WP_EXTRAFOLLICULAR_B_CELL_ACTIVATION_BY_SARSCOV2	67	-4.14732	0	1.257975	0.18787879
KEGG_CYTOKINE_CYTOKINE_RECEPTOR_INTERACTION	233	-4.166408	0	-2.338732	0
WP_TYROBP_CAUSAL_NETWORK_IN_MICROGLIA	58	-4.390703	0	-2.19338	0
WP_ELECTRON_TRANSPORT_CHAIN_OXPHOS_SYSTEM_IN_MITOCHONDRIA	88	-4.468	0.000	4.300	0.000
WP_PROTEASOME_DEGRADATION	60	-4.544	0.000	4.027	0.000
HALLMARK_INTERFERON_GAMMA_RESPONSE	197	-4.717	0.000	2.193	0.000
KEGG_PROTEASOME	44	-4.883	0.000	4.342	0.000
HALLMARK_OXIDATIVE_PHOSPHORYLATION	198	-5.013	0.000	3.256	0.000
HALLMARK_INFLAMMATORY_RESPONSE	198	-5.285856	0	-1.618838	0.038306452
HALLMARK_ALLOGRAFT_REJECTION	198	-7.012	0.000	2.252	0.000

Supplemental table S3. Proteins altered in genome-edited tumors on MMTV-Wnt1 vs on Wild-type

Category	Protein name	*Kras*- mutated tumors on Wnt1 vs. WT			*Pik3ca*- mutated tumors on Wnt1 vs. WT		
		T-test	Fold	FDR	T-test	Fold	FDR
Unique to Kras-mutated tumors	Akt	5.2E-03	2.6	0.039			
	Bax	1.9E-02	2.5	0.101			
	p-Chk1(S345)	2.2E-02	15.4	0.112			
	p-EGFR(Y1173)	2.0E-02	1.3	0.103			
	HER3/ErbB3	2.8E-02	1.5	0.120			
	Lipocalin-1	1.5E-02	1.6	0.085			
	p-PTEN(S380)	3.8E-02	1.8	0.153			
	PHF8	3.8E-04	1.9	0.005			
	ASH2	6.7E-05	3.8	0.001			
	c-Jun	3.4E-03	2.4	0.030			
	CRSP1-TRAP220	1.6E-05	2.1	0.001			
	p300	9.9E-05	3.1	0.002			
	Raptor	1.3E-02	1.6	0.078			
	14-3-3zeta,gamma,eta	1.2E-02	0.6	0.074			
	p-Akt(T308)	5.4E-03	0.1	0.040			
	ALK	1.3E-02	0.3	0.078			
	Annexin1	2.3E-02	0.4	0.110			
	p-Met(Y1234/1235)	1.7E-03	0.0	0.018			
	p-p44/42MAPK(Erk1/2)(T202/Y204)	3.2E-02	0.3	0.133			
	p-p53(S15)	2.3E-05	0.0	0.001			
	p-PDGFRb(Y751)	3.9E-02	0.5	0.156			
	Integrinb1	2.8E-02	0.0	0.123			
	Caspase-3(cleaved at Asp175)	7.6E-03	0.4	0.054			
	p-SHC(2431)(Y317)	1.7E-02	0.4	0.091			
	p-4E-BP1 (Thr37/46)	4.9E-02	0.4	0.185			
	p-ALK(Y1586)	2.7E-02	1.4	0.121	1.8E-03	1.8	0.015
	Bcl-xL	6.5E-05	2.3	0.001	8.9E-04	3.5	0.011
	E-Cadherin	3.4E-03	9.4	0.029	1.5E-03	6.6	0.013
	p-Beta-Catenin(S33/37/T41)	2.8E-02	34.9	0.122	1.2E-02	1.5	0.047
	c-Met	8.4E-03	2.5	0.056	1.4E-02	2.0	0.050
	p-c-Myc(T58)	1.2E-04	2.4	0.002	1.4E-02	1.7	0.050
	Ezh2	4.8E-05	7.7	0.001	4.6E-02	2.7	0.100
	FoxO1	1.9E-06	5.9	0.000	7.2E-05	5.9	0.004
	p-p27/KIP1(T198)	8.3E-03	2.2	0.057	6.0E-03	2.2	0.031
	PIAS1	4.1E-06	6.2	0.000	1.5E-02	3.7	0.051
	Slug	3.6E-03	3.0	0.030	8.5E-04	6.0	0.011
	Wnt5a/b	2.6E-03	2.5	0.025	1.7E-03	2.9	0.015
	Stat5a	2.3E-05	11.0	0.001	3.3E-02	5.6	0.086
	SOX9	6.8E-06	23.1	0.000	1.4E-02	14.1	0.050

Shared	CBP	3.1E-05	3.4	0.001	3.1E-02	3.6	0.083
	CtBP2	1.2E-06	10.6	0.000	2.0E-02	3.2	0.064
	DRIP130	1.7E-02	1.6	0.091	3.1E-02	1.4	0.083
	MED12	7.7E-05	2.8	0.001	2.2E-02	1.6	0.067
	PPP1R10	1.7E-04	4.9	0.002	3.7E-02	2.3	0.091
	Stat3	4.8E-03	2.6	0.038	4.3E-02	1.5	0.098
	p-TRAP220-MED1(T1457)	3.2E-04	3.2	0.004	6.0E-03	2.7	0.031
	Ki67	3.1E-02	3.2	0.128	3.1E-02	1.9	0.084
	PI3Kp110a	1.3E-03	2.4	0.014	8.4E-03	2.1	0.041
	Beta-Catenin	2.7E-03	3.5	0.025	1.4E-02	3.0	0.051
	c-Myc	2.4E-02	2.7	0.114	1.2E-02	2.8	0.047
	GATA-3	4.9E-02	3.5	0.187	4.9E-04	4.0	0.008
	NF-kB p65	9.1E-04	2.5	0.010	3.3E-03	2.7	0.023
	MEK6	8.7E-03	3.1	0.057	1.6E-04	5.5	0.005
	PRAS40	1.5E-02	1.9	0.085	4.5E-02	1.7	0.099
	p-Raptor (Ser792)	6.1E-04	2.1	0.007	1.4E-03	2.3	0.013
	PKM2	4.4E-02	0.7	0.171	1.3E-02	2.2	0.050
	PKM1/2	2.5E-02	0.7	0.116	3.3E-02	2.6	0.086
	p-Akt(S473)				2.0E-02	21.6	0.065
	p-ALK(Y1604)				4.9E-05	1.7	0.005
	p-AMPKa1(S485)				1.1E-02	2.2	0.046
	p-AMPKb1(S108)				1.3E-02	2.2	0.050
	p-AuroraA(T288)/B(T232)/C(T198)				4.4E-02	1.3	0.099
	p-Bad(S112)				4.8E-03	2.4	0.030
	p-Bad(S136)				2.8E-02	1.3	0.080
	p-Bad(S155)				5.2E-03	1.8	0.029
	Bak				1.4E-02	1.7	0.050
	N-Cadherin				2.4E-04	1.7	0.005
	p-Caveolin-1(Y14)				3.3E-02	1.6	0.086
	p-EGFR(Y1148)				2.9E-02	1.5	0.081
	p-EGFR(Y1173)				4.4E-02	1.1	0.100
	p-HER3/ErbB3(Y1197)				2.9E-03	1.6	0.021
	HDAC3				4.5E-03	1.5	0.028
	HDAC4				2.7E-02	1.5	0.079
	MEK1				6.5E-03	1.4	0.033
	p-MEK1/2(S217/221)				3.6E-02	1.7	0.091
	mTOR				9.4E-03	1.8	0.042
	p-mTOR(S2448)				1.1E-02	2.6	0.045
	Notch1				2.2E-04	4.0	0.005
	p-p27(T187)				1.7E-02	1.8	0.057
	p27/KIP1(C-term)				1.6E-04	2.7	0.006
	p44/42MAPK(Erk1/2)				2.0E-02	1.7	0.066
	p70S6K				1.1E-03	3.8	0.012
	p-PDGFRa(Y754)				4.9E-02	1.2	0.103
	p-PDK1(S241)				1.2E-03	2.2	0.013
	PTEN				2.7E-02	2.5	0.078

Unique to Pikc3a-muated Tumors	p-RafB(S445)				3.0E-03	2.4	0.021
	p-Smad2(S465/467)				3.7E-03	1.7	0.025
	SOCS1				5.0E-03	1.6	0.030
	SOCS3				4.9E-02	1.6	0.104
	p-Stat3(S727)				3.9E-03	2.0	0.025
	p-Stat5(Y694)				4.5E-02	2.0	0.100
	Stat6				9.4E-04	2.4	0.011
	CHAF1A				4.8E-02	1.2	0.104
	Cyclin C				3.6E-02	3.0	0.090
	p-Rb(S807/811)				2.3E-02	3.0	0.069
	p-Rb(S780)(C84F6)				5.7E-03	1.8	0.031
	p-FAK(Y576/577)				3.5E-02	1.4	0.090
	Integrinb4				9.9E-05	1.8	0.004
	PI3Kp85				5.4E-04	2.5	0.008
	AMPKa				5.3E-03	1.7	0.029
	Atg12				1.1E-02	1.7	0.047
	Beclin-1				2.5E-03	2.1	0.019
	LC3B				8.9E-03	2.0	0.042
	p-EGFR(Y845)				4.0E-02	1.3	0.094
	SRD5A1				7.1E-04	0.1	0.010
	HexokinaseII				2.0E-04	5.0	0.005
	p-FAK (Tyr397)				5.1E-03	1.8	0.030
	Aldehyde Dehydrogenase 2 (ALDH2)				9.2E-03	2.0	0.042
	IDH1				9.2E-04	2.4	0.011
	p-GSK-3a/b (Ser21/9)				2.0E-02	2.3	0.065
	HIF-1a				2.6E-02	2.4	0.077
	RB1				4.2E-02	1.6	0.099
	NCOA2				2.2E-04	1.3	0.005
	IKKb				1.0E-02	1.8	0.045
	GbetaL				4.6E-04	2.4	0.008
	ULK1				4.3E-05	2.3	0.009
	p-mTOR (Ser2481)				1.3E-03	1.7	0.013
	SQSTM1/p62				2.1E-02	3.0	0.065
	BRCA1				3.9E-02	0.5	0.093
	p-EGFR(S1046/1047)				3.7E-02	0.8	0.091
	p-EGFR(Y1045)				2.1E-03	0.1	0.017
	MMP-9				8.7E-03	0.7	0.042
	PDGFRb				4.9E-02	0.2	0.104
	p21				3.9E-02	0.3	0.092
	GLDC				6.5E-05	0.6	0.005
	SCD1				1.3E-02	0.5	0.051
	p-ATM (Ser1981)				2.6E-02	0.7	0.077

Data S1. (Separate file)
22804_unique_indel_filtered_no_trf.

Data S2. (Separate file)
Merged_pwmscan_mm10_up_to_5_mismatch_with_PAM_and_indel.

Data S3. (Separate file)
RPPA_WB

Data S4. (Separate file)
RNAseq_DiffEx_for_supplemental_011023
